# 5‐hydroxymethylcytosine features of portal venous blood predict metachronous liver metastases of colorectal cancer and reveal phosphodiesterase 4 as a therapeutic target

**DOI:** 10.1002/ctm2.70189

**Published:** 2025-02-16

**Authors:** Nuo Xu, Zhaoya Gao, Deyan Wu, Hangyu Chen, Zijian Zhang, Lei Zhang, Yuchen Wang, Xuyang Lu, Xu Yao, Xuelan Liu, Yi‐You Huang, Meiying Qiu, Sen Wang, Jinqiang Liang, Can Mao, Feng Zhang, Huimin Xu, Yujiao Wang, Xian Li, Zhexin Chen, Dandan Huang, Jingyi Shi, Wensheng Huang, Fuming Lei, Zeruo Yang, Long Chen, Chuan He, Haichuan Zhu, Hai‐Bin Luo, Jin Gu, Jian Lin

**Affiliations:** ^1^ Department of Pharmacy Peking University Third Hospital Beijing China; ^2^ Natural Medicine Institute of Zhejiang YangShengTang Co. Ltd. Hangzhou China; ^3^ Department of Gastrointestinal Surgery Peking University Shougang Hospital Beijing China; ^4^ Center for Precision Diagnosis and Treatment of Colorectal Cancer and Inflammatory Diseases Peking University Health Science Center Beijing China; ^5^ Key Laboratory of Tropical Biological Resources of Ministry of Education School of Pharmaceutical Sciences Hainan University Haikou Hainan China; ^6^ School of Pharmaceutical Sciences Sun Yat‐sen University Guangzhou China; ^7^ Department of Oncology Peking University Shougang Hospital Beijing China; ^8^ Key laboratory of Carcinogenesis & Translational Research (Ministry of Education), Department of Gastrointestinal Surgery III Peking University Cancer Hospital & Institute Beijing China; ^9^ Department of Chemistry Department of Biochemistry and Molecular Biology Howard Hughes Medical Institute The University of Chicago Chicago Illinois USA; ^10^ Institute of Biology and Medicine College of Life and Health Sciences Wuhan University of Science and Technology Wuhan China; ^11^ Peking University International Cancer Institute Peking University Beijing China; ^12^ Peking University Third Hospital Cancer Center Peking University Third Hospital Beijing China; ^13^ Synthetic and Functional Biomolecules Center Peking University Beijing China; ^14^ Key Laboratory of Tropical Biological Resources of Ministry of Education, Song Li's Academician Workstation of Hainan University, School of Pharmaceutical Sciences Hainan University Haikou China

**Keywords:** 5hmC‐Seal, Colorectal cancer, Metachronous liver metastases, PDE4D

## Abstract

Metachronous liver metastases (MLM) are characterised by high incidence and high mortality in clinical colorectal cancer treatment. Currently traditional clinical methods cannot effectively predict and prevent the occurrence of metachronous liver metastasis in colorectal cancer. Based on 5hmC‐Seal analysis of blood and tissue samples, this study found that portal venous blood was more relevant to tumour gDNA than peripheral blood. We performed a novel epigenetic liquid biopsy strategy using the 10 5hmC epigenetic alterations, to accurately distinguish MLM patients from patients without metastases. Among these epigenetic alterations, phosphodiesterase 4 (PDE4D) was highly increased in MLM patients and correlated with poor survival. Moreover, our studies demonstrated that PDE4D was a key metastasis‐driven target for drug development. Interfering with the function of PDE4D significantly repressed liver metastases. Similarly, roflumilast, a PDE4 inhibitor for chronic obstructive pulmonary disease (COPD) therapy, also inhibits liver metastases. Further studies indicate that blocking the function of PDE4D can affect CRC invasion through the HIF‐1α‐CCN2 pathway. To develop a more efficient PDE4 inhibitor and reduce the occurrence of adverse events, we also designed several new compounds based on 2‐arylbenzofurans and discovered lead L11 with potent affinity for PDE4D and significant suppression of liver metastases. In this work, our study provides a promising strategy for predicting metachronous liver metastasis and discovers L11 as a potential repurposed drug for inhibiting liver metastasis, which have the potential to benefit patients with CRC in the future.

**Key points:**

- 5hmC epigenetic markers derived from portal venous blood could accurately predict metachronous metastasis of colorectal cancer.
- PDE4D was a key metastasis‐driven target that promoted metachronous metastasis via the HIF‐1α‐CCN2 pathway.
- The newly synthesised compound L11 could specifically inhibit PDE4D and abolish metachronous metastasis of colorectal cancer without obvious toxic side effects.

## Graphical abstract

1. 5hmC epigenetic markers derived from portal venous blood could accurately predict metachronous metastasis of colorectal cancer.
2. PDE4D was a key metastasis‐driven target that promoted metachronous metastasis via the HIF‐1α‐CCN2 pathway.
3. The newly synthesised compound L11 could specifically inhibit PDE4D and abolish metachronous metastasis of colorectal cancer without obvious toxic side effects.

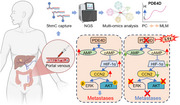

## 1 INTRODUCTION

Colorectal cancer (CRC) is the third most common incidence and momentous cause of cancer‐related mortality worldwide.^1^, ^2^ Metastases is the primary cause of CRC‐related death.^3^, ^4^ Approximately 20% of CRC patients are initially diagnosed with synchronous liver metastases (SLM), and another 20–25% of CRC patients will eventually develop metachronous liver metastases (MLM) after surgical resection of the primary tumour.^5^ Resection and chemotherapy are standard treatments for patients with metastases. However, surgery can only be performed in 10–20% of patients due to factors such as the location and size of the tumour and unresectable metastases. The 5‐year survival rate after surgery is as low as 30%.^6^, ^7^ Patients who are not eligible for surgery have an even worse prognosis.^8^ The shortcomings of standard therapies underscore the pressing need for advanced methods in MLM prediction and the identification of innovative therapeutic targets.

Early detection of high‐risk populations susceptible to MLM may provide opportunities for clinicians to adopt different treatment strategies and more intensive follow‐up plans to improve survival for patients with CRC.^9^ Genomic profiling can help select treatments that are more beneficial and less toxic for patients.^10^ Although previous research has been conducted to identify MLM warning biomarkers for patients with CRC after surgery, no efficacious early diagnosis method exists.^11^, ^12^, ^13^ Current CRC liquid biopsies rely on peripheral blood.^14^ However, physiologically, the portal venous carries approximately 75% of the total liver blood flow, connecting the colorectum and liver.^15^ Tumour cells metastasise and spread to the liver through portal venous blood.^16^, ^17^ The hepatic portal vein forms the core of the hepatic portal venous system. It is formed by the convergence of the superior mesenteric vein and the splenic vein, which occurs behind the junction between the head and body of the pancreas. During the process of blood sampling from the portal vein, it is difficult to draw blood with a puncture needle, and the success rate of blood collection is not high. Therefore, liquid biopsy based on portal venous blood has not been used in CRC. However, studies have shown that a higher number of circulating tumour cells (CTCs) are detected in portal venous blood compared to peripheral blood.^17^, ^18^, ^19^, ^20^ According to previous research on the spatial heterogeneity of CTC in different cycles, CTCs appear to be localised to the hepatic macrocirculation in patients with colorectal liver metastases.^21^ Besides, the expression levels of tumour markers such as CEA in portal venous blood are significantly higher than those in peripheral blood. Therefore, liquid biopsy based on portal venous blood may have more optimised effects on the early diagnosis of metastases and prognosis of treatment.

We have previously described the 5hmC features in circulating cell‐free DNA (cfDNA) from patients with solid tumours or haematological tumours, suggesting that 5hmC features have the potential to be specific biomarkers for human cancers arising from different tissue origins.^22^, ^23^, ^24^, ^25^, ^26^ Moreover, the difference of 5hmC abundance not only marks active demethylation but also serves as a stable DNA marker for gene expression.^27^ Hence, 5hmC features could serve as an efficient disease‐specific biomarker for a variety of human cancers and other diseases in early detection, diagnosis and prognosis.^23^, ^28^, ^29^, ^30^, ^31^, ^32^

Here, we performed a novel epigenetic liquid biopsy strategy using portal venous blood that could noninvasively predict CRC patients with metachronous liver metastases (MLM). Moreover, early‐stage MLM patients could be precisely predicted based on these differential 5hmC features. We further found cAMP‐specific 3′,5′‐cyclic phosphodiesterase 4D (PDE4D) as an essential therapeutic target for liver metastases of CRC and interfering the function of PDE4D could repress colorectal cancer metastases. Our research presents novel strategy, potential biomarkers, and leads to improve the therapeutic effect of CRC liver metastases.

## 2 RESULTS

### 2.1 Landscape of 5hmC profiles in cell‐free DNA and genomic DNA from colorectal cancer patients

**FIGURE 1:**
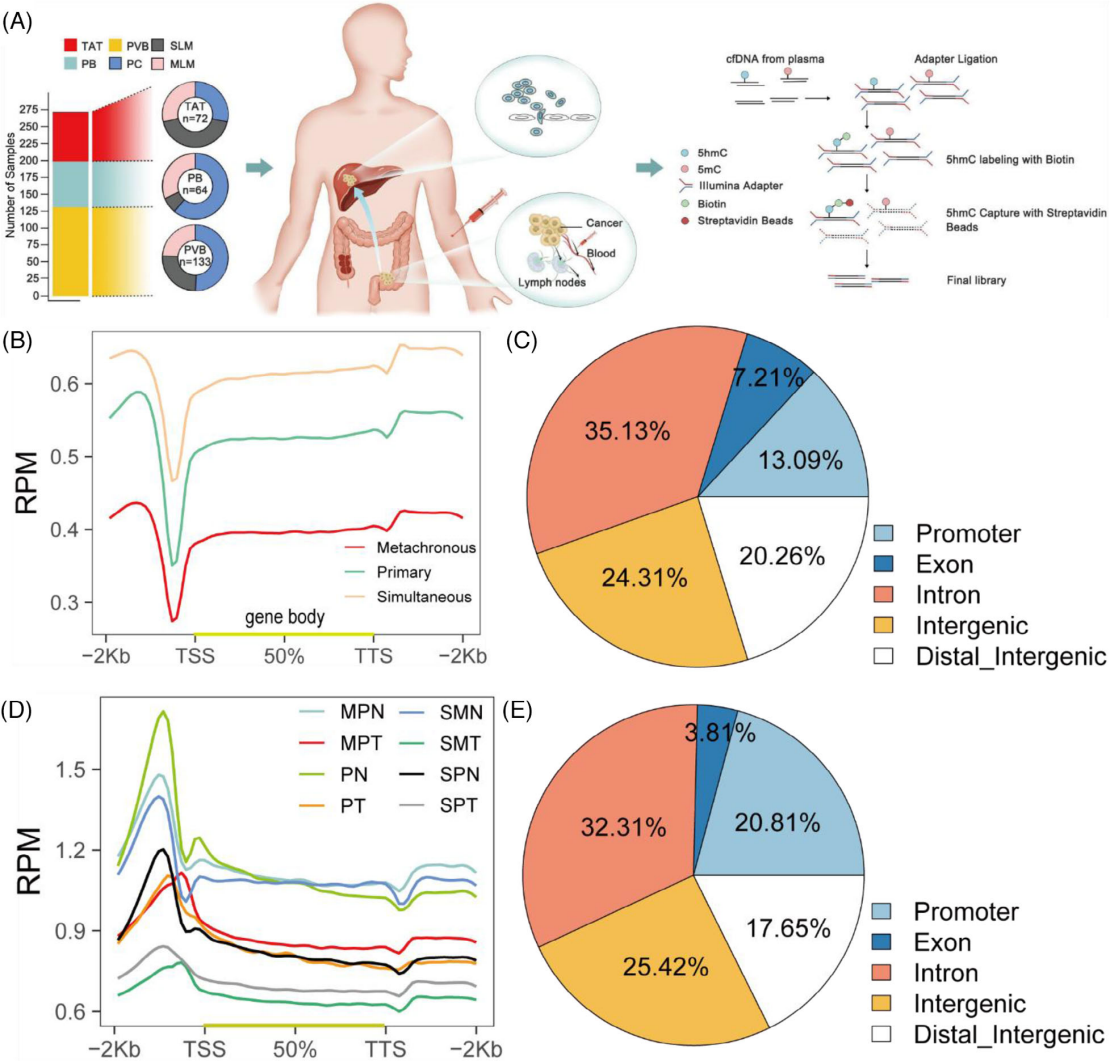
Characteristics of 5hmC distribution in plasma cfDNA and tissue gDNA of CRC patients. (A) Sample collection and 5hmC‐Seal workflow of 133 patients with CRC. (B) Distribution of normalised 5hmC read counts across the gene bodies in PC, MLM and SLM. Average numbers of hMRs located in different genomic regions. TSS, transcription start site; TTS, transcription termination site; RPM, Reads per million mapped reads. (C) Pie chart showing the percentage of 5hmC peaks in plasma cfDNA genomic features. The promoter regions are defined as 2 kb around the TSS. (D) Distribution of normalised 5hmC read counts across the gene bodies in gDNA of tissue samples. (E) Pie chart showing the percentage of 5hmC peaks in tissue gDNA genomic features. The promoter regions are defined as 2 kb around the TSS. MLM, metachronous liver metastasis of colorectal cancer; MPT, MLM primary tumour tissue and MPN; MLM primary adjacent normal tissue; PVB, Portal venous blood; PB, peripheral blood; PC, primary colorectal cancer; PT, PC tumour tissue; PN, PC adjacent normal tissue; SLM, simultaneous liver metastasis of colorectal cancer; SPT, SLM primary tumour tissue; SPN, SLM primary adjacent normal tissue; SMT, SLM metastatic tumour tissue; SMN, SLM metastatic adjacent normal tissue; TAT, tumour and adjacent tissue.

The portal venous blood collection period is after ligation of the arteries and veins in the colorectal tumour area and before tumour excision. After the blood collection needle is inserted into the veins in the tumour area, collection is performed. The volume of venous blood collected in each patient's tumour area ranges from 10 to 20 mL. After collection is completed, it is centrifuged and aliquoted within 30 min and stored in a –80°C freezer (Video S1). In total, 133 patients with colorectal cancer (CRC) participated in this study, of whom 70 were diagnosed with primary colorectal cancer (PC), 32 with metachronous liver metastasis of colorectal cancer (MLM), and 31 with simultaneous liver metastasis of colorectal cancer (SLM) (Table S1). Portal venous blood (PVB) was collected from a subset of patients. To obtain a complete view of the genome‐wide 5hmC changes, 133 portal venous blood samples and their matched 64 peripheral blood (PB) samples, and 72 tumour and adjacent tissue (TAT) samples were selected for 5hmC‐Seal analysis (Figure 1A).

Before this study, we performed QC analysis for 5hmC‐Seal data in all samples and every sample, including the total reads, unique mapping ratio, and total peaks (Figure S1A–C). Meanwhile, we found that the patients with PC have more peaks enriched in the enhancers (Figure S1D). Next, we explored the correlation between 5hmC in portal venous blood and peripheral blood plasma cell‐free DNA (cfDNA), and 5hmC in tissue genomic DNA (gDNA). For the top‐ranked genes in terms of variability in cfDNA, there was a higher correlation between tumour gDNA and portal venous blood profiles (Figure S1F) than between tumour gDNA and peripheral blood (Figure S1E) (mean Pearson's *r* .68 vs. .25, Wilcoxon rank‐sum test *p* < .01), providing further evidence that 5hmC in portal venous blood cfDNA was relevant to the tumour origin. In addition, our results indicated that there were no significant differences in cfDNA and 5hmC contents in peripheral blood among the PC, SLM and MLM groups (Figure S2A and B). However, the cfDNA content in portal vein blood was significantly increased in SLM group (Figure S2C), and there was no significant change in 5hmC (Figure S2D). At the same time, we compared the changes of cfDNA and 5hmC content in portal vein blood and peripheral blood, and found no significant difference between the two groups (Figure S2E and F).

To determine the genome‐wide distribution of 5hmC, we generated metagene profiles of normalised 5hmC read counts. In portal venous blood plasma cfDNA, 5hmC enrichment was observed throughout the gene bodies with a significant decrease in the surrounding transcription starting sites (TSSs), and 5hmC densities in MLM were lower than those in PC and SLM throughout the gene bodies (Figure 1B). Hydroxymethylated regions (hMRs) were enriched within the gene bodies of each sample. Statistical analysis of the hMRs distribution across different genomic elements revealed that hMRs were mostly enriched in introns in all samples (Figure 1C). We then identified the 5hmC‐enriched peaks among the three groups and found that the patients with PC enriched more peaks than the MLM and SLM groups in different genomic characteristic regions, such as promoters, introns, and exons (Figure S1G). In tissue gDNA, we then generated metagene profiles of normalised 5hmC read counts in PC tumour tissue (PT), PC adjacent normal tissue (PN), SLM primary tumour tissue (SPT), SLM primary adjacent normal tissue (SPN), SLM metastatic tumour tissue (SMT), SLM metastatic adjacent normal tissue (SMN), MLM primary tumour tissue (MPT) and MLM primary adjacent normal tissue (MPN), and found that 5hmC densities in tumour tissue gDNA were lower than adjacent tissue gDNA throughout the gene bodies (Figure 1D). Similarly, we found that hMRs were mostly enriched in introns in all the samples (Figure 1E). Principal component analysis (PCA) based on top variance hMRs showed that the PT, SPT, and MPT groups could be separated from the PN group based on the 5hmC patterns (Figure S1H).

In summary, we established a genome‐wide map of the hydroxymethylome in portal venous blood cfDNA and tissue genomic DNA. Furthermore, our results suggest that the genome‐wide loss of the 5hmC landscape represented a new type of epigenetic alteration in primary tumours during the process of liver metastasis.

### 2.2 Identification of differentially 5hmC‐enriched regions of groups PC, MLM and SLM

**FIGURE 2:**
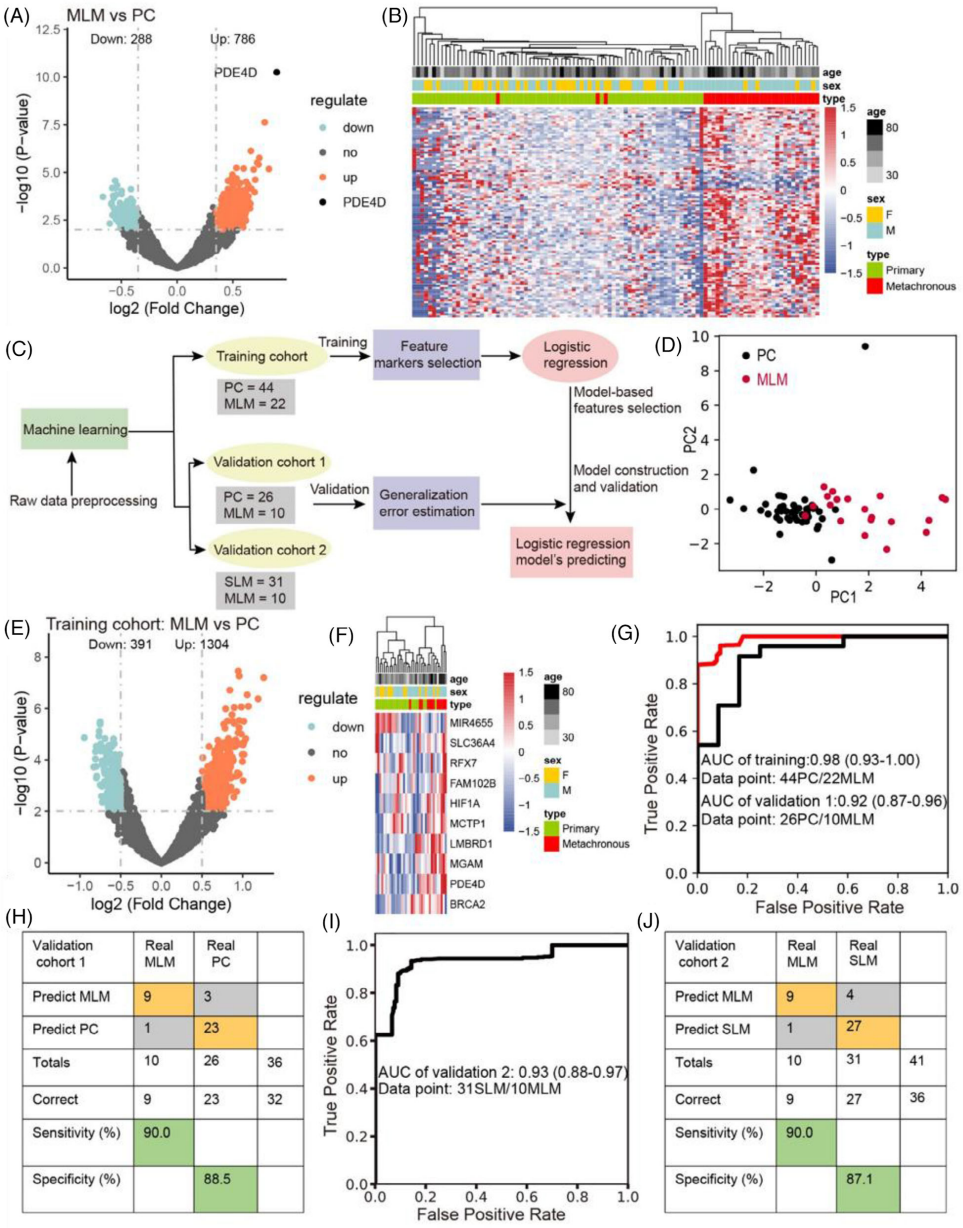
Prediction for MLM in the training and validation cohort. (A) Volcano plot of hMRs (MLM patients vs. PC patients). Significantly enriched regions on PDE4D gene in MLM patients were represented with black dots. Significance: (|log2FoldChange| ≥ .35 and *p*‐value < .01). (B) Heatmaps of 100 5hmC markers with MLM and PC patients; age and sex information are provided. Hierarchical clustering was performed across DhMRs and samples. (C) Workflow of machine learning. (D) PCA plot based on top variance hMRs of PC and MLM patients in plasma cfDNA. (E) Volcano plot. Significantly altered hMRs (abs (log2 Foldchange) ≥.5; *p*‐value < .01) are highlighted using the MLM group as the reference (*n* = 1695) in the training cohort. Black dots represent the hMRs that are not differentially expressed. (F) Heatmaps of 10 5hmC markers with PC and MLM patients in the validation cohort, sex and age information provided. (G) Receiver operating characteristic (ROC) curve of the classification model with 10 5hmC markers in the training cohort and validation cohort 1. The true positive rate (sensitivity) is plotted in function of the false positive rate (1 − specificity). (H) Confusion matrices built from the prediction model in the validation cohort 1. (I) Receiver operating characteristic (ROC) curve of the classification model with 10 5hmC markers in the validation cohort 2. The true positive rate (sensitivity) is plotted in function of the false positive rate (1 − specificity). (J) Confusion matrices built from the prediction model in the validation cohort 2.

We performed deep‐scale molecular analysis spanning 5hmC‐Seal data types on prospective CRC portal venous blood samples collected from 133 patients. First, we conducted differential analysis (DESeq2; *p* < .01, |log2FoldChange| ≥ .35) and observed 1074 differentially 5hmC‐enriched regions (DhMRs), including up regulated (*n* = 786) and down regulated (*n* = 288) regions in MLM compared to PC (Figure 2A). For instance, the hydroxymethylation level of PDE4D was the highest in MLM patients. According to recent studies, high hydroxymethylation in gene body is associated with up‐regulation of gene expression, which is easier to develop and apply than inhibition from the perspective of drug development.^25^, ^33^ Through Metascape enrichment analysis of up‐regulated differentially hydroxymethylated genes (DhMGs), we found that the top enriched GO biological pathways included signalling pathways such as inflammation and immunity, Rho GTPase, metabolism and protein phosphorylation (Figure S3A). Among these pathways, signalling by inflammation and immunity are relevant to tumour growth and apoptosis, suggesting that DhMGs might be involved in the immune system.^34^, ^35^, ^36^ Similarly, many studies have shown that protein phosphorylation is closely related to tumourigenesis, progression, and metastases.^37^, ^38^, ^39^, ^40^ Furthermore, the analysis of gene lists in the context of protein interactions can help illuminate biochemical complexes or signal transduction components that govern biological outputs. The GO functional interaction networks (Figure S3B) showed that these genes related with the protein phosphorylation, inflammation and immunity pathway, including phosphodiesterase 4D (PDE4D), CD44 molecule (CD44), cyclin dependent kinase 14 (CDK14) and C‐C motif chemokine ligand 28 (CCL28). Meanwhile, heatmap results, using default clustering methods, demonstrated that these 100 DhMGs could effectively distinguish patients with MLM from those with PC (Figure 2B).

In addition, we found 1098 DhMRs (DESeq2; *p* < .01, |log2FoldChange| ≥ .5) in SLM patients compared to PC patients (Figure S4A). Similarly, SLM‐related up regulation of DhMG was enriched in immunity and protein phosphorylation signalling pathways (Figure S4B). *PDE4D* and *CDK14* also related with these pathways (Figure S4C). Our results suggest that these DhMGs could closely associated with liver metastasis in colorectal cancer.

### 2.3 Screening for characteristic markers associated with metachronous liver metastasis in colorectal cancer

Colorectal cancer patients were randomly divided into a training cohort (22 with MLM and 44 with PC) and a validation cohort (10 with MLM and 26 with PC), and a 5hmC‐based logistic regression model was developed from the training cohort to predict MLM in the validation cohort (Figure 2C). PCA based on top variance hMRs showed that MLM samples could be separated from the PC samples based on the 5hmC patterns in the training cohort (Figure 2D). First, we conducted differential analysis (|log2FoldChange| ≥ .5, *p* < .01) and observed 1695 DhMRs, including up regulated (*n* = 1304) and down regulated (*n* = 391) regions, among the MLM patients compared to the PC patients (Figure 2E). Furthermore, using the recursive feature elimination algorithm based on the logistic regression CV estimator, we further reduced the number of 5hmC markers from 1695 to 10, thus achieving the optimum cross‐validation score. Finally, we found that the 10 5hmC markers (Table S2), selected by the LR model, could distinguish MLM patients from PC patients in both the training and validation cohorts (Figures 2F and S5A). Meanwhile, these 10 5hmC markers could effectively predict MLM and PC in the training cohort (AUC = .98) and validation cohorts 1 (AUC = .92) (Figure 2G), achieving 95.0% sensitivity and 100% specificity in the training cohort (Figure S5B), and 90.0% sensitivity and 88.5% specificity in the validation cohort 1 (Figure 2H). At the same time, ROC analysis showed that the clinical characteristics predicted MLM (CEA: AUC, .82; and 95% confidence intervals (CI), .55 to .69; CA199: AUC, .61; 95% CI, .52 to .67) (Figure S5C and D), but their predictive power are significantly lower than that of the 5hmC model. In addition, the prediction model based on these 10 5hmC markers can effectively distinguish SLM and MLM in validation cohort 2, with an AUC value of .93 (Figure 2I), sensitivity of 90.0%, and specificity of 87.1% (Figure 2J). This suggests that the underlying mechanisms of synchronous and metachronous liver metastases are different. It also indicates that the 5hmC prediction model can accurately distinguish metachronous liver metastasis, providing an important reference for early clinical intervention and treatment. Also, the AUC value of the 5hmC marker in peripheral blood to distinguish PC from MLM was .859, which was less accurate than the prediction model of portal vein blood (Figure S2G). Finally, we calculated the individual AUC for each of the 10 5hmC markers in the training cohort and validation cohort 1 (Figure S5E–N). Among these, *PDE4D* yielded optimal predictive performance, with an AUC of .83 in the validation cohort. These results suggest that changes in the hydroxylmethylation levels of selected genes may predict the occurrence of metachronous liver metastasis in patients with CRC.

### 2.4 Potential therapeutic target for the prevention of metachronous liver metastasis in colorectal cancer

**FIGURE 3:**
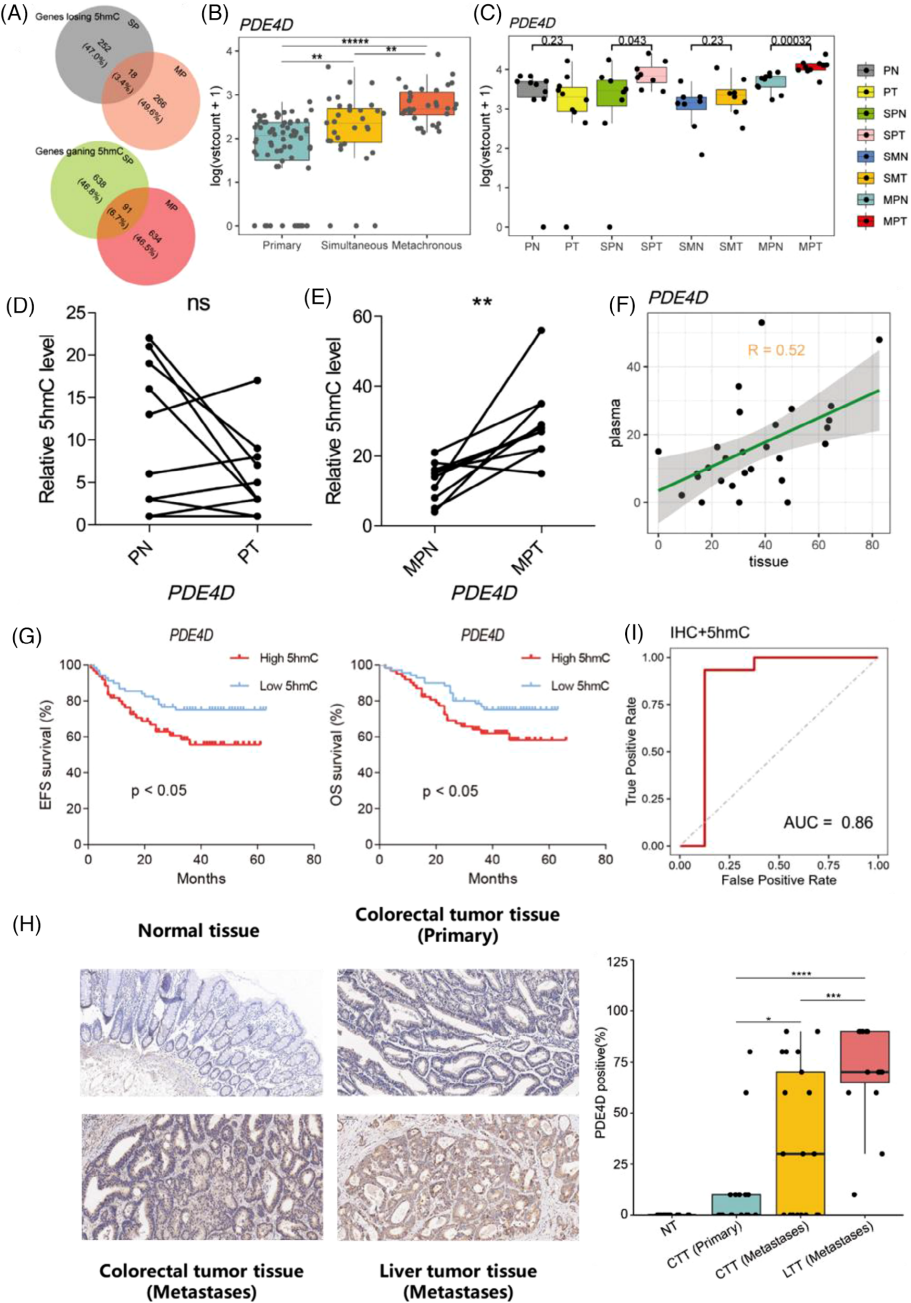
Functional relevance of the 5hmC‐based predict marker for in colorectal cancer. (A) Venn diagram indicating overlap and specificity of increase or decrease in PC patients and MLM patients from 5hmC‐Seal. (B) Boxplots of PDE4D 5hmC FPKM (Fragments per kilobase of gene per million mapped reads) in PC, SLM and MLM groups. Welch *t*‐test was used. (C) Boxplots of PDE4D 5hmC FPKM (Fragments per kilobase of gene per million mapped reads) in gDNA of tissue samples. Welch *t*‐test was used. (D) Quantitative comparison of PDE4D 5hmC levels in PN and PT. The two dots connected are samples from the same patient. (E) Quantitative comparison of PDE4D 5hmC levels in MPN and MPT. (F) Correlation plots of the source of cfDNA with the source of tissue gDNA in PDE4D. (G) The overall survival time (OS, months) and event free survival (EFS, months) of patients with different 5hmC level of PDE4D in 133 portal vein blood samples. (H) Immunohistochemical detection of PDE4D expression in normal tissue, colorectal tumour tissue (primary), colorectal tumour tissue (metastases) and liver tumour tissue (*n* = 15). Scale bar, 100 mm. (I) ROC curve of the classification model of liver metastases with 5hmC level and protein expression level of PDE4D. *p < .05; **p < .01; ***p < .001. CTT: colorectal tumour tissue, LTT: liver tumour tissue.

To localise the gene‐specific 5hmC changes between the MLM and SLM, DhMGs were identified for each pairwise comparison. Through analysis, 1009 DhMGs (DhMGs‐gain, 725; DhMGs‐loss, 284) and 989 DhMGs (DhMGs‐gain, 729; DhMGs‐loss, 260) were identified in the MLM and SLM, respectively. Venn diagram results showed 91 DhMGs‐gain and 18 DhMGs‐loss identified in both groups (Figure 3A). Intriguingly, the hydroxymethylation levels of PDE4D were highest in the MLM group and lowest in the PC group (Figure 3B).

Phosphodiesterase‐4 (PDE4) is a member of the phosphodiesterase family and is involved in important physiological regulatory functions through the hydrolysis of cAMP.^41^ There are four subtypes of *PDE4*: *PDE4A*, *PDE4B*, *PDE4C*, and *PDE4D*. *PDE4A*, *PDE4B*, and *PDE4D* are widely distributed in the human brain, particularly in the hippocampus, olfactory bulb, and cerebellum.^42^, ^43^ Additionally, these three isoforms are detected at high levels in many inflammatory and immune cells, such as neutrophils, eosinophils, mast cells, basophils, lymphocytes, dendritic cells, macrophages, monocytes, and smooth muscle cells.^44^ Therefore, It may serve as a potential drug target for the treatment of various diseases.

To investigate the changes in hydroxymethylation of *PDE4D* in tissue samples, we compared 28 pairs of 5hmC‐Seal data from tissue genomic DNA (gDNA). Similarly, the hydroxymethylation levels of *PDE4D* increased in the SPT and MPT groups, providing further evidence for the association between *PDE4D* and liver metastasis of CRC (Figure 3C). Meanwhile, the hydroxymethylation level of *PDE4D* in the tumour gDNA of the same MLM patients was higher than that in the adjacent tissue (Figure 3D–E). Furthermore, *PDE4D* hydroxylmethylation sites in cfDNA were strongly correlated with tissue DNA (*R* = .52) (Figure 3F). Moreover, from the survival analysis results in 133 portal vein blood samples 5hmC‐Seal dataset, we found that the overall survival time (OS, months) and event‐free survival (EFS, months) of patients with high 5hmC levels of *PDE4D* were significantly lower than those of patients with low 5hmC levels (Figure 3G). We detected the protein levels of *PDE4D* in normal tissue (NT), colorectal tumour tissue (CTT), and liver tumour tissue (LTT). In line with the IHC results, *PDE4D* was significantly overexpressed in tumour tissues compared to normal tissues and exhibited the highest expression in metastatic lesions (Figure 3H). Patients with liver metastases showed a higher expression level of *PDE4D* in CTT than those without metastases. The RNA expression of *PDE4D* in tissue samples were also detected, which showed an increase in *PDE4D* RNA level in MLM patients (Figure S5O). Moreover, patients with higher hydroxymethylation and protein levels of *PDE4D* were prone to develop liver metastases, while 86% of patients with metastases could be diagnosed based on hydroxymethylation and protein levels of *PDE4D* (Figure 3I). We speculated that *PDE4D* plays a key role in the pathogenesis of colorectal cancer liver metastasis.

### 2.5 Interfering with the PDE4D function represses liver metastasis

**FIGURE 4:**
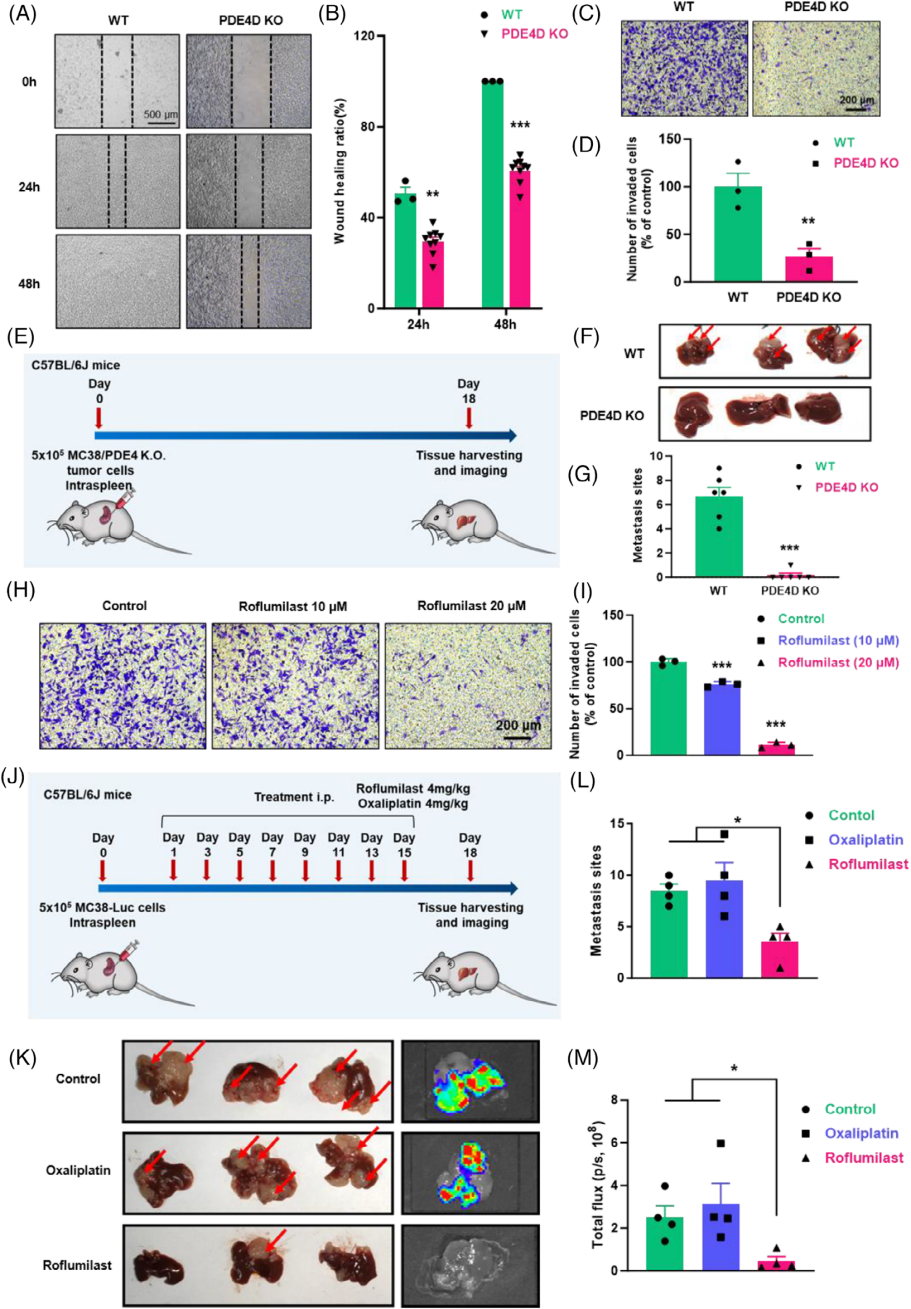
Interfering the function of PDE4D inhibits CRC liver metastasis. (A) Analysis of CRC cell migration of PDE4D KO cells by in vitro scratch assays. Images were acquired at 0, 24, and 48 h. Scale bar, 500 µm. (B) Quantitative analysis of scratch wound closure. (WT, *n* = 3; PDE4D KO, *n* = 9) (C) The migration of PDE4D KO cells was assessed using a transwell migration assays. Scale bar, 200 µm. (D) Quantitatively statistic of transwell assay (*n* = 3). (E) A flowchart depicting the in vivo experimental design and morphology. (F) Representative images of livers with experimental MC38 cell or PDE4D KO cell liver metastases. (G) Quantitative analysis of metastases sites of liver (*n* = 6). (H) Transwell migration images of MC38 cells treated with roflumilast. Scale bar, 200 µm (*n* = 3). (I) Statistical analysis results of the transwell assay (*n* = 3). (J) Schematic of spleen injection of MC38 cells and treatment timeline with roflumilast and oxaliplatin. (K) Representative images and fluorescence images of livers with experimental MC38 liver metastases. (L to M) Quantitative analysis of metastases sites (L) and fluorescence intensity (M) of liver (*n* = 4). Data are shown as means ± SEM. *p < .05; **p < .01; ***p < .001.

To further elucidate the function of PDE4D in CRC liver metastases, we used CRISPR/Cas9 to generate PDE4D knockout MC38 cells (PDE4D KO cells). As expected, the protein level of PDE4D in PDE4D KO cells was significantly decreased compared to that in WT MC38 cells, while the statistical data indicated the same result (Figure S6A). The proliferation of PDE4D KO cells was the same as that of WT MC38 cells (Figure S6B). Simultaneously, the results of wound‐healing scratch assays showed that the migration of PDE4D KO cells was significantly inhibited compared to that of WT MC38 cells (Figure 4A and B). Transwell assays also showed that the metastatic ability of PDE4D KO cells was inhibited (Figure 4C and D). Additionally, we used the shRNA approach to establish PDE4D knockdown SW480 cell line. The proliferation of shPDE4D cells did not change compared with that of SW480 cells (Figure S6C). In vitro transwell assay indicated that cell migration was significantly inhibited after PDE4D knockdown (Figure S6D). We further investigated whether PDE4D knockout could inhibit tumour metastases in vivo. WT MC38 cells and PDE4D KO cells were intraspleen injected into mice to establish liver metastasis model, reflecting MLM in clinical trials (Figure 4E). After 18 days, the mice were sacrificed, and the livers harvested. Images of the liver indicated that mice injected with PDE4D KO cells had remarkably fewer liver metastases than those injected with WT MC38 cells (Figure 4F and G).

Over the past few decades, numerous attempts to develop PDE4 inhibitors for treating central nervous system (CNS) diseases or other diseases have been made. Among these, roflumilast (Rof) was widely used for the clinical treatment of chronic obstructive pulmonary disease (COPD). In line with these findings, we selected roflumilast as a potential drug to prevent CRC liver metastases. The results of the CCK8 assays showed less toxicity in CRC cells (Figure S6E). However, scratch wounding assays showed that roflumilast treatment for 24 h and 48 h significantly inhibited the migration of MC38 cells (Figure S6F). Transwell assay images and statistical analysis results further showed that roflumilast treatment significantly inhibited wound closure in MC38 cells (Figure 4H and I). A similar trend was observed when LoVo and HCT116 cells were recipient cells (Figure S6G and H). To validate the potential contribution to tumour metastasis in vivo, roflumilast was intraperitoneally injected into mice after establishing a liver metastasis model with luciferase‐encoding MC38 cells (MC38‐Luc) (Figure 4J). After two weeks of treatment, the livers of mice, as well as bioluminescent images, suggested that fewer metastatic lesions were formed by roflumilast treatment (Figure 4K). Quantitative statistics of metastatic sites (Figure 4L) and total flux (Figure 4M) also showed that roflumilast could inhibit liver metastasis of CRC cells.

### 2.6 The mechanism of PDE4D in liver metastasis of CRC

**FIGURE 5:**
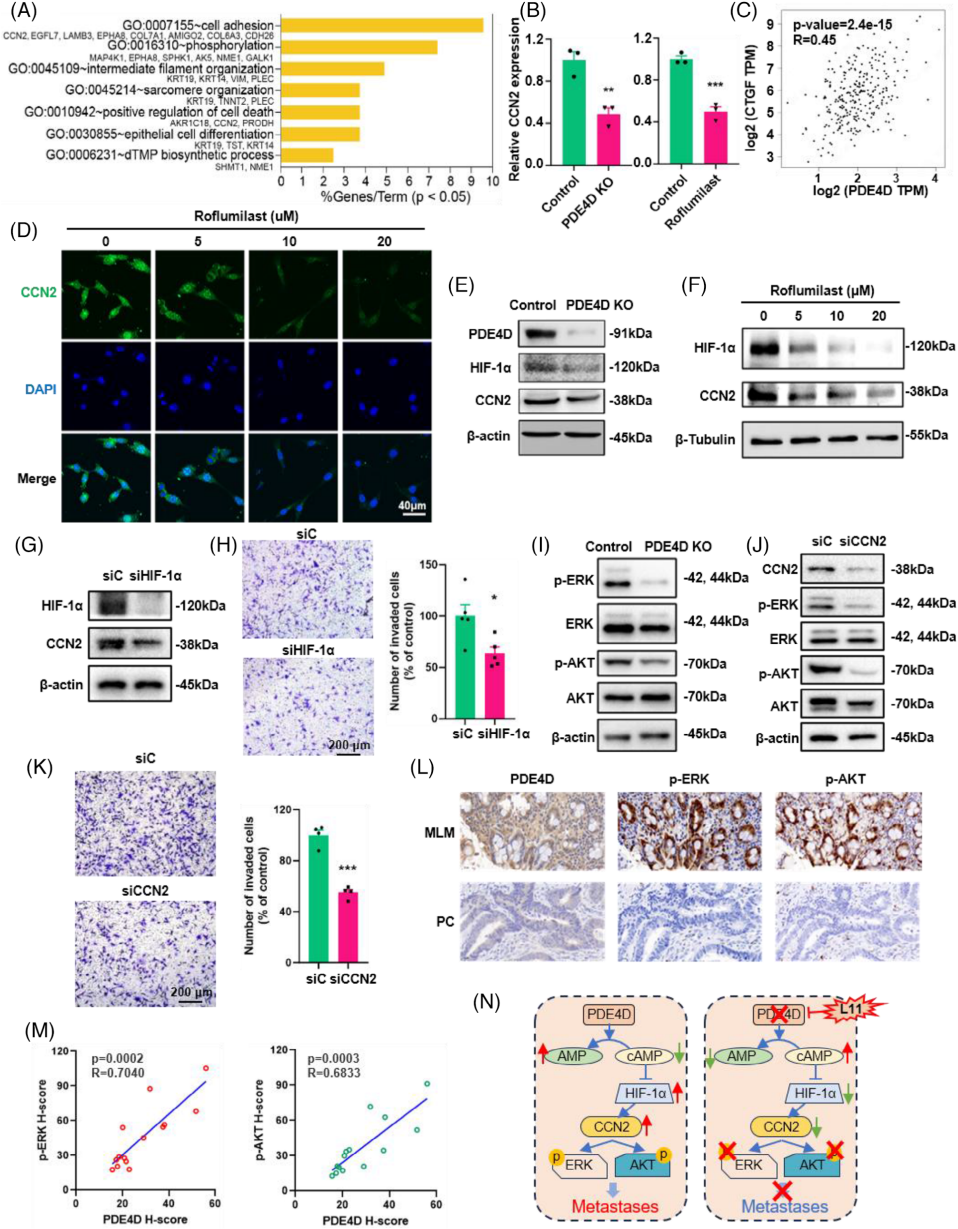
Molecular mechanism of PDE4D for CRC liver metastasis. (A) GO enrichment analysis and function exploration of downregulated genes. (B) Expression of CCN2 in PDE4D KO cells and roflumilast treated cells (*n* = 3). (C) Correlation plots of the mRNA expression of PDE4D with the mRNA expressions of CCN2 in CRC sequencing data from TCGA dataset. (D) Immunofluorescence images for CCN2 in MC38 cells treated with roflumilast for 48 h. (E) Analysis of HIF‐1α and CCN2 expression in PDE4D KO cells by western blot. (F) Expression of HIF‐1α and CCN2 in roflumilast treated cells detected by western blot. (G) Western blot analysis of HIF‐1α and CCN2 expression in MC38 cells after HIF1A knockdown. (H) The migration of MC38 cells after HIF1A knockdown (*n* = 5). (I) Western blot for p‐AKT and p‐ERK in PDE4D KO MC38 cells. (J) Western blot for p‐AKT and p‐ERK in CCN2 KD MC38 cells. (K) The migration of MC38 cells after CCN2 knockdown (*n* = 4). (L) Immunohistochemical detection of p‐ERK and p‐AKT expression in tissue samples of MLM patients and PC patients (*n* = 14). (M) The correlation analysis for PDE4D and p‐ERK or p‐AKT in tissue samples of MLM patients and PC patients (*n* = 14). (N) Model for mechanism of the treatment with roflumilast inhibiting metastases in CRC. Data are shown as means ± SEM. *p < .05; **p < .01; ***p < .001.

To reveal the mechanism of PDE4D in liver metastasis, we performed RNA sequencing analysis to measure differential gene expression in PDE4D KO and MC38 cells incubated with roflumilast. The results indicated that PDE4D KO cells could be significantly distinguished by principal component (PCA) analysis (Figure S7A). Similar results were observed in the RNA sequencing data between MC38 cells and cells treated with roflumilast (Figure S7B). Differential analysis revealed 2541 differential gene expressions in PDE4D KO cells, including upregulated (*n* = 1294) and downregulated (*n* = 1247) genes (Figure S7C). In addition, 130 upregulated genes and 190 downregulated were observed in roflumilast‐treated cells (Figure S7D). The heatmap results further demonstrated that both PDE4D KO cells and cells treated with roflumilast could be effectively separated (Figure S7E). Based on pairwise comparison analysis, 137 upregulated and 85 downregulated genes were identified in both groups (Figure S7F). Intriguingly, GO pathway analysis revealed that the downregulation of genetic variations aggregated in cell adhesion, phosphorylation, intermediate filament organisation, positive regulation of cell death, and many other complex biological processes (Figure 5A). Among these, adhesion is essential for cell migration. All eight genes (*CCN2*, *LAMB3*, *EGFL7*, *EPHA8*, *COL7A1*, *AMIGO2*, *COL6A3*, and *CDH26*) in the cell adhesion pathway had relatively low expression in both PDE4D KO cells and cells treated with roflumilast (Figures 5B and S7G). In addition, we analysed the association between genes based on the sequencing data of CRC patients in TCGA database, and found that the expression of several genes was positively correlated with *PDE4D*, including *CCN2* and *COL6A3* (Figures 5C and S7H). In view of the above results, we focused on *CCN2* in further research.

To confirm that PDE4D promotes colorectal cancer metastasis by upregulating CCN2, we performed immunofluorescence (IF) in PDE4D inhibitor (roflumilast) treated MC38 cells. Fluorescent images revealed that the protein level of CCN2 decreased significantly after treatment with roflumilast (Figure 5D). While CCN2 showed a significant decrease in both mRNA and protein levels, we focused on the transcription factors of CCN2, including HIF1A, EGR1, YAP1, TEAD1, CREB1, SRF, SMAD1, and RELA. Correlation analysis showed that the mRNA level of PDE4D was highly correlated with that of HIF1A (*R* = .66) (Figure S8A). According to previous studies, HIF‐1α directly binds to the HRE regions in the transcription start site of CCN2 and controls CCN2 expression.^45^ In our study, the protein levels of HIF‐1α and CCN2 were significantly decreased in PDE4D KO cells (Figure 5E). In addition, PDE4D inhibitor (roflumilast) inhibited HIF‐1α and CCN2 expression levels in MC38 cells after incubating for 24 h (Figure 5F). Similar results were observed in HCT116 cells (Figure S8B). To demonstrate that HIF‐1α positively regulates CCN2, we knockdown HIF‐1α in MC38 cells using short hairpin RNA. A significant decrease in the mRNA and protein levels of CCN2 were observed after HIF‐1α knockdown (Figure 5G and H) and HIF‐1α inhibitors (KC7F2 and 2‐MEOE2) treatment (Figure S8C). Additionally, the metastasis efficiency of MC38 cells was significantly reduced (Figure 5H). To further prove the PDE4D‐HIF1a‐CNN2 pathway, we combined roflumilast with siRNA. Results showed that roflumilast could not inhibit tumour metastases combined with siCCN2 (Figure S8D). Same result was observed when MC38 cells treated with roflumilast and siHIF1A (Figure S8E). These results indicated that PDE4D acts exclusively via the PDE4D‐HIF11a‐CNN2 pathway.

Previous studies have shown that CCN2 is associated with the phosphorylation of AKT and ERK.^46^, ^47^ To determine the feasibility of the mechanism in our model, we examined the phosphorylation levels of ERK and AKT in PDE4D knockout cells and found that p‐ERK and p‐AKT were significantly reduced following PDE4D knockout and inhibition (Figures 5I and S9A and B). We also detected a decrease in CCN2, p‐AKT, and p‐ERK in the SW480 PDE4D knockdown cell lines (Figure S9C). In addition, we discovered that knockdown CCN2 can lead to a reduction in the levels of p‐ERK and p‐AKT and the metastases efficiency of MC38 cells (Figure 5J and K). We also detected the protein levels of PDE4D and phosphorylation levels of ERK and AKT in CRC patients, found the levels of PDE4D, p‐ERK and p‐AKT was higher in MLM samples than that in PC samples, the p‐ERK and p‐AKT exhibited significant correlations with the expression of PDE4D (Figure 5L and M). Furthermore, the ERK inhibitor (PD98095) and AKT inhibitor (MK2206) could significantly inhibit MC38 metastases in transwell analysis (Figure S9D–G). The above results indicate that PDE4D affects the phosphorylation levels of ERK and AKT through HIF1a‐CCN2 pathway, further influencing the capability of metastases in CRC.

PDE4 plays an important role in the hydrolysis of cAMP, its inhibition of PDE4 leads to an increase in intracellular cAMP levels. Therefore, we tested whether forskolin (FSK), a cAMP activator, could affect downstream molecules. Treatment with FSK significantly inhibited the mRNA and protein levels of CCN2, p‐AKT, and p‐ERK (Figure S9H). According to the transwell assay results, the effects of FSK were quantitatively similar to those of roflumilast (Figure S9I). Generally, PDE4D was important in CRC metastases, the inhibition of PDE4D led to an increase in cAMP, further inhibiting the protein expression of HIF‐1α, as well as the mRNA and protein levels of CCN2 and the phosphorylation of AKT and ERK, and consequently inhibiting metastasis (Figure 5N).

### 2.7 Synthesis and biological activity of novel PDE4 inhibitors

**FIGURE 6:**
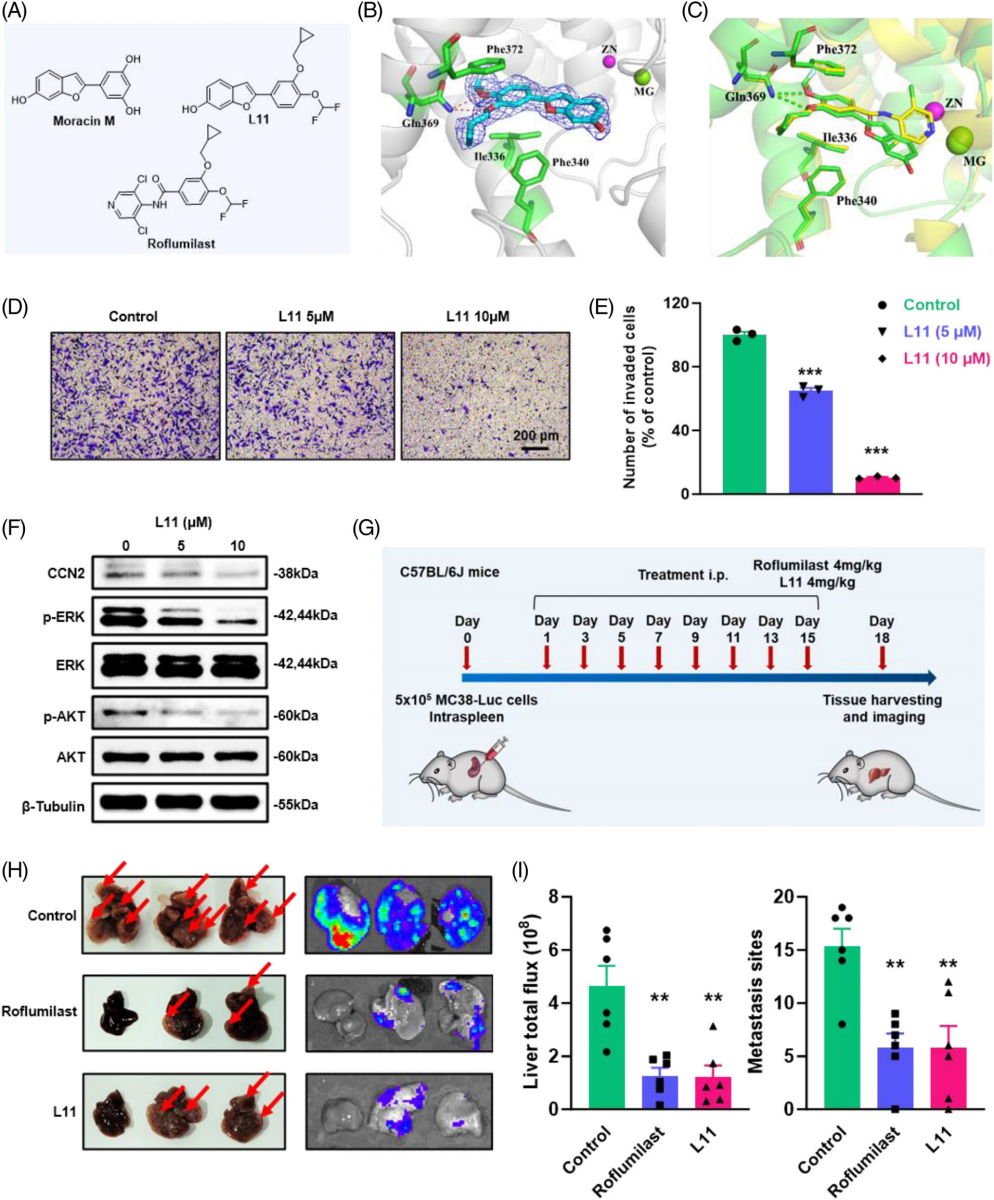
Compound L11 inhibit CRC liver metastases in vitro and in vivo. (A) Molecular structure of compound **L11**. (B) Cartoon representation of the crystal structure of PDE4D protein in complex with compound **L11** is represented by magenta sticks. (C) Comparison of the binding modes of roflumilast and compound **L11** in PDE4D protein. (D) Transwell migration assays of MC38 cells treated with compound **L11**. Scale bar, 200 µm. (E) Representative images of transwell assay (*n* = 3). (F) Western blot for CCN2, phospho‐AKT, total AKT, phospho‐ERK and total ERK in MC38 cells treated with compound **L11**. (G) Schematic of spleen injection of MC38 cells and treatment timeline with roflumilast and compound **L11**. (H) Representative and bioluminescence images of livers with experimental MC38 liver metastases. (I) Quantitative analysis of liver metastases sites and fluorescence intensity analysis of liver after two weeks treatment (*n* = 6). Data are shown as means ± SEM. *p < .05; **p < .01; ***p < .001.

Despite their therapeutic effect, the clinical application of most PDE4 inhibitors, including roflumilast, is limited by their unexpected and severe side effects such as nausea, vomiting, and diarrhoea; however, the exact mechanisms underlying these adverse effects are unclear. To develop more effective PDE4 inhibitors to resolve existing challenges, it is necessary to develop novel scaffolds for drug discovery. Natural products have been the inspiration sources of drug discovery for centuries because of their benefits in various disease therapies. *Cortex Mori*, the root bark of *Morus* plants, is a traditional Chinese medicine used to treat hyperactive cough, oedema, puffiness oliguria, and face puffiness(48). *Cortex Mori* provides a wide range of compounds with different structures, including 2‐arylbenzofurans, flavonoids, stilbenoids, and their pharmacological effects, including antioxidative, antitumour, antimicrobic, and anti‐inflammatory effects.^48^ Moracin M is a 2‐arylbenzofurans component found in *Cortex Mori*. It has weak inhibitory effects on PDE4B2 (IC_50 _= 4.5 µM) and PDE4D2 (IC_50 _= 2.9 µM)(49). Moreover, molecular docking and molecular dynamics simulations indicated that it could bind well to the active pocket of PDE4,^49^, ^50^ which encouraged us to develop novel PDE4 inhibitors. Based on these previous studies, we designed and synthesised a new series of 2‐arylbenzofurans as potent PDE4 inhibitors (Scheme S2 and S3, Data S1 and Data S2). We then determined the potency of these compounds against PDE4. In total, 13 derivatives of 2‐arylbenzofurans were discovered, resulting in 4 compounds with IC_50_ < 200 nM. The most potent inhibitor, **L11**, exhibited a potent affinity of 54 nM, which was better than that of other compounds (Figure 6A and Table S3). Based on these results, we selected compound **L11** for further research.

The selectivity profile of compound **L11** across other PDE families was examined (Table S4). Inhibition of PDE1B, PDE3A, and PDE9A2 was very weak (IC_50_ > 10 000 nmol/L). Its inhibitory activity against PDE2A, PDE5A1, PDE7A1, PDE8A1, PDE10A, and PDE11A was 90‐, 56‐, 42‐, 67‐, 116‐, and 74‐fold higher than that against PDE4D2. These results revealed that compound **L11** exhibits remarkable selectivity for PDE4D.

Given the potent inhibition of **L11**, the cocrystal structure of PDE4 with bound **L11** was obtained at 2.1 Å resolution. The liganded PDE4 crystals had the space group P2_1_2_1_2_1_ with unit cell dimensions of *a* = 58.36, *b* = 80.43 and *c* = 164.58 Å, and the structure was refined to *R*/*R*_free_ = .227/.278 (Table S5). As shown in Figure 6B, the 2Fo–Fc electron density unambiguously revealed the binding of **L11** to the PDE4 pocket. The scaffold of **L11** formed two H‐bonds with the conserved Gln369 and π−π stacking interactions with a hydrophobic clamp consisting of Phe372 and Phe340/Ile336. In addition, the 6‐hydroxybenzofuran fragment of **L11** formed an H‐bond with residue Asn209, mediated by a water molecule, which differs from the binding pattern of roflumilast. Further structural superposition of the crystal structures of PDE4‐Roflumilast and PDE4‐**L11** also revealed that **L11** has a slightly different binding pattern (Figure 6B and C).

To examine the safety of the newly discovered PDE4 inhibitor L11, we evaluated the emetic activity of compound L11 in Beagle dogs at an oral dose of 10 mg/kg (Table S6). **L11** did not induce emetic effects in dogs at this dose, indicating that L11 is well‐tolerated in dogs. To examine the long‐term safety of the newly discovered PDE4 inhibitor L11, the rats were randomly divided into three groups with each group comprising 10 male and 10 female rats: control group, compound L11 (30 mg/kg), and compound L11 (100 mg/kg). Then compound L11 (30 mg/kg or 100 mg/kg) were administered via intraperitoneal injection for 2 weeks, respectively. As a result, no abnormal changes were observed in food intake, weight, haematology indicators and serum test indicators (Tables S7–13). Our results demonstrated that the compound L11 did not cause any adverse effects on body weight or other signs of overt toxicity at daily doses of up to 100 mg/kg for 2 weeks.

### 2.8 Compound L11 suppresses liver metastases in vitro and in vivo

Based on these results, we further characterised the effect of compound **L11** on CRC liver metastases in vitro and in vivo. The results of the CCK8 assay showed that the IC_50_ of compound **L11** was approximately 20 µM (Figure S12A). Compared with result of roflumilast (Figure S6E), compound **L11** could significantly inhibit the growth of MC38 cells. In addition, compound **L11** treatment markedly reduced the migration of CRC cells in vitro, according to wound closure assays (Figure S12B), and transwell assays (Figure 6D and E). Based on the statistical data, compound **L11** showed more optimised inhibitory function than roflumilast. Meanwhile, according to the western blot results, compound **L11** significantly repressed the expression of CCN2 as well as the phosphorylation of AKT and ERK, which was similar to the mechanism of roflumilast (Figure 6F).

Based on these in vitro findings, compound **L11** was further tested in a liver metastasis allograft model generated from MC38 cells (Figure 6G). The bioluminescence of mice treated with roflumilast and **L11** taken on day 18 showed a significant decrease in the liver (Figure S12C and D). The mice were sacrificed and organs were harvested after treatment. The images of the liver, as well as bioluminescence images, suggested that the administration of compound **L11** could significantly inhibit tumour metastases in the liver (Figure 6H). The graphs of metastasis sites, liver total flux and liver weight also indicated that compound **L11** and roflumilast had similar effects on blocking tumour metastases (Figures 6I and S12E). H&E staining images and statistical results of metastatic areas in the liver confirmed that compound **L11** treatment significantly suppressed liver metastases (Figure S12F and G). Moreover, the safety of compound **L11** was observed in vivo according to the body weight curve and H&E staining results for the heart, lung and kidney (Figure S12H and I). These results indicated **L11** is a potential drug for the treatment of asynchronous liver metastases in clinical trials.

## 3 DISCUSSION

Although advanced treatments have substantially extended the survival of patients with colorectal cancer, recurrence of MLM following surgery continues to be the primary cause of mortality in patients.^51^ Liquid biopsy is minimally invasive and easier to perform throughout the course of disease progression than other tumour‐based approaches.^52^ Advances in liquid biopsy provides hope for the diagnosis, progression, and prognosis of tumours.^53^, ^54^ As a novel epigenetic biomarker, 5hmC plays a critical role in gene expression regulation and is involved in various biological processes, including solid and haematological tumours.^33^, ^55^, ^56^ In addition, tumour cells release large quantities of cfDNA, which has been used in the appraisal of numerous oncogenes and other diseases.^57^, ^58^ To identify reliable biomarkers and therapeutic targets for MLM, we utilised the 5hmC‐Seal sequencing method to detect cfDNA 5hmC features of CRC patients.

Typically, in previous studies, most samples for liquid biopsy were peripheral blood, which was relatively easy to obtain. However, most of the blood in the digestive tract flows back into the liver through the portal vein. Therefore, the portal vein is the main site for primary cancer to spread to the liver. Based on previous research, metastatic CTC were identified in portal vein samples from all patients, as opposed to only 22.2% of matched peripheral blood samples, indicating a close relationship between the portal vein and tumour metastases.^59^ Higher positive rates of CTC were observed in the first drainage vein blood (FDVB) than in the peripheral blood. Moreover, CTC content is related to liver metastases, T stage, and CA19‐9 levels.^60^

The mechanism of CRC metastasis to the liver has been described in detail, with tumour cells entering the liver either via the portal vein or the hepatic artery.^61^, ^62^ To provide more granular insight into liver metastases, we collected portal venous blood from CRC patients with corresponding peripheral blood samples and tissue samples to map the complete view of genome‐wide 5hmC changes (Figure 1 and Table S1). By comparing the genome‐wide 5hmC profiles of peripheral blood cfDNA, portal venous blood cfDNA, and tissue samples, we found portal venous blood to be more highly correlated with tumour gDNA profiles than peripheral blood. Our results showed that MLM patients can be distinguished from SLM and PC patients by 5hmC features derived from portal venous blood and tissue. Notably, a logistic regression model based on DhMRs of portal venous blood demonstrated that 10 5hmC features could predict MLM with high sensitivity and specificity in both the training and validation cohorts (Figure 2).

However, the survival rate of patients with MLM is low. If the risk of MLM can be predicted, early intervention may significantly improve patient prognosis. However, there is still no effective method for alerting MLM patients, and MLM patients still use the same treatment as PC patients before occurrence.^63^, ^64^, ^65^ Our study is the first to offer a novel approach for pre‐warning of the occurrence of MLM based on 5hmC features derived from portal venous blood. This 5hmC approach could quickly and accurately screen patients at high risk of metastasis after surgery and serve as a convenient tool even for large populations. Compared with other prediction methods, such as CT radionics models, this 5hmC approach has a higher accuracy.^66^, ^67^

In CRC liver metastasis, mutations in KRAS, TP53, APC, PIK3CA, NRAS, BRAF, and SMAD4, accompanied by genomic and epigenetic instability, initiate CRC metastases.^68^, ^69^ Moreover, inflammatory cytokines secreted by tumour cells could can also induce tumour cell growth, invasion, and metastases through different mechanisms, including epithelial‐mesenchymal transition (EMT).^70^ Pathways related to immunity and inflammation were enriched in the 5hmC‐Seal data analysis. However, although clinical therapies for these aspects can control metastatic tumours, recurrence continues to pose a major challenge for successful treatment.^69^

To prevent MLM and prolong survival, our study identified PDE4D as a novel diagnostic and therapeutic target for CRC liver metastases and developed therapeutic strategies based on PDE4 inhibitors. We first reported PDE4D as one of the most important factors of CRC liver metastases. Interestingly, PDE4D is not a genetic dependent target with liver specificity according to DepMap (depmap.org/portal/). Our study revealed that CRC liver metastases is positively correlated with the expression of PDE4D, but not the mutations in PDE4D. These findings may provide a novel way for liver metastases research and treatment.

Previous studies have documented that cAMP signalling pathway can regulate numerous biological processes including migration, differentiation, proliferation, apoptosis and metastases inhibition.^71^, ^72^, ^73^, ^74^, ^75^ Our screening results are consistent with IHC detection, indicating that PDE4D is among the list of genes related to MLM (Figure 3). PDE4D is overexpressed in tumour tissues of patients with MLM but not in those of patients with PC. Notably, all patients with high 5hmC and IHC levels of PDE4D eventually developed liver metastasis. Therefore, we hypothesised that PDE4D plays a crucial role in MLM. Subsequently, we demonstrated that inhibition of PDE4D could repress CRC cell migration in vitro and liver metastasis in vivo via a gene knockout strategy. We found that roflumilast, a PDE4 inhibitor approved for chronic obstructive pulmonary disease (COPD), also inhibited metastases in vitro and in vivo (Figure 4). Since roflumilast is approved for clinical use, adding this drug to the treatment regimen for colon cancer may result in patient benefits.

Besides, our study is the first to observe a correlation between PDE4D and CCN2 in CRC metastases. The novel PDE4D‐CCN2 pathway we found also heralds a novel intervention in clinical treatment of liver metastasis (Figure 5). According to our RNA sequencing analysis, we found that RNA level of CCN2 was significantly reduced after PDE4D knockout or roflumilast incubation, consistent with the protein expression level. Abnormal CCN2 expression is associated with the progression of multiple cancers, including breast, prostate and pancreatic cancers.^76^, ^77^, ^78^ The mRNA and protein levels of CCN2, a well‐known transcriptional target of TGF‐β, are associated with CRC prognosis.^79^ CCN2 levels were associated with a decreased risk of peritoneal metastases and significantly higher disease‐free and overall survival.^80^ CCN2 levels are higher in advanced CRC stages.^81^ Notably, roflumilast inhibited the PDE4D/ CCN2 axis and further degraded the phosphorylation of AKT and ERK, which have been reported to be associated with metastases of numerous tumours.^82^, ^83^, ^84^, ^85^

Although roflumilast has achieved significant efficacy in clinical COPD treatment,^86^, ^87^ unexpected severe side effects of PDE4 inhibitors such as emesis, diarrhoea, nausea, and psychiatric adverse events cannot be disregarded.^88^ To develop a more efficient PDE4 inhibitor, we synthesised several novel compounds based on 2‐arylbenzofurans and verified them in subsequent studies. Among these new compounds, compound **L11** exhibited a potent affinity of 54 nM (Table S4), which was more optimised than that of the other compounds (Figure 6). Moreover, compound **L11** significantly suppressed cell migration in vitro and liver metastasis in vivo. Further research indicated that compound **L11** provided the same mechanism as roflumilast, inhibiting the expression of CCN2 as well as the phosphorylation of AKT and ERK. We also found that compound **L11** could not induce weight loss or any major organ damage in mice.

This study has several limitations that should be addressed in future research. Although key clinical variables, including gender and age, were controlled for in our study, the impact of other potential confounding factors should be explored and confirmed in future investigations. In addition, the number of patients analysed in our study was limited, and more independent participants are required to validate the marker panel. In addition to retrospective studies, prospective studies will be necessary to evaluate the predictive value in future stages, which could help to verify and establish the ultimate clinical utility of this method.

In conclusion, we developed and validated a novel early‐stage diagnosis for high‐risk MLM patients based on 5hmC and portal venous blood. We also identified PDE4D as a potential therapeutic target for the prevention of CRC metastasis. In addition, the novel compound **L11** discovered in our study could efficiently prevent CRC metastasis in vitro and in vivo and exhibited remarkable drug tolerance and less drug toxicity in vivo. Our findings provide promising strategies that could be used to predict the occurrence and evaluate the prognosis. Our companion diagnostics strategy of PDE4D may provide a novel approach to identify the most at‐risk patients and provide precision treatment. We expect that our approach and inhibitors such as roflumilast will benefit patients with CRC in clinical treatment in the future.

## 4 CONCLUSION

Our investigation presents a novel epigenetic liquid biopsy strategy using portal venous blood to predict metachronous liver metastases (MLM) in colorectal cancer (CRC) patients. By analysing 5‐hydroxymethylcytosine (5hmC) features in portal venous blood, our study found that portal venous blood was more relevant to tumour genomic DNA compared to peripheral blood, indicating its potential for early detection of MLM. The study identified 10 epigenetic alterations, particularly phosphodiesterase 4D (PDE4D), as significant biomarkers for distinguishing MLM patients from those without metastases. PDE4D was found to be highly increased in MLM patients and correlated with poor survival, suggesting it as a key metastasis‐driven target for drug development. Interfering with PDE4D function significantly inhibited liver metastases, and roflumilast, a PDE4 inhibitor approved for the treatment of chronic obstructive pulmonary disease, was also effective. Further research indicates that blocking the function of PDE4D can inhibit CRC metastases through the HIF‐1α‐CCN2‐AKT/ERK pathway. To develop a PDE4 inhibitor with fewer side effects, new compounds based on 2‐arylbenzofurans were designed and found to have potent affinity for PDE4D and significant suppression of liver metastases. Overall, this study provides a promising strategy for predicting MLM in CRC patients and discovers roflumilast as a potential repurposed drug for inhibiting liver metastasis. The findings have the potential to benefit CRC patients by improving early detection, therapeutic targeting, and survival outcomes.

## 5 MATERIALS AND METHODS

### 5.1 Study design

The aim of this study was to investigate epigenetic alterations between patients with and without liver metastases and find potential target against colorectal cancer. Epigenetic sequencing data were generated from peripheral blood, portal venous blood and tumour tissues by 5hmC‐Seal (Figure 1A). Analysis methods, predictive models and potential targets were developed using a combination of these datasets. Based on preliminary results, metastases models of colorectal cancer were established for verification of target and screening of antimetastases drugs in vitro and in vivo.

### 5.2 Materials

All cancer cell lines (MC38, SW480, LoVo, HCT116) were purchased from ATCC. The cell lines were maintained in RPMI 1640 medium supplemented with 10% fetal bovine serum (FBS) (Gibco), 100 U/mL penicillin, and 100 µg/mL streptomycin (both Gibco) in a 5% CO₂ incubator. C57BL/6 (for experimental metastases model) were purchased from Zhejiang Academy of Medical Sciences. Female mice aged 6–8 weeks were used in the study. All animal experiments were conducted in accordance with the guidelines approved by Peking University Third Hospital (No. A2023016).

### 5.3 Data and sample source

All enrolled patients received standard treatment: patients with middle‐to‐lower rectal cancer underwent preoperative neoadjuvant chemoradiotherapy, while those with liver metastases received preoperative conversion or neoadjuvant therapy. None of the enrolled patients received immunotherapy. After obtaining the patient's informed consent, the biospecimen bank at the researcher's hospital collected peripheral venous blood preoperatively and portal venous blood (venous blood from the tumour area) intraoperatively from patients who underwent primary colorectal cancer resection. For enrolled patients, portal vein blood collection is performed after ligating the arteries and veins in the colorectal tumour region but before tumour resection. Blood is collected by inserting a needle into the vein in the tumour region, with a collection volume of 10–20 mL per patient. The obtained samples were centrifuged and aliquoted within 30 min and stored in a –80°C freezer. Fresh colorectal tumour tissues were kept in vitro for 30 min and stored in a –80°C freezer. Inclusion criteria: colorectal adenocarcinoma, primary tumour resection, with complete clinical specimens (preoperative peripheral blood, intraoperative portal venous blood, colorectal tumour tissue, normal intestinal tissue) and follow‐up data, signed informed consent, follow‐up time is more than 3 years. Patients with hereditary colorectal cancer or a history of other malignant tumours in the past 12 months were excluded. Based on pathology and imaging results, recruited subjects were classified into three groups: (1) Primary colorectal cancer (PC): no distant metastasis at the initial surgery, no liver metastasis after 3 years of follow‐up; (2) patients with colorectal cancer who underwent tumour resection surgery and had concomitant liver metastasis were classified as having synchronous liver metastasis (SLM); and (3) patients with metachronous liver metastasis (MLM): no distant metastasis at the initial surgery, liver metastasis occurred within 3 years after the surgery. A total of 169 subjects were initially recruited. Based on inclusion criteria in addition to similar sex, age, and the appropriate proportion of PC and MLM, 133 samples were enrolled: 70 patients with PC, 31 SLM, and 32 MLM (Scheme S1).

A total of 133 (PC = 70, SLM = 31, MLM = 32) cases of portal venous blood and their matched 64 (PC = 39, SLM = 5, MLM = 20) cases of peripheral blood samples with colorectal cancer patients who underwent surgical resection were collected between 2017 and 2019 in Peking University Shougang Hospital Biobank. In addition, 28 pairs of matched tumour tissues and adjacent normal tissues (PT = 10, PN = 10, SPT = 8, SPN = 8, SMT = 8, SMN = 8, MPT = 10, MPN = 10) were obtained from those patients. All those samples were freshly frozen at −80°C until use. All patients provided written informed consent. Inclusion criteria included diagnosed with colorectal adenocarcinoma by pathological examination; underwent curative surgery. Patients with hereditary colorectal cancer or prior history of other malignancies within the past 12 months were excluded. Peripheral blood samples were collected prior to administration of anaesthesia. Portal venous blood was collected before tumour manipulation from the following draining veins: the inferior mesenteric vein drains the tumour in the descending colon, sigmoid colon, and rectum; the middle colic vein drains the tumour in the transverse colon; and the superior mesenteric vein drains the tumour in the ascending colon. Samples were qualified for the study if tumour tissue had a minimum mass of 200 mg and demonstrated greater than 60% tumour cell nuclei and less than 20% tumour necrosis on frozen tissue section review. Patient history, procedural details, and other relevant clinical and diagnostic information were collected using case report forms. The informed consent was obtained from each patient prior to sample collection, and the study was approved by the Ethics Review Committee of Peking University Shougang Hospital (IRBK‐2019‐020‐02), and this study protocol agrees with the world medical association declaration of Helsinki (2008) and the Belmont Report. All the patients were followed up from the date of surgery until death or the lasted census date (Table S1).

### 5.4 Sample collection and processing

2 mL peripheral blood samples were collected from each patient during their hospitalisation, and the results of clinical tests nearby were recorded. Blood samples collected in lavender‐top EDTA tubes were kept at room temperature for 60 min, followed by two rounds of centrifugation: first for 12 min at 1350 *g*, and then for 5 min at 13 500 *g*. The supernatant (plasma) was collected and stored at −80°C for cfDNA extraction.

### 5.5 Nucleic acid extractions and quantity assessments

cfDNA samples were extracted from .5 mL of plasma using the Quick‐cfDNA Serum & Plasma Kit (ZYMO, D4076) and were resuspended in a final volume of 25 µL of Elution buffer. Frozen tumour gDNA samples were extracted from freshly‐cut 25 mg tissue using the Quick‐DNA miniprep plus kit (ZYMO, D4069) and were resuspended in a final volume of 500 µL of Elution buffer. The quantity of the extracted nucleic acids was assessed using the Qubit™ dsDNA HS Assay kit on a Qubit™ 3.0 Fluorometer.

### 5.6 5hmC‐Seal profiling

Details about the 5hmC‐Seal library construction and bioinformatic processing pipeline are described in previous publications.^89^, ^90^

cfDNA (1–10 ng) or fragmented whole blood genomic DNA (1 µg) spiked with amplicons (.01 pg of each amplicon per 10 ng DNA) was end‐repaired, 3′‐adenylated, and ligated to DNA barcodes (Bioo Scientific) using the KAPA Hyper Prep Kit (Kapa Biosystems) according to the manufacturer's instructions. The ligated DNA was then incubated in a 25 µL solution containing 50 mM HEPES buffer (pH 8), 25 mM MgCl2, 60 µM UDP‐6‐N3‐Glc (Active Motif), and 12.5 U βGT (Thermo) for 2 h at 37°C. After incubation, 2.5 µL of DBCO‐PEG4‐biotin (Click Chemistry Tools, 20 mM stock in DMSO) was added directly to the reaction mixture and incubated for an additional 2 h at 37°C. Subsequently, 10 µg of sheared salmon sperm DNA (Life Technologies) was added, and the DNA was purified using a Micro Bio‐Spin 30 Column (Bio‐Rad). The purified DNA was then incubated with .5 µL of M270 Streptavidin beads (Life Technologies), pre‐blocked with salmon sperm DNA in buffer 1 (5 mM Tris, pH 7.5, .5 mM EDTA, 1 M NaCl, and .2% Tween 20) for 30 min. The beads were washed three times for 5 min each in buffer 1, buffer 2 (buffer 1 without NaCl), buffer 3 (buffer 1, pH 9), and buffer 4 (buffer 3 without NaCl). All binding and washing steps were performed at room temperature with gentle rotation. The beads were then resuspended in water and amplified with 14 cycles (cfDNA) or 9 cycles (whole blood genomic DNA) of PCR using Phusion DNA polymerase (NEB). The PCR products were purified with AMPure XP beads. Separate input libraries were prepared by direct PCR from ligated DNA without labelling and capture. For technical replicates, cfDNA from the same subject was divided into two separate replicates. Paired‐end 75 bp sequencing was performed on the NextSeq instrument.

### 5.7 library sequencing and mapping

Paired‐end 39 bp high‐throughput sequencing was performed on the NextSeq 500 platform. Sequence quality was assessed using FASTQC (version 0.11.5). Raw reads were aligned to the human genome (version hg19) using Bowtie2 (version 2.2.9) and further filtered with SAMtools (version 1.3.1) to retain only unique, non‐duplicate matches to the genome. Paired‐end reads were extended and converted into bedGraph format, normalised to the total number of aligned reads, using BedTools (version 2.19.1). The resulting data were then converted to BigWig format using the bedGraphToBigWig utility from the UCSC Genome Browser for visualisation in the Integrated Genomics Viewer. Potential hMRs were identified using MACS (version 1.4.2), and the parameters used were macs 14‐p 1e‐3‐f BAM‐g hs.^94^ Peak calls were merged using bedtools merge, and only those peak regions that appeared in more than 10 samples and less than 1000 bp were retained. Genomic regions known to exhibit artefact signals, as identified by ENCODE, were also filtered out.

### 5.8 Feature selection, model training, and validation

Colorectal cancer patients were randomly divided into training and validation cohorts with a 2:1 ratio, using train_test_split in Scikit‐Learn (version 0.22.1)^95^ package in Python (version 3.6.10), the logistic regression CV (LR) model was chosen to establish prediction model. In the training cohort, we identified differentially 5hmC enriched Regions (DhMRs) using DESeq2 package (version 1.30.0) in R (version 3.5.0), with the filtering threshold (*p*‐value < .01 & |log2FoldChange| ≥ .5). To avoid overfitting, 5 rounds of 10‐fold cross‐validation was performed. The details were as follows: the training cohort was randomly divided into five folds, four of which were selected as the training subset, and the remaining one was the test subset. Then, we performed 100 repeats to further filtered using the recursive feature elimination algorithm (RFECV) in Scikit‐Learn (parameters used: estimator = LogisticRegressionCV (class_weight = ‘balanced’, cv = 2, max_iter = 1000), scoring = ‘accuracy’). Meanwhile, 10‐fold cross‐validation was repeated 100 times in each round, and the final markers observed in at least 3 rounds were used to build the final warning model in the training cohort. Next, we trained the logistic regression CV model (LR) with the features selected from DhMRs (parameter used: maxiter = 100, method = ‘lbfgs’). Finally, the trained LR model was used to predict liver metastasis in colorectal cancer patients in the validation cohort. Receiver operating characteristics (ROC) analysis was used to evaluate model performance.

### 5.9 Exploring functional relevance of the 5hmC markers

We annotated the DhMRs using the ChIPseeker Package (version 1.20.0),^96^ and DhMGs that were closest to the marker regions were used for the following functional analyses. The GO enrichment analysis was done by the Metascape,^97^ including biological process, Molecular function and Cellular component. Then, the Cytoscape software (version 3.7.2) was used to construct the network.

### 5.10 Cell viability assays

A total of 2000–5000 cells were seeded in 96‐well plates for an overnight culture. The medium was then replaced with fresh medium supplemented with varying concentrations of drugs. After 24 h of incubation, the medium was replaced with fresh medium containing 10 µL of CellTiter 96 Aqueous One Solution Reagent (Promega) per well and incubated at 37°C. Absorbance at 490 nm was measured for subsequent MTS assay analysis to assess cell viability.

### 5.11 Migration and invasion assay

For migration assays (wound healing assays), cells were seeded in 6‐well plates and grown until confluence. Cells were scraped in each well, the medium was replaced with medium with 2% FBS to block cell proliferation. After 24 h or 48 h, the photographs of each well were obtained.

To access cell migration, 1.0  ×  10^5^ cells were seeded into the 8‐µm‐pore upper chambers in serum‐free RPMI1640 and incubated in RPMI1640 with 10% FBS of the lower chamber of 12‐well plates (Corning Star). After normal culture for 24 h, cells were permeabilised with 100% methanol and then stained with .1% crystal violet.

### 5.12 Animal models

A murine colon cancer cell line (MC38) stably transfected with the firefly luciferase (Luc) gene (Addgene plasmid #17477) was generated. To establish a model of liver metastases mice were anaesthetised with ketamine. Then the spleens were exposed to allow direct intrasplenic injections of 5 × 10^5^ Luc‐labelled MC38 (MC38‐Luci) cells in 50 µL 1 mM EDTA in PBS. Mice were sacrificed 18 days after injection of the tumour cells, then spleen and liver were analysed immediately. For ex vivo imaging, female mice were administered by intraperitoneal injection with d‐luciferin (150 mg/kg; Perkin Elmer). Ten minutes later, mice were sacrificed, the livers were collected and acquired using counted using the ex vivo IVIS Lumina III system (Perkin Elmer). Collected images were analysed using Living Image 4.3.1 software (Perkin Elmer): ROIs were drawn around each liver and radiant efficiency calculated.

### 5.13 RNA‐seq and analysis

RNA integrity was assessed using the RNA Nano 6000 Assay Kit on the Bioanalyzer 2100 system (Agilent Technologies, CA, USA). Total RNA was used as input for RNA sample preparation. Briefly, mRNA was purified from total RNA using poly‐T oligo‐attached magnetic beads. Fragmentation was performed using divalent cations at elevated temperature in First Strand Synthesis Reaction Buffer (5X). First‐strand cDNA was synthesised using a random hexamer primer and M‐MuLV Reverse Transcriptase (RNase H‐). Second‐strand cDNA synthesis was then carried out using DNA Polymerase I and RNase H. The remaining overhangs were converted to blunt ends via exonuclease/polymerase activities. After adenylation of the 3′ ends of the DNA fragments, adaptors with a hairpin loop structure were ligated to prepare for hybridisation. To select cDNA fragments in the preferred size range of 370–420 bp, the library fragments were purified using the AMPure XP system (Beckman Coulter, Beverly, USA). PCR amplification was performed using Phusion High‐Fidelity DNA polymerase, universal PCR primers, and an index (X) primer. The PCR products were then purified again using the AMPure XP system, and the library quality was assessed on the Agilent Bioanalyzer 2100 system. Index‐coded samples were clustered on a cBot Cluster Generation System using the TruSeq PE Cluster Kit v3‐cBot‐HS (Illumina) according to the manufacturer's instructions. After cluster generation, the library preparations were sequenced on the Illumina NovaSeq platform, generating 150 bp paired‐end reads.

### 5.14 PDE4D knockdown

To establish short hairpin (sh) PDE4D knockdown cell lines, we use shRNA approach and the shRNA sequences were shown as follows, hPDE4D‐sh: 5′‐GCCAGTGATATACACGGAGATctcgagATCTCCGTGTATATCACTGGCTTTTT, and a non‐specific shRNA control, shRNAC: 5′‐CAACACAGATGATAGAGCACCAATTGGTGCTCTATCATCTGTGTTGTTTTT. As following, the shRNA sequences were constructed into PLKO.1‐puro plasmid. To produce lentiviruses, 293T cells were transfected with a combination of plasmids containing shRNA of PDE4D, pMDLg‐pRRE, pRSV‐rev and pMD2.G. Cell lines were transfected with lentiviruses, and after 72 h, the expression level of PDE4D was analysed by qPCR and immunoblotting. Control group was treated with control lentivirus carrying a non‐specific shRNA.

### 5.15 Gene knockout

To establish PDE4D knockout cell lines, we used the fourth‐generation base editors (BE4) system to change C:G to T:A, and further turned Gln (CAA or CAG) to stop codons (TAA or TAG) then terminated translation. The sgRNA used in our study were: mPDE4D‐sg: 5′‐ AACGGAGCAGGAAGATGTCC. The sgRNA was constructed into mCherry labed BE4 dCas9 vector, and transfected into the MC38 cell by electroporation (Lonza) as their protocol. After 48 h, the mCherry positive cells were selected and seeded into 96 wells plates, ensuring one cell per well, through Flow Cytometry. The monoclonal cells were cultured in CO_2_ incubator normally. To identify genotypeions, the gDNA was extracted from monoclonnal cells using gDNA isolation kits (QIAGEN), and the DNA sequence around the sgRNA targets were obtained by the PCR. The genotypeions were characterised by Sanger Sequencing and the protein level of PDE4D was verified through immunoblotting.

### 5.16 Western blot

Western blot was performed using the following antibodies: anti‐phospho‐ERK (CST, #4370), anti‐ERK (CST, #4695), anti‐phospho‐AKT (CST, #4060), anti‐AKT (CST, #9272), anti‐beta‐tubulin (CST, #86298), anti‐PDE4D (Abcam, ab171750) and anti‐CCN2 (Abcam, ab6992). Cell lysates were separated by electrophoresis on 12% acrylamide SDS gels and transferred to a PVDF membrane. The blots were blocked with 5% non‐fat milk and incubated overnight at 4°C with the primary antibody. Afterward, the blots were incubated for 1 h with the secondary antibody and subsequent imaging was carried out by BIO‐RAD Chemidoc MP imaging system. Relative protein levels were corrected for beta‐tubulin or beta‐actin.

### 5.17 Immunohistochemistry (IHC)

For immunohistochemistry, primary tumour tissues from CRC patients were harvested, rinsed once with PBS, and fixed in 4% paraformaldehyde for 24 h. The tissues were subsequently embedded in paraffin and sectioned into 5 µm slices using a microtome. For immunohistochemical analysis, the sections were deparaffinised. and hydrated with dimethylbenzene and graded concentrations of ethanol. For antigen retrieval, the sections were dipped in 3% H_2_O_2_, deionised water and boiled in citrate buffer orderly. After blocked with 10% goat serum in PBS, sections were stained with PDE4D (Invitrogen, PA5‐79795) according to their standard procedures. Then, secondary antibodies were used, and Images were captured using Olympus FV3000 microscope at × 20 magnification unless specified otherwise.

### 5.18 Immunofluorescence (IF)

The livers from CRC PDX models were harvested, washed once and fixed in 4% paraformaldehyde. After embedding in paraffin blocks, 5 µm sections were obtained using a microtome. The sections were then dewaxed by sequentially incubating twice in 100% xylene, followed by once in 100% ethanol, 90% ethanol, 70% ethanol, and twice in water. The slides were then transferred to sodium citrate buffer for antigen retrieval and incubated in a 95°C water bath. Sections were blocked with 10% goat serum in PBS for 30 min at room temperature; the primary antibodies Vimentin (Cell Signaling Technology, #5741) was diluted in IF buffer (PBS plus 1% bovine serum albumin and 2% fetal bovine serum) overnight at 4°C. After three washes of 5 min in PBS, secondary antibodies diluted in IF buffer were incubated 2 h at room temperature. Slides were mounting mounted with ProLong Gold antifade mounting medium (Invitrogen, P10144) overnight, and were visualised on Olympus FV3000 microscope.

### 5.19 In vitro enzymatic activity assay

Protocols for the expression and enzyme assay of PDE isoforms were similar to our previous works.^98^, ^99^, ^100^ Briefly, the recombinant plasmid was transformed into Escherichia coli competent cells BL21 (CodonPlus) and cultured in LB medium at 37°C until the optical density at 600 nm (A600) reached .6–.8. Induction of protein expression was achieved by adding .1 mM isopropyl β‐D‐thiogalactopyranoside (IPTG), followed by further incubation at 15°C for 24 h. The recombinant proteins were then purified using a Ni‐NTA column (Qiagen), a Q column (GE Healthcare), and a Superdex 100 column (GE Healthcare), and Superdex 100 column (GE Healthcare). The enzymatic activities of PDEs were measured by using ^3^H‐cAMP or ^3^H‐cGMP (GE Healthcare) as substrates. The assay buffer contains 20 mM Tris‐HCl (pH 7.5), 10 mM MgCl_2_ or 4.0 mM MnCl_2_, 1.0 mM dithiothreitol, and 10–30 nM ^3^H‐cAMP or ^3^H‐cGMP (20 000–30 000 cpm/assay). The reaction was conducted at room temperature for 15 min and then terminated by adding .2 M ZnSO4. The reaction products, 3H‐AMP or 3H‐GMP, were precipitated with .2 N Ba(OH)2, while unreacted 3H‐cAMP or 3H‐cGMP remained in the supernatant. The radioactivity of the supernatant was measured in 2.5 mL of Ultima Gold liquid scintillation cocktail (Fisher Scientific) using a PerkinElmer 2910 liquid scintillation counter. For the measurement of IC_50_, eight concentrations at least were used and IC_50_ values were calculated by the nonlinear regression.

### 5.20 Crystallisation and structure determination

The cocrystals of PDE4D‐**L11** was grown by using the hanging drop vapour diffusion method as previously described.^98^, ^101^ The PDE4D^86^, ^87^, ^88^ (10 mg/mL) in a buffer of 50 mM NaCl, 20 mM Tris‐HCl, pH 7.5, 1.0 mM β‐mercaptoethanol, and 1.0 mM ethylenediaminetetraacetic acid was mixed with 10 mM compounds overnight. The precipitant solution was made of .1 M N‐(2‐hydroxyethyl)piperazine‐N′‐ethanesulfonic acid (pH = 7.4), .1 M MgCl_2_, 15% PEG3350, 10% isopropanol, and 25% ethylene glycol, and crystals appeared within a week at 4°C. X‐ray diffraction data were collected at 100 K using an in‐house Rigaku XtaLAB Synergy diffractometer. The data were processed with the CrysAlis Pro software. The structures were solved by molecular replacement using MOLREP, with the PDE4D structure (PDB code: 5WQA) as the search model. The complex structures were then built and refined using Phenix and Coot.^102^, ^103^ The coordinates and structure factors have been deposited in the Protein Data Bank with PDB ID 8XXS, respectively. All structural figures were produced using PyMOL (Ref: The PyMOL Molecular Graphics System). Data collection and refinement statistics for all structures are shown in Table S3.

### 5.21 Emetic assay on beagle dogs

All animal care and experimental protocols were conducted in accordance with the *Guide for the Care and Use of Laboratory Animals* (National Institutes of Health Publication, revised 1996, no. 86‐23, Bethesda, MD) and were approved by the Institutional Animal Research Ethics Committee of Sun Yat‐Sen University. (IACUC numbers: SYSUIACUC‐2021‐000130). The emetic potentials of **L11** and roflumilast were evaluated in beagle dogs,^104^, ^105^ which were divided into two groups and fast for 12 h before the experiment. The six male beagle dogs were purchased from the Guangzhou General Pharmaceutical Research Institute Co., Ltd. Compounds were suspended in .5% carboxymethyl cellulose sodium (CMC‐Na) solution at 1.0 mg/mL and orally administered to dogs with different concentrations (**L11** at 10 mg/kg and roflumilast at 1.0 mg/kg), and then 15 mL of water was used to rinse the lavage tube, ensuring that all the compounds enter the stomach of the dogs. Animals are watched continuously for 180 min for the behaviour of retching, excessive salivation, and vomiting.

### 5.22 Chemistry

All chemicals and reagents were bought from several commercial suppliers (Bide, Adamas, Energy, Sigma‒Aldrich, and J&K) and tested directly without further purification. Silica gel plates with fluorescence F254 (.1‒.2 mm) were performed for thin‐layer chromatography (TLC) analysis, and chemical HG/T2354‐92 silica gel (200‒300 mesh) was carried out for column chromatography. Reactions requiring anhydrous conditions were used under argon or a calcium chloride tube. ¹H NMR and ¹^3^C NMR spectra were recorded on a Bruker AVANCE 400 MHz or 500 MHz NMR spectrometer, using tetramethylsilane as the internal standard. The following abbreviations are used: s (singlet), br (broad signal), d (doublet), dd (doublet of doublets), dt (doublet of triplets), t (triplet), td (triplet of doublets), q (quartet), and m (multiplet). Coupling constants are reported in Hz. High‐resolution mass spectra (HRMS) were obtained using a MAT‐95 spectrometer. Synthesis and characterisation data of targeted compounds were given in Supporting Information.

### 5.23 Statistics

Statistical differences between the two groups were analysed using GraphPad Prism. Data are presented as means ± SEM, unless otherwise specified. *p* Values less than .05 were considered statistically significant for all analyses.

## AUTHOR CONTRIBUTIONS

Sample provision: Jin Gu, Zhaoya Gao, Dandan Huang, Jingyi Shi, Jingyi Shi, and Fuming Lei. 5hmC‐Seal sequencing and data analysis: Hangyu Chen, Lei Zhang, and Zeruo Yang. Pharmacological and pharmacodynamics evaluation: Nuo Xu, Zijian Zhang, Yuchen Wang, Xuyang Lu, Xu Yao, and Xuelan Liu. Cell line construction: Zijian Zhang and Haichuan Zhu. Compounds design and synthesise: Hai‐Bin Luo, Deyan Wu, Yi‐You Huang, Sen Wang, Jinqiang Liang, Can Mao, Feng Zhang, Huimin Xu, Yujiao Wang, and Xian Li. Manuscript writing: Nuo Xu, Zijian Zhang, Hangyu Chen, and Deyan Wu. Manuscript revised: Chuan He, Haichuan Zhu, Hai‐Bin Luo, Jin Gu, and Jian Lin. Supervision: Haichuan Zhu, Hai‐Bin Luo Jin Gu, and Jian Lin.

## CONFLICT OF INTEREST STATEMENT

Nuo Xu, Lei Zhang, Yuchen Wang, Xuyang Lu, Xu Yao, and Xuelan Liu are the employees of Natural Medicine Institute of Zhejiang YangShengTang Co., Ltd. All other authors declare that they have no competing interests.

## ETHICS STATEMENT

This study was approved by the Ethics Research Committee of Peking University Shougang Hospital (IRBK‐2019‐020‐02), and this study protocol agrees with the world medical association declaration of Helsinki (2008) and the Belmont Report. All the patients were followed up from the date of surgery until death or the lasted census date.

## Supporting information



Supporting information

Hepatic portal vein blood collection

## Data Availability

The datasets presented in this study can be found in online repositories. The names of the repository/repositories and accession numbers can be found below, http://bigd.big.ac.cn/gsa‐human, HRA007947. The crystal structures are available at PDB. The remaining data are available within the Article, Supplementary Information or Source Data file.

## References

[1] Siegel RL , Miller KD , Jemal A . Cancer statistics, 2019. CA Cancer J Clin. 2019;69:7‐34. 30620402 10.3322/caac.21551

[2] Dekker E , Tanis PJ , Vleugels JLA , Kasi PM , Wallace MB . Colorectal cancer. Lancet. 2019;394:1467‐1480. 31631858 10.1016/S0140-6736(19)32319-0

[3] Pita‐Fernández S , Alhayek‐Aí M , González‐Martín C , López‐Calviño B , Seoane‐Pillado T , Pértega‐Díaz S . Intensive follow‐up strategies improve outcomes in nonmetastatic colorectal cancer patients after curative surgery: a systematic review and meta‐analysis. Ann Oncol. 2015;26:644‐656. 25411419 10.1093/annonc/mdu543

[4] Bonney GK , Chew CA , Lodge P , et al. Liver transplantation for non‐resectable colorectal liver metastases: the International Hepato‐Pancreato‐Biliary Association consensus guidelines. The lancet Gastroenterology & hepatology. 2021;6:933‐946. 34506756 10.1016/S2468-1253(21)00219-3

[5] Kemeny N . The management of resectable and unresectable liver metastases from colorectal cancer. Curr Opin Oncol. 2010;22:364‐373. 20520544 10.1097/CCO.0b013e32833a6c8a

[6] Al Bandar MH , Kim NK . Current status and future perspectives on treatment of liver metastasis in colorectal cancer (Review). Oncol Rep. 2017;37:2553‐2564. 28350137 10.3892/or.2017.5531

[7] Takahashi H , Berber E . Role of thermal ablation in the management of colorectal liver metastasis. Hepatobil Surg Nutrition. 2020;9:49‐58. 10.21037/hbsn.2019.06.08 PMC7026789 32140478

[8] Zhou H , Liu Z , Wang Y , et al. Colorectal liver metastasis: molecular mechanism and interventional therapy. Signal Transduct Targeted Therap. 2022;7:70. 10.1038/s41392-022-00922-2 PMC8897452 35246503

[9] Tang M , Wang H , Cao Y , Zeng Z , Shan X , Wang L . Nomogram for predicting occurrence and prognosis of liver metastasis in colorectal cancer: a population‐based study. Int J Colorect Dis. 2021;36:271‐282. 10.1007/s00384-020-03722-8 32965529

[10] Biller LH , Schrag D . Diagnosis and treatment of metastatic colorectal cancer: a review. Jama. 2021;325:669‐685. 33591350 10.1001/jama.2021.0106

[11] Li C , Sun Yi‐Di , Yu G‐Y , et al. integrated omics of metastatic colorectal cancer. Cancer Cell. 2020;38:734‐747. e739. 32888432 10.1016/j.ccell.2020.08.002

[12] Oliveira RC , Alexandrino H , Cipriano MA , Alves FC , Tralhão JG . Predicting liver metastases growth patterns: current status and future possibilities. Seminars in cancer biology. 2021;71:42‐51. 32679190 10.1016/j.semcancer.2020.07.007

[13] Tsilimigras DI , Brodt P , Clavien P‐A , et al. Liver metastases. Nat Rev. 2021;7:27. 10.1038/s41572-021-00261-6 33859205

[14] Normanno N , Cervantes A , Ciardiello F , De Luca A , Pinto C . The liquid biopsy in the management of colorectal cancer patients: current applications and future scenarios. Cancer Treat Rev. 2018;70:1‐8. 30053724 10.1016/j.ctrv.2018.07.007

[15] Cannella R , Tselikas L , Douane F , et al. Imaging‐guided interventions modulating portal venous flow: evidence and controversies. JHEP Rep. 2022;4:100484. 35677591 10.1016/j.jhepr.2022.100484 PMC9168703

[16] Subbotin VM . Privileged portal metastasis of hepatocellular carcinoma in light of the coevolution of a visceral portal system and liver in the chordate lineage: a search for therapeutic targets. Drug Discov Today. 2018;23:548‐564. 29330122 10.1016/j.drudis.2018.01.020

[17] Riddiough GE , Jalal Q , Perini MV , Majeed AW . Liver regeneration and liver metastasis. Semin Cancer Biol. 2021;71:86‐97. 32532594 10.1016/j.semcancer.2020.05.012

[18] Catenacci DVT , Chapman CG , Xu P , et al. Acquisition of portal venous circulating tumor cells from patients with pancreaticobiliary cancers by endoscopic ultrasound. Gastroenterology. 2015;149:1794‐1803. e1794. 26341722 10.1053/j.gastro.2015.08.050 PMC4985007

[19] Chapman CG , Ayoub F , Swei E , et al. Endoscopic ultrasound acquired portal venous circulating tumor cells predict progression free survival and overall survival in patients with pancreaticobiliary cancers. Pancreatology. 2020;20:1747‐1754. 33082106 10.1016/j.pan.2020.10.039

[20] Zhang Y , Su H , Wang H , et al. Endoscopic ultrasound‐guided acquisition of portal venous circulating tumor cells as a potential diagnostic and prognostic tool for pancreatic cancer. Cancer Manag Res. 2021;13:7649‐7661. 34675662 10.2147/CMAR.S330473 PMC8502022

[21] Jiao LR , Apostolopoulos C , Jacob J , et al. Unique localization of circulating tumor cells in patients with hepatic metastases. J Clin Oncol. 2009;27:6160‐6165. 19884529 10.1200/JCO.2009.24.5837

[22] Chen H‐Yu , Zhang W‐L , Zhang L , et al. 5‐Hydroxymethylcytosine profiles of cfDNA are highly predictive of R‐CHOP treatment response in diffuse large B cell lymphoma patients. Clin Epigenetics. 2021;13:33. 33573703 10.1186/s13148-020-00973-8 PMC7879534

[23] Li W , Zhang Xu , Lu X , et al. 5‐Hydroxymethylcytosine signatures in circulating cell‐free DNA as diagnostic biomarkers for human cancers. Cell Res. 2017;27:1243‐1257. 28925386 10.1038/cr.2017.121 PMC5630683

[24] Song C‐X , Yin S , Ma Li , et al. 5‐Hydroxymethylcytosine signatures in cell‐free DNA provide information about tumor types and stages. Cell Res. 2017;27:1231‐1242. 28820176 10.1038/cr.2017.106 PMC5630676

[25] Cui X‐L , Nie Ji , Ku J , et al. A human tissue map of 5‐hydroxymethylcytosines exhibits tissue specificity through gene and enhancer modulation. Nat Commun. 2020;11:6161. 33268789 10.1038/s41467-020-20001-w PMC7710742

[26] Cai J , Chen L , Zhang Z , et al. Genome‐wide mapping of 5‐hydroxymethylcytosines in circulating cell‐free DNA as a non‐invasive approach for early detection of hepatocellular carcinoma. Gut. 2019;68:2195‐2205. 31358576 10.1136/gutjnl-2019-318882 PMC6872444

[27] He B , Zhang C , Zhang X , et al. Tissue‐specific 5‐hydroxymethylcytosine landscape of the human genome. Nat Commun. 2021;12:4249. 34253716 10.1038/s41467-021-24425-w PMC8275684

[28] Guler GD , Ning Y , Ku C‐J , et al. Detection of early stage pancreatic cancer using 5‐hydroxymethylcytosine signatures in circulating cell free DNA. Nat Commun. 2020;11:5270. 33077732 10.1038/s41467-020-18965-w PMC7572413

[29] Chen H‐Yu , Li X‐X , Li C , et al. 5‐Hydroxymethylcytosine signatures in circulating cell‐free dna as early warning biomarkers for COVID‐19 progression and myocardial injury. Front Cell Dev Biol. 2021;9:781267. 35071229 10.3389/fcell.2021.781267 PMC8770986

[30] Tian X , Sun B , Chen C , et al. Circulating tumor DNA 5‐hydroxymethylcytosine as a novel diagnostic biomarker for esophageal cancer. Cell Res. 2019;28:597‐600. 10.1038/s41422-018-0014-x PMC5951904 29467383

[31] Yang Y , Zeng C , Lu X , et al. 5‐Hydroxymethylcytosines in circulating cell‐free DNA reveal vascular complications of type 2 diabetes. Clin Chem. 2019;65:1414‐1425. 31575611 10.1373/clinchem.2019.305508 PMC7055673

[32] Dong C , Chen J , Zheng J , et al. 5‐Hydroxymethylcytosine signatures in circulating cell‐free DNA as diagnostic and predictive biomarkers for coronary artery disease. Clin Epigenetics. 2020;12:17. 31964422 10.1186/s13148-020-0810-2 PMC6974971

[33] Branco MR , Ficz G , Reik W . Uncovering the role of 5‐hydroxymethylcytosine in the epigenome. Nat Rev. 2011;13:7‐13. 10.1038/nrg3080 22083101

[34] Quail DF , Joyce JA . Microenvironmental regulation of tumor progression and metastasis. Nat Med. 2013;19:1423‐1437. 24202395 10.1038/nm.3394 PMC3954707

[35] Cioroianu AI , Stinga PI , Sticlaru L , et al. Tumor microenvironment in diffuse large B‐cell lymphoma: role and prognosis. Analyt Cell Pathol (Amsterdam). 2019;2019:8586354. 10.1155/2019/8586354 PMC6942707 31934533

[36] Roma‐Rodrigues C , Mendes R , Baptista PV , Fernandes AR . Targeting tumor microenvironment for cancer therapy. Int J Mol Sci. 2019;20. 10.3390/ijms20040840 PMC6413095 30781344

[37] Mani DR , Krug K , Zhang B , et al. Cancer proteogenomics: current impact and future prospects. Nat Rev. 2022;22:298‐313. 10.1038/s41568-022-00446-5 PMC12404316 35236940

[38] Lin W‐H , Chang Yi‐W , Hong M‐X , et al. STAT3 phosphorylation at Ser727 and Tyr705 differentially regulates the EMT‐MET switch and cancer metastasis. Oncogene. 2021;40:791‐805. 33262462 10.1038/s41388-020-01566-8 PMC7843420

[39] Liu B , Sun L , Liu Q , et al. A cytoplasmic NF‐κB interacting long noncoding RNA blocks IκB phosphorylation and suppresses breast cancer metastasis. Cancer Cell. 2015;27:370‐381. 25759022 10.1016/j.ccell.2015.02.004

[40] Zhang J , Wang S , Jiang B , et al. c‐Src phosphorylation and activation of hexokinase promotes tumorigenesis and metastasis. Nat Commun. 2017;8:13732. 28054552 10.1038/ncomms13732 PMC5227066

[41] Xu RX , Hassell AM , Vanderwall D , et al. Atomic structure of PDE4: insights into phosphodiesterase mechanism and specificity. Science (New York, NY). 2000;288:1822‐1825. 10.1126/science.288.5472.1822 10846163

[42] Pérez‐Torres S , Miró X , Palacios JM , Cortés R , Puigdoménech P , Mengod G . Phosphodiesterase type 4 isozymes expression in human brain examined by in situ hybridization histochemistry and[3H]rolipram binding autoradiography. Comparison with monkey and rat brain. J Chem Neuroanat. 2000;20:349‐374. 11207431 10.1016/s0891-0618(00)00097-1

[43] Lakics V , Karran EH , Boess FG . Quantitative comparison of phosphodiesterase mRNA distribution in human brain and peripheral tissues. Neuropharmacology. 2010;59:367‐374. 20493887 10.1016/j.neuropharm.2010.05.004

[44] Contreras S , Milara J , Morcillo E , Cortijo J . Selective inhibition of phosphodiesterases 4A, B, C and D isoforms in chronic respiratory diseases: current and future evidences. Curr Pharmaceut Design. 2017;23:2073‐2083. 10.2174/1381612823666170214105651 28201975

[45] Tran CM , Fujita N , Huang B‐L , et al. Hypoxia‐inducible factor (HIF)‐1α and CCN2 form a regulatory circuit in hypoxic nucleus pulposus cells: CCN2 suppresses HIF‐1α level and transcriptional activity. J Biol Chem. 2013;288:12654‐12666. 23530034 10.1074/jbc.M112.448860 PMC3642312

[46] Wu Z , Zhou C , Yuan Q , Zhang D , Xie J , Zou S . CTGF facilitates cell‐cell communication in chondrocytes via PI3K/Akt signalling pathway. Cell Prolifer. 2021;54:e13001. 10.1111/cpr.13001 PMC7941231 33522639

[47] Hashiguchi S , Tanaka T , Mano R , Kondo S , Kodama S . CCN2‐induced lymphangiogenesis is mediated by the integrin αvβ5‐ERK pathway and regulated by DUSP6. Sci Rep. 2022;12:926. 35042954 10.1038/s41598-022-04988-4 PMC8766563

[48] Yang Y , Tan Y‐X , Chen R‐Y , Kang J . The latest review on the polyphenols and their bioactivities of Chinese Morus plants. J Asian Nat Prod Res. 2014;16:690‐702. 24911924 10.1080/10286020.2014.923405

[49] Chen S‐Ke , Zhao P , Shao Y‐X , et al. *Moracin* M from *Morus alba* L. is a natural phosphodiesterase‐4 inhibitor. Bioorg Med Chem Lett. 2012;22:3261‐3264. 22483586 10.1016/j.bmcl.2012.03.026

[50] Huang Y , Liu X , Wu D , et al. The discovery, complex crystal structure, and recognition mechanism of a novel natural PDE4 inhibitor from *Selaginella pulvinata* . Biochem Pharmacols. 2017;130:51‐59. 10.1016/j.bcp.2017.01.016 28159622

[51] Meyer Y , Olthof PB , Grünhagen DJ , et al. Treatment of metachronous colorectal cancer metastases in the Netherlands: a population‐based study. Eur J Surg Oncol. 2022;48:1104‐1109. 34895970 10.1016/j.ejso.2021.12.004

[52] Zviran A , Schulman RC , Shah M , et al. Genome‐wide cell‐free DNA mutational integration enables ultra‐sensitive cancer monitoring. Nat Med. 2020;26:1114‐1124. 32483360 10.1038/s41591-020-0915-3 PMC8108131

[53] Pantel K , Alix‐Panabières C . Circulating tumour cells in cancer patients: challenges and perspectives. Trends Mol Med. 2010;16:398‐406. 20667783 10.1016/j.molmed.2010.07.001

[54] Ignatiadis M , Sledge GW , Jeffrey SS . Liquid biopsy enters the clinic—implementation issues and future challenges. Nat Rev Clin Oncol. 2021;18:297‐312. 33473219 10.1038/s41571-020-00457-x

[55] Bachman M , Uribe‐Lewis S , Yang X , Williams M , Murrell A , Balasubramanian S . 5‐Hydroxymethylcytosine is a predominantly stable DNA modification. Nat Chem. 2014;6:1049‐1055. 25411882 10.1038/nchem.2064 PMC4382525

[56] Mooijman D , Dey SS , Boisset J‐C , Crosetto N , Van Oudenaarden A . Single‐cell 5hmC sequencing reveals chromosome‐wide cell‐to‐cell variability and enables lineage reconstruction. Nat Biotechnol. 2016;34:852‐856. 27347753 10.1038/nbt.3598

[57] Liu L , Toung JM , Jassowicz AF , et al. Targeted methylation sequencing of plasma cell‐free DNA for cancer detection and classification. Ann Oncol. 2018;29:1445‐1453. 29635542 10.1093/annonc/mdy119 PMC6005020

[58] Shen SYi , Singhania R , Fehringer G , et al. Sensitive tumour detection and classification using plasma cell‐free DNA methylomes. Nature. 2018;563:579‐583. 30429608 10.1038/s41586-018-0703-0

[59] Chapman CG , Waxman I . EUS‐guided portal venous sampling of circulating tumor cells. Curr Gastroenterol Rep. 2019;21:68. 31813055 10.1007/s11894-019-0733-2

[60] Yang X , Bi X , Liu F , Huang J , Zhang Z . predictive efficacy of circulating tumor cells in first drainage vein blood from patients with colorectal cancer liver metastasis. Cancer Investig. 2022:1‐10. 10.1080/07357907.2022.2098970 35797354

[61] Engstrand J , Strömberg C , Nilsson H , Freedman J , Jonas E . Synchronous and metachronous liver metastases in patients with colorectal cancer‐towards a clinically relevant definition. World J Surg Oncol. 2019;17:228. 31878952 10.1186/s12957-019-1771-9 PMC6933908

[62] Van Den Eynden GG , Majeed AW , Illemann M , et al. The multifaceted role of the microenvironment in liver metastasis: biology and clinical implications. Cancer Res. 2013;73:2031‐2043. 23536564 10.1158/0008-5472.CAN-12-3931

[63] Li W , Guo L , Tang W , et al. Identification of DNA methylation biomarkers for risk of liver metastasis in early‐stage colorectal cancer. Clin Epigenetics. 2021;13:126. 34108011 10.1186/s13148-021-01108-3 PMC8190869

[64] Hao M , Wang K , Ding Y , Li H , Liu Y , Ding L . Which patients are prone to suffer liver metastasis? A review of risk factors of metachronous liver metastasis of colorectal cancer. Euro J Med Res. 2022;27:130. 10.1186/s40001-022-00759-z PMC9310475 35879739

[65] Xiao C , Zhou M , Yang X , et al. Accurate prediction of metachronous liver metastasis in Stage I‐III colorectal cancer patients using deep learning with digital pathological images. Front Oncol. 2022;12:844067. 35433467 10.3389/fonc.2022.844067 PMC9010865

[66] Taghavi M , Trebeschi S , Simões R , et al. Machine learning‐based analysis of CT radiomics model for prediction of colorectal metachronous liver metastases. Abdominal Radiol (New York). 2021;46:249‐256. 10.1007/s00261-020-02624-1 32583138

[67] Li Y , Gong J , Shen X , et al. Assessment of primary colorectal cancer CT radiomics to predict metachronous liver metastasis. Front Oncol. 2022;12:861892. 35296011 10.3389/fonc.2022.861892 PMC8919043

[68] Armaghany T , et al. Genetic alterations in colorectal cancer. Gastrointestinal cancer Res. 2012;5:19‐27. PMC3348713 22574233

[69] Zeng X , Ward SE , Zhou J , Cheng ASL . Cheng, liver immune microenvironment and metastasis from colorectal cancer‐pathogenesis and therapeutic perspectives. Cancers. 2021;13. 10.3390/cancers13102418 PMC8156220 34067719

[70] Pretzsch E , Bösch F , Neumann J , et al. Mechanisms of metastasis in colorectal cancer and metastatic organotropism: hematogenous versus peritoneal spread. J Oncol. 2019;2019:7407190. 31641356 10.1155/2019/7407190 PMC6770301

[71] Zaccolo M , Zerio A , Lobo MJ , Garland C . Subcellular organization of the cAMP signaling pathway. Pharmacol Rev. 2021;73:278‐309. 33334857 10.1124/pharmrev.120.000086 PMC7770493

[72] Zhang F , Zhang L , Qi Y , Xu H . Mitochondrial cAMP signaling. Cell Mol Life Sci. 2016;73:4577‐4590. 27233501 10.1007/s00018-016-2282-2 PMC5097110

[73] Dua P , Gude RP . Pentoxifylline impedes migration in B16F10 melanoma by modulating Rho GTPase activity and actin organisation. Eur J Cancer. 2008;44:1587‐1595. 18495474 10.1016/j.ejca.2008.04.009

[74] Zimmerman NP , Roy I , Hauser AD , Wilson JM , Williams CL , Dwinell MB . Cyclic AMP regulates the migration and invasion potential of human pancreatic cancer cells. Mol Carcinog. 2015;54:203‐215. 24115212 10.1002/mc.22091 PMC4087083

[75] Jiang Ke , Yao G , Hu L , et al. MOB2 suppresses GBM cell migration and invasion via regulation of FAK/Akt and cAMP/PKA signaling. Cell Death Dis. 2020;11:230. 32286266 10.1038/s41419-020-2381-8 PMC7156523

[76] Yang F , Tuxhorn JA , Ressler SJ , Mcalhany SJ , Dang TD , Rowley DR . Stromal expression of connective tissue growth factor promotes angiogenesis and prostate cancer tumorigenesis. Cancer Res. 2005;65:8887‐8895. 16204060 10.1158/0008-5472.CAN-05-1702

[77] Dornhöfer N , Spong S , Bennewith K , et al. Connective tissue growth factor‐specific monoclonal antibody therapy inhibits pancreatic tumor growth and metastasis. Cancer Res. 2006;66:5816‐5827. 16740721 10.1158/0008-5472.CAN-06-0081

[78] Sha W , Leask A . CCN2 expression and localization in melanoma cells. J Cell Commun Signaling. 2011;5:219‐226. 10.1007/s12079-011-0128-0 PMC3145873 21667293

[79] Ubink I , Verhaar ER , Kranenburg O , Goldschmeding R . A potential role for CCN2/CTGF in aggressive colorectal cancer. J Cell Commun Signal. 2016;10:223‐227. 27613407 10.1007/s12079-016-0347-5 PMC5055504

[80] Lin B‐R , Chang C‐C , Chen RJ‐C , et al. Connective tissue growth factor acts as a therapeutic agent and predictor for peritoneal carcinomatosis of colorectal cancer. *Clin* Cancer Res. 2011;17:3077‐3088. 10.1158/1078-0432.CCR-09-3256 21558398

[81] Guo Y , Li X , Lin C , et al. MicroRNA‑133b inhibits connective tissue growth factor in colorectal cancer and correlates with the clinical stage of the disease. Mol Med Rep. 2015;11:2805‐2812. 25501363 10.3892/mmr.2014.3075

[82] Pierobon M , Ramos C , Wong S , et al. Enrichment of PI3K‐AKT‐mTOR pathway activation in hepatic metastases from breast cancer. Clin Cancer Res. 2017;23:4919‐4928. 28446508 10.1158/1078-0432.CCR-16-2656 PMC5564311

[83] Zhang Y , Cheng H , Li W , Wu H , Yang Y . Highly‐expressed P2X7 receptor promotes growth and metastasis of human HOS/MNNG osteosarcoma cells via PI3K/Akt/GSK3β/β‐catenin and mTOR/HIF1α/VEGF signaling. Int J Cancer. 2019;145:1068‐1082. 30761524 10.1002/ijc.32207 PMC6618011

[84] Wen S , Hou Y , Fu L , et al. Cancer‐associated fibroblast (CAF)‐derived IL32 promotes breast cancer cell invasion and metastasis via integrin β3‐p38 MAPK signalling. Cancer Lett. 2019;442:320‐332. 30391782 10.1016/j.canlet.2018.10.015

[85] Xiang Z , Li J , Song S , et al. A positive feedback between IDO1 metabolite and COL12A1 via MAPK pathway to promote gastric cancer metastasis. J Exp Clin Cancer Res: CR. 2019;38:314. 31315643 10.1186/s13046-019-1318-5 PMC6637527

[86] Garnock‐Jones KP . Roflumilast: a review in COPD. Drugs. 2015;75:1645‐1656. 26338438 10.1007/s40265-015-0463-1

[87] Rabe KF . Update on roflumilast, a phosphodiesterase 4 inhibitor for the treatment of chronic obstructive pulmonary disease. Br J Pharmacol. 2011;163:53‐67. 21232047 10.1111/j.1476-5381.2011.01218.x PMC3085868

[88] Rabe KF , Bateman ED , O'donnell D , Witte S , Bredenbröker D , Bethke TD . Roflumilast–an oral anti‐inflammatory treatment for chronic obstructive pulmonary disease: a randomised controlled trial. Lancet. 2005;366:563‐571. 16099292 10.1016/S0140-6736(05)67100-0

[89] Song C‐X , Szulwach KE , Fu Ye , et al. Selective chemical labeling reveals the genome‐wide distribution of 5‐hydroxymethylcytosine. Nat Biotechnol. 2011;29:68‐72. 21151123 10.1038/nbt.1732 PMC3107705

[90] Song C‐X , Yin S , Ma Li , et al. 5‐Hydroxymethylcytosine signatures in cell‐free DNA provide information about tumor types and stages. Cell Res. 2017;27:1231‐1242. 28820176 10.1038/cr.2017.106 PMC5630676

[91] Langmead B , Salzberg SL . Fast gapped‐read alignment with Bowtie 2. Nat Methods. 2012;9:357‐359. 22388286 10.1038/nmeth.1923 PMC3322381

[92] Li H , Handsaker B , Wysoker A , et al. The Sequence Alignment/Map format and SAMtools. Bioinformatics (Oxford, England). 2009;25:2078‐2079. 19505943 10.1093/bioinformatics/btp352 PMC2723002

[93] Quinlan AR . BEDTools: the Swiss‐Army tool for genome feature analysis. Curr Protocol Bioinform. 2014;47. 11.12.11‐34. 10.1002/0471250953.bi1112s47 PMC4213956 25199790

[94] ENCODE Project Consortium , Birney E , Stamatoyannopoulos JA , et al, ENCODE Project Consortium . Identification and analysis of functional elements in 1% of the human genome by the ENCODE pilot project. Nature. 2007;447:799‐816. 17571346 10.1038/nature05874 PMC2212820

[95] Swami A , et al. Scikit‐learn: machine Learning in Python. J Mach Learning Res∖. 2013;12:2825‐2830.

[96] Yu G , Wang LG , He QY , et al. ChIPseeker: an R/Bioconductor package for ChIP peak annotation, comparison and visualization. Bioinformatics (Oxford, England). 2015;31:2382‐2383. 25765347 10.1093/bioinformatics/btv145

[97] Zhou Y , Zhou B , Pache L , et al. Metascape provides a biologist‐oriented resource for the analysis of systems‐level datasets. Nat Commun. 2019;10:1523. 30944313 10.1038/s41467-019-09234-6 PMC6447622

[98] Huang Y , Liu X , Wu D , et al. The discovery, complex crystal structure, and recognition mechanism of a novel natural PDE4 inhibitor from Selaginella pulvinata. Biochem Pharmacol. 2017;130:51‐59. 28159622 10.1016/j.bcp.2017.01.016

[99] Huang Yi‐Y , Yu Y‐Fa , Zhang C , et al. Validation of Phosphodiesterase‐10 as a novel target for pulmonary arterial hypertension via highly selective and subnanomolar inhibitors. J Med Chem. 2019;62:3707‐3721. 30888810 10.1021/acs.jmedchem.9b00224

[100] Zhang T , Lai Z , Yuan S , et al. Discovery of evodiamine derivatives as highly selective PDE5 inhibitors targeting a unique allosteric pocket. J Med Chem. 2020;63:9828‐9837. 32794708 10.1021/acs.jmedchem.0c00983

[101] Liang J , Huang Yi‐Y , Zhou Q , et al. Discovery and optimization of α‐mangostin derivatives as novel PDE4 inhibitors for the treatment of vascular dementia. J Med Chem. 2020;63:3370‐3380. 32115956 10.1021/acs.jmedchem.0c00060

[102] Adams PD , Afonine PV , Bunkóczi G , et al. PHENIX: a comprehensive Python‐based system for macromolecular structure solution. Acta crystallographica. 2010;66:213‐221. 10.1107/S0907444909052925 PMC2815670 20124702

[103] Emsley P , Cowtan K . Coot: model‐building tools for molecular graphics. Acta crystallographica. 2004;60:2126‐2132. 15572765 10.1107/S0907444904019158

[104] Dastidar SG , Ray A , Shirumalla R , et al. Pharmacology of a novel, orally active PDE4 inhibitor. Pharmacology. 2009;83:275‐286. 19321962 10.1159/000209608

[105] Nose T , Kondo M , Shimizu M , et al. Pharmacological profile of GPD‐1116, an inhibitor of phosphodiesterase 4. Biol Pharmaceut Bull. 2016;39:689‐698. 10.1248/bpb.b15-00652 27150141

[106] Total synthesis of the 2‐arylbenzo[b]furan‐containing natural products from Artocarpus. Tetrahedr Lett. 2015;56:4383‐4387.

