# Supplementary material for: 5‐hydroxymethylcytosine features of portal venous blood predict metachronous liver metastases of colorectal cancer and reveal phosphodiesterase 4 as a therapeutic target
Source: Clin Transl Med. 2025 Feb 16;15(2):e70189. doi: 10.1002/ctm2.70189 (PMC11830572; doi:10.1002/ctm2.70189)
Supplement: Supplementary file 1 — Supporting information [file CTM2-15-e70189-s002.docx]

5-hydroxymethylcytosine features of portal venous blood predict metachronous liver metastases of colorectal cancer and reveal phosphodiesterase 4 as a therapeutic target

Nuo Xu^1,2†^, Zhaoya Gao^3,11†^, Deyan Wu^4†^, Hangyu Chen^1†^, Zijian Zhang^4†^, Lei Zhang^2^, Yuchen Wang^2^, Xuyang Lu^2^, Xu Yao^2^, Xuelan Liu^2^, Yi-You Huang^4^, Meiying Qiu^14^, Sen Wang^4^, Jinqiang Liang^4^, Can Mao^4^, Feng Zhang^4^, Huimin Xu^4^, Yujiao Wang^4^, Xian Li^4^, Zhexin Chen^4^, Dandan Huang^9,11^, Jingyi Shi^10^, Wensheng Huang^3,11^, Fuming Lei^3,11^, Zeruo Yang^1^, Long Chen^1^, Chuan He^13^, Haichuan Zhu^5*^, Hai-Bin Luo^4*^, Jin Gu^3,10,11,12*^ and Jian Lin^1,6,7,8*^

**Affiliations:**

^1^Department of Pharmacy, Peking University Third Hospital, Beijing 100191, China.

^2^Natural Medicine Institute of Zhejiang YangShengTang Co. Ltd., Hangzhou 310024, China.

^3^Department of Gastrointestinal Surgery, Peking University Shougang Hospital, Beijing, China.

^4^Key Laboratory of Tropical Biological Resources of Ministry of Education, School of Pharmaceutical Sciences, Hainan University, Haikou 570228, Hainan, China.

^5^Institute of Biology and Medicine, College of Life and Health Sciences, Wuhan University of Science and Technology, Wuhan 430081, China.

^6^ Peking University Third Hospital Cancer Center, Beijing 100191, China.

^7^Synthetic and Functional Biomolecules Center, Peking University, Beijing 100871, China.

^8^Song Li’s Academician Workstation of Hainan University (School of Pharmaceutical Science), Yazhou Bay, Sanya 572000, China.

^9^Department of Oncology, Peking University Shougang Hospital, Beijing, China.

^10^Key laboratory of Carcinogenesis & Translational Research (Ministry of Education), Department of Gastrointestinal Surgery III, Peking University Cancer Hospital & Institute, Beijing, 100142, China.

^11^Center for Precision Diagnosis and Treatment of Colorectal Cancer and Inflammatory Diseases，Peking University Health Science Center, Beijing, China.

^12^Peking University International Cancer Institute, Beijing, China

^13^Department of Chemistry, Department of Biochemistry and Molecular Biology, Howard Hughes Medical Institute, The University of Chicago, Chicago, IL 60637, USA.

^14^School of Pharmaceutical Sciences, Sun Yat-sen University, Guangzhou 510006, China

† These authors contribute equally to this work

* Corresponding author. Email:

Jian Lin: linjian@pku.edu.cn

Jin Gu: zlguj@bjmu.edu.cn

Hai-Bin Luo: [hbluo@hainanu.edu.cn](mailto:hbluo@hainanu.edu.cn)

Haichuan Zhu: zhuhaichuan@wust.edu.cn

**
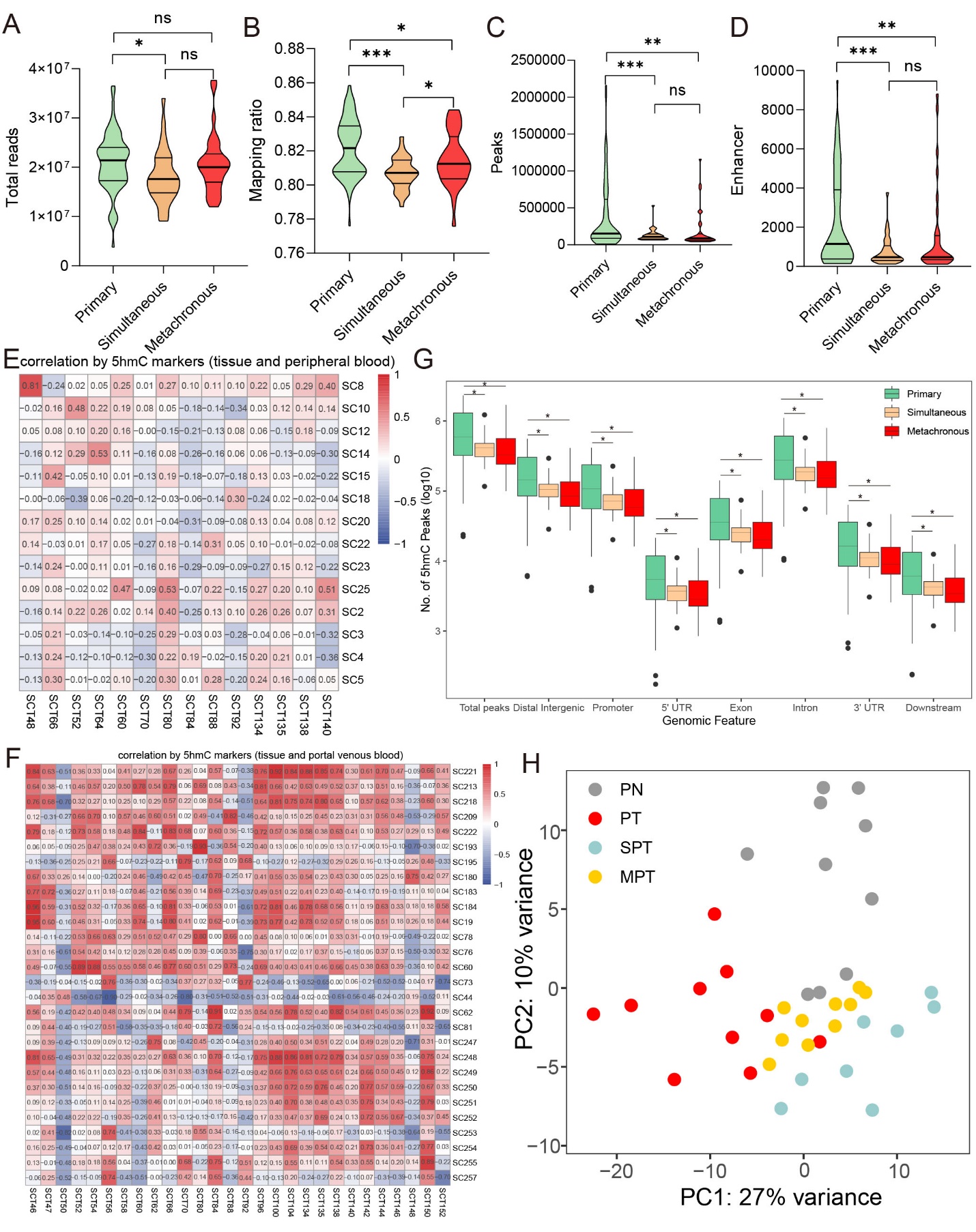
**

**Supplementary Fig. 1. QC analysis for 5hmC-Seal data.** (A) Total reads number for the groups. Each boxplot represents all the samples of each group. (B) Mapping ratios of the samples. Each boxplot represents all the samples of each group. (C) Total peaks number for the groups. (D) Peaks number enriched regions in enhancer for the groups. (E) Within-subject correlation is significantly higher (Wilcoxon rank-sum test p<0.01) between peripheral blood cfDNA and tissue genomic DNA than that between different individuals, based on the top variable genes in cfDNA samples in terms of 5hmC modification. (F) Within-subject correlation is significantly higher (Wilcoxon rank-sum test p<0.01) between portal venous blood cfDNA and tumor tissue genomic DNA than that between different individuals, based on the top variable genes in cfDNA samples in terms of 5hmC modification. (G) Genome-wide 5hmC distribution in different genomic features grouped by CRC patients (*p<0.05). (H) Principal component analysis (PCA) plot based on top variance hMRs in tissue gDNA samples.


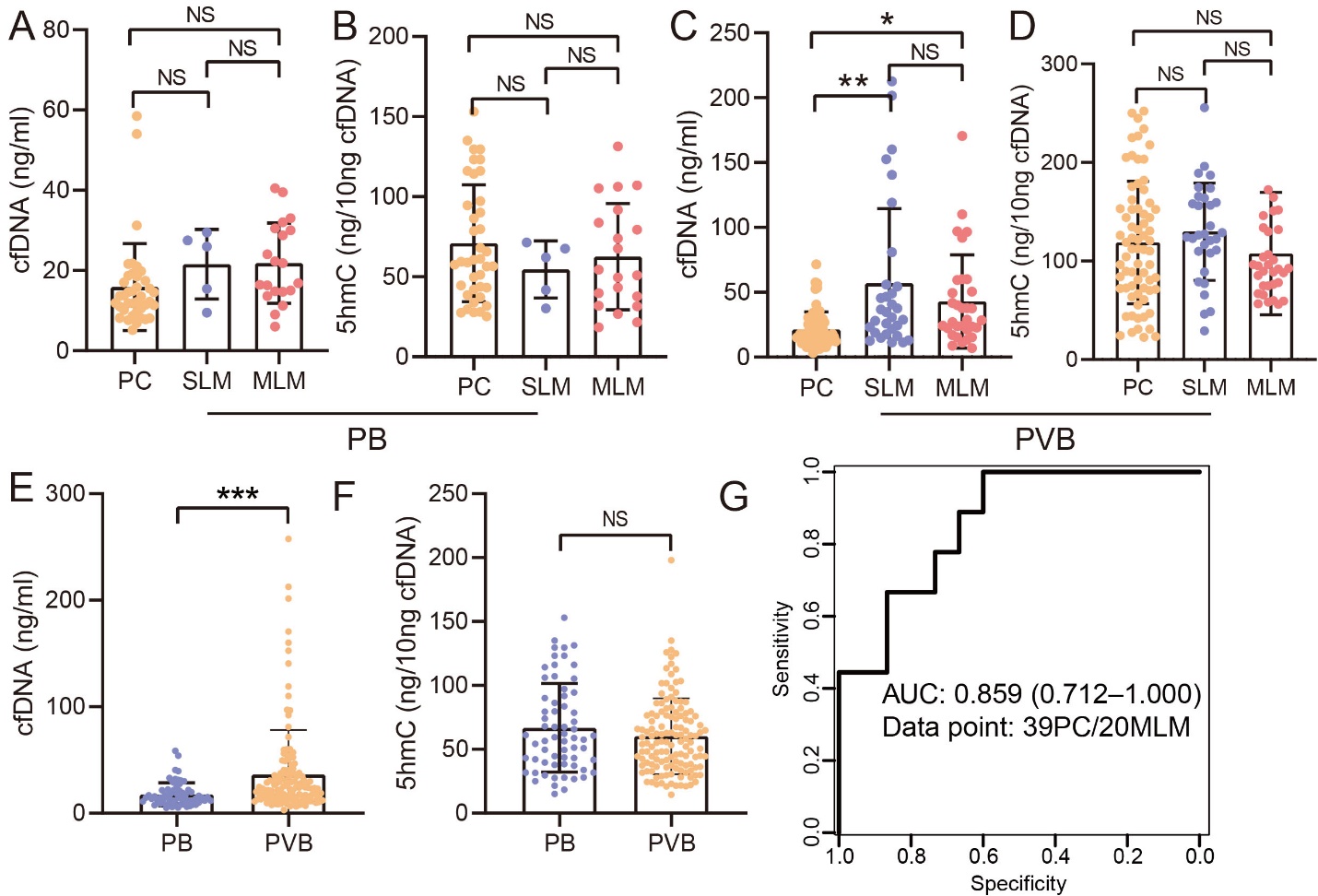


**Supplementary Fig. 2. Comparative analysis of portal venous blood and peripheral blood.** (A) cfDNA content in peripheral blood. (B) 5hmC content in peripheral blood. (C) cfDNA content in portal vein blood. (D) 5hmC content in portal vein blood. (E) cfDNA content in portal vein blood (PVB) and peripheral blood (PB). (F) 5hmC content in portal vein blood (PVB) and peripheral blood (PB). (G) Receiver operating characteristic (ROC) curve of the classification model with 5hmC markers in the PB samples. The true positive rate (sensitivity) is plotted in function of the false positive rate (1-specificity).

**
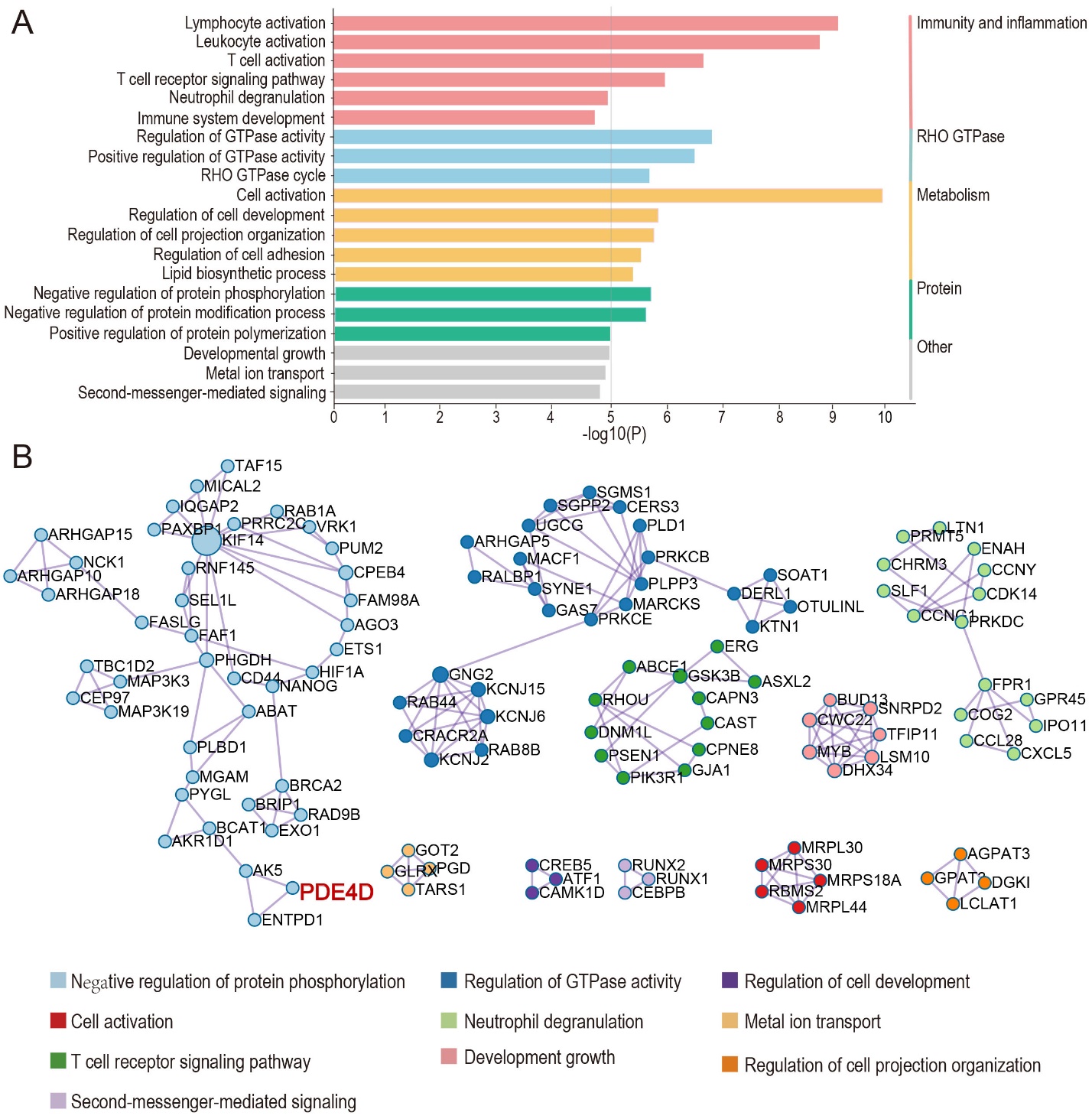
**

**Supplementary Fig. 3. GO enrichment analysis and function exploration of DhMGs.** (A) Metascape bar graph for viewing top non-redundant enrichment clusters, one per cluster, using bar graph length to represent statistical significance. (B) Ten MCODE complexes automatically identified in Metascape, colored according to their identities. Their functional labels are generated based on the top-three functional enriched terms, if visualizations of meta-analysis results based on multiple gene lists are available.


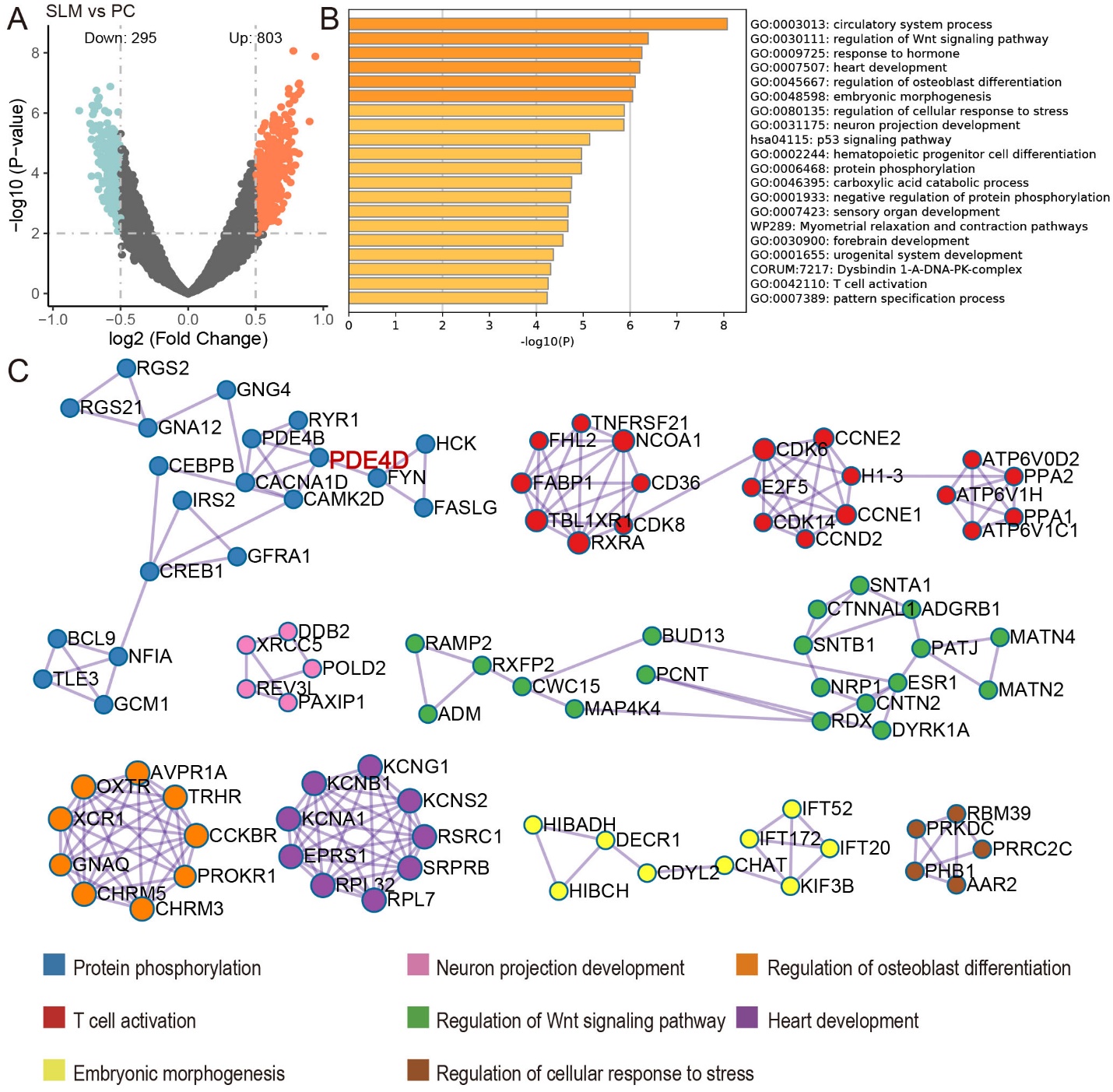


**Supplementary Fig. 4. GO enrichment analysis and function exploration of DhMGs using Metascape.** (A) Volcano plot of hMRs (SLM patients vs PC patients). Significance: (|log2FoldChange| ≥ 0.5 and p-value < 0.01). (B) Metascape bar graph for viewing top non-redundant enrichment clusters, one per cluster, using bar graph length to represent statistical significance. (C) Ten MCODE complexes automatically identified in Metascape, colored by their identities. Their functional labels are generated based on the top-three functional enriched terms, if available visualizations of meta-analysis results based on multiple gene lists.

**
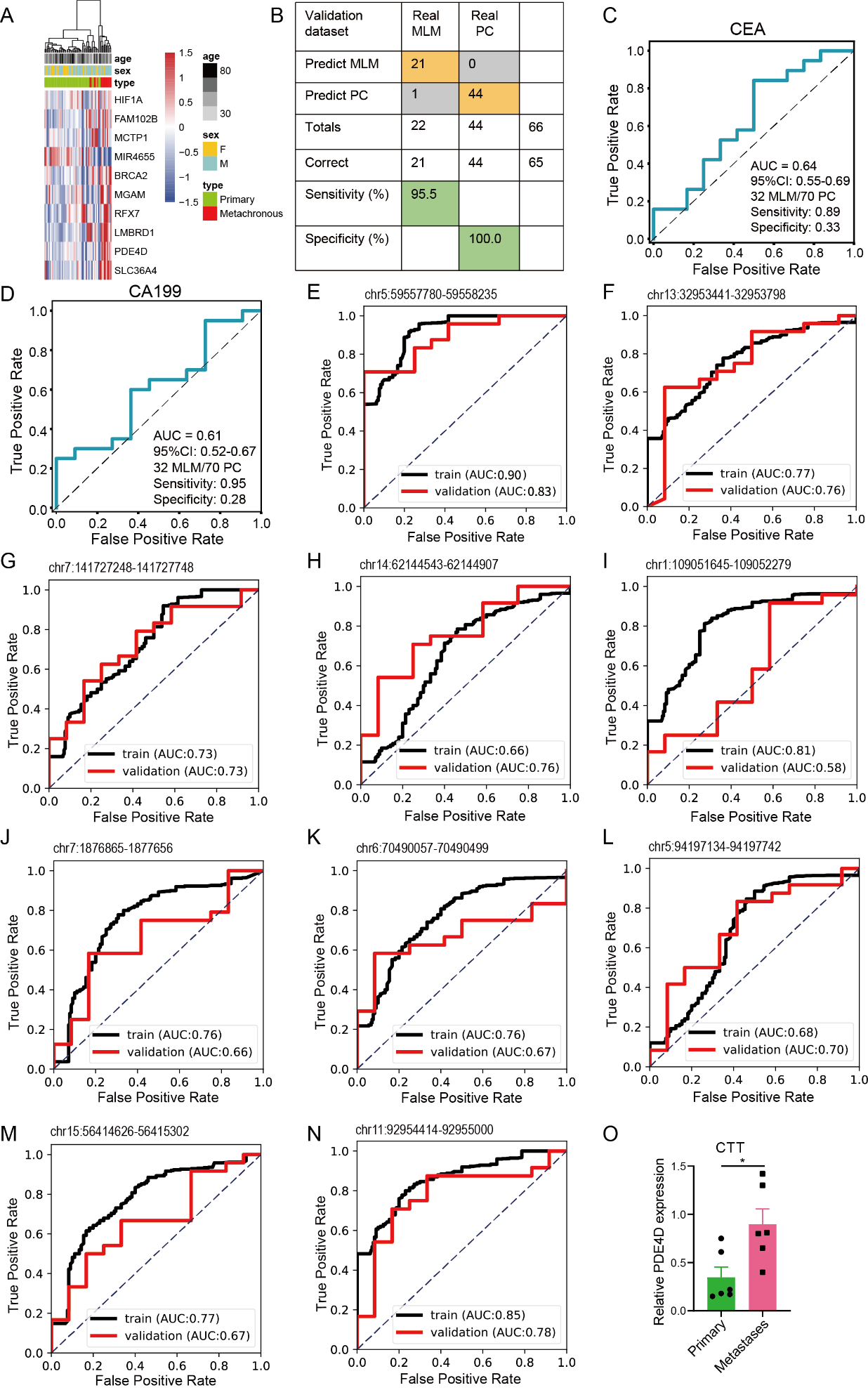
**

**Supplementary Fig. 5. The individual prediction performance of each marker in the ten markers in the training and validation cohort.** (A) Heatmaps of 10 5hmC markers with PC and MLM patients in the training cohort, sex and age information labeled in the figure. (B) Confusion matrices built from the prediction model prediction in the training cohort. (C) Receiver operating characteristic (ROC) curve of the classification model with CEA in portal vein blood samples. (D) ROC) curve of the classification model with CA199 in portal vein blood samples. (E to N) Individual ROC curves for each marker in the training cohort and validation cohort. (O) Quantitative comparison of *PDE4D* RNA level in PC patients and MLM patients. * p < 0.05.

**
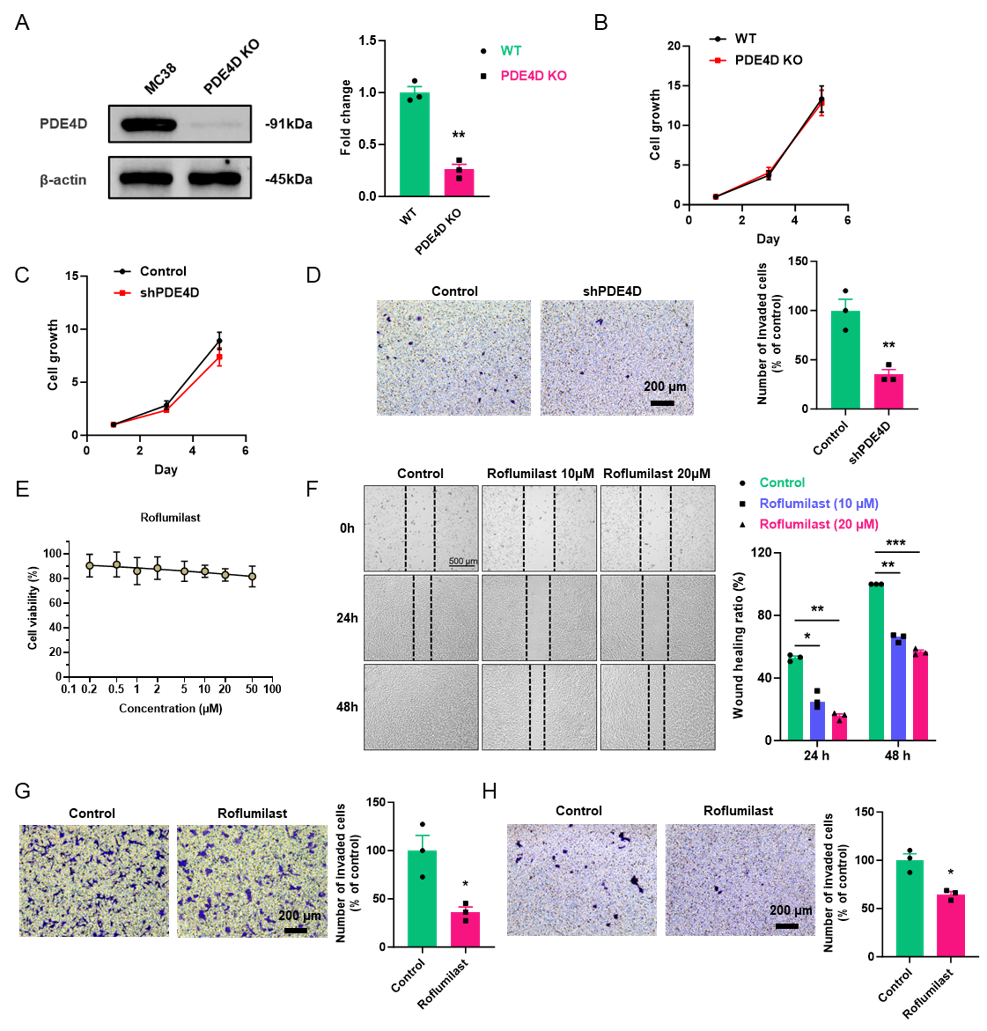
**

**Supplementary Fig. 6. PDE4D suppression inhibits migration in multiple CRC cells.** (A) Western blot analysis and quantitatively statistic of PDE4D expression in PDE4D KO cells. (n=3) (B) The proliferation curve of PDE4D KO cell. (C) The proliferation curve of shPDE4D SW480 cell. (D) The migration of shPDE4D SW480 cells was assessed using a transwell migration assays. (n=3) Scale bar, 200 µm. (E) Cell viability of MC38 cells treated with roflumilast. (F) Scratch assay analysis of MC38 cells treated with roflumilast. Images were acquired at 0, 24 and 48 hours. (n=3) Scale bar, 500 µm. (G to H) The migration of LoVo cells (G) and HCT116 cells (H) was assessed using a transwell migration assays. Right, Representative images of the assay. (n=3) Scale bar, 200 µm. Data are shown as means ± SEM. * p < 0.05; ** p < 0.01; *** p < 0.001.


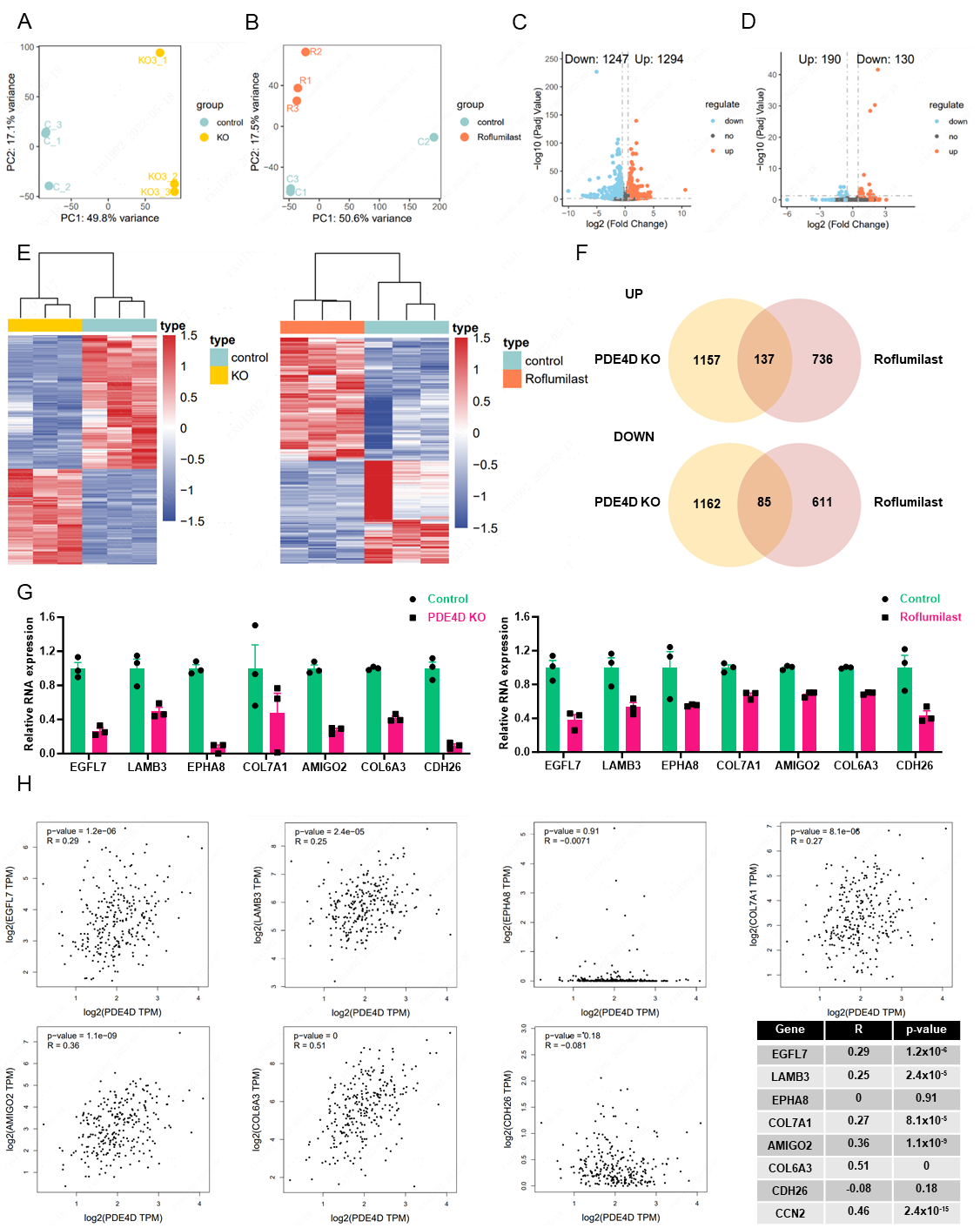


**Supplementary Fig. 7. Association between CCN2 and PDE4D according to RNA-seq data.** (A to B) Principal component analysis (PCA) of PDE4D KO cells compared with wild type cells (A) and roflumilast treated cells compared with untreated cells (B). (C to D) Volcano plot of PDE4D KO cells (C) and roflumilast treated cells (D). Significantly alterations (log2FoldChange >0.5, p-value < 0.05). (E) Gene expressions in PDE4D KO MC38 cells compared with wild type cells, as well as roflumilast treated cells compared with untreated cells analyzed by RNA-seq. (F) Venn diagram indicating overlap and specifity of upregulations and downregulations from RNA-seq data. (G) Expression of genes accumulated in cell adhesion pathway in PDE4D KO cells and roflumilast treated cells. (n=3) (H) Correlation plots of the mRNA expression of PDE4D with the mRNA expressions of genes in the cell adhesion pathway, including LAMB3, EGFL7, EPHA8, COL7A1, AMIGO2, COL6A3 and CDH26 in CRC sequencing data from TCGA dataset. Data are shown as means ± SEM.

**
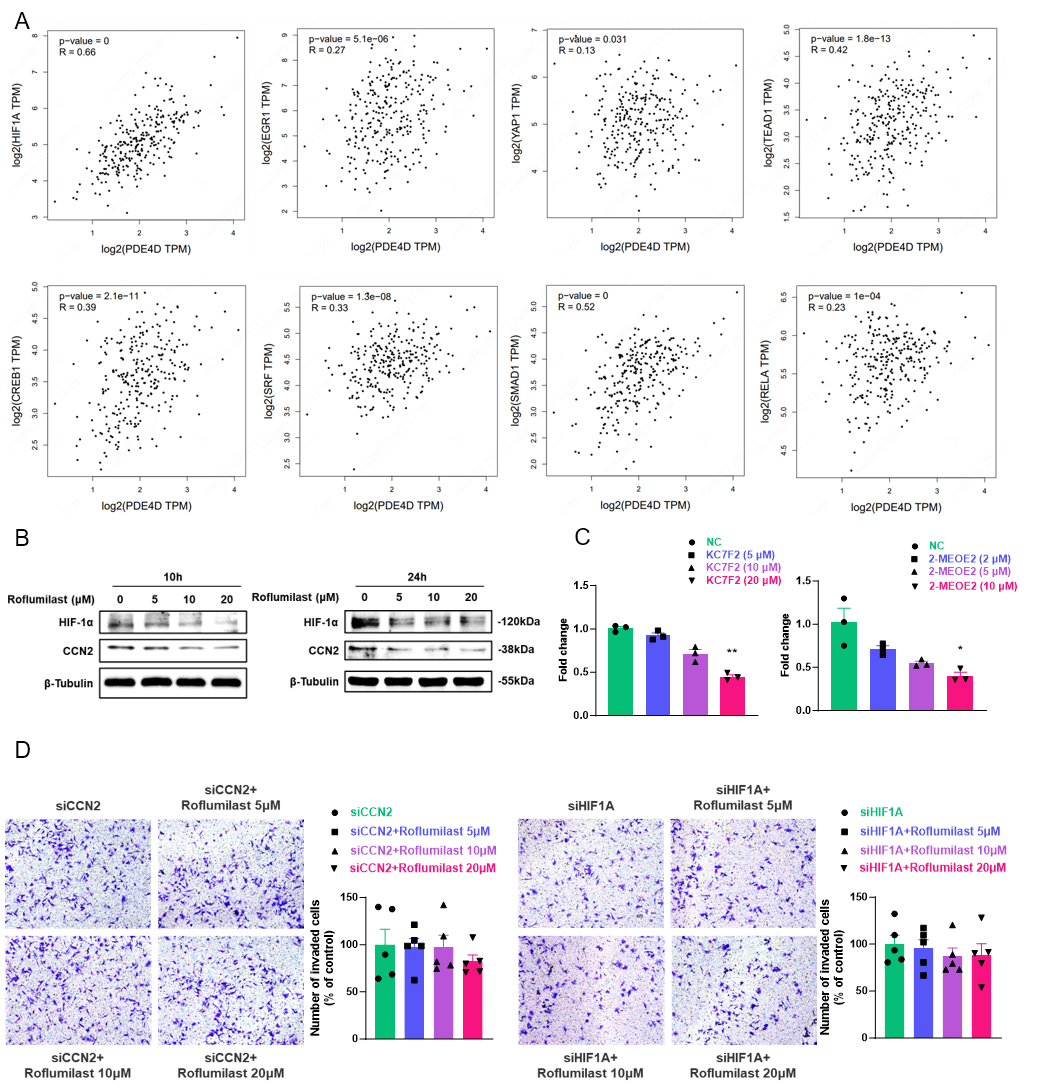
**

**Supplementary Fig. 8. PDE4 suppression inhibits HIF-1α and CCN2.** (A) Correlation plots of the mRNA level of PDE4D with the mRNA level of transcription factors, including HIF1A, EGR1, YAP1, TEAD1, CREB1, SRF, SMAD1 and RELA in CRC sequencing data from TCGA dataset. (B) Western blot for HIF-1α and CCN2 in HCT116 cells treated with roflumilast for 10h and 24h. (C) RNA expression level of CCN2 in MC38 cells treated with different concentration of KC7F2 and 2MEOE2. (n=3) (D to E) The migration of MC38 cells treated with siCCN2 (D)/ siHIF1A (E) and roflumilast was assessed using a transwell migration assays. Right, Representative images of the assay. (n=4) Scale bar, 200 µm. Data are shown as means ± SEM. * p < 0.05; ** p < 0.01.


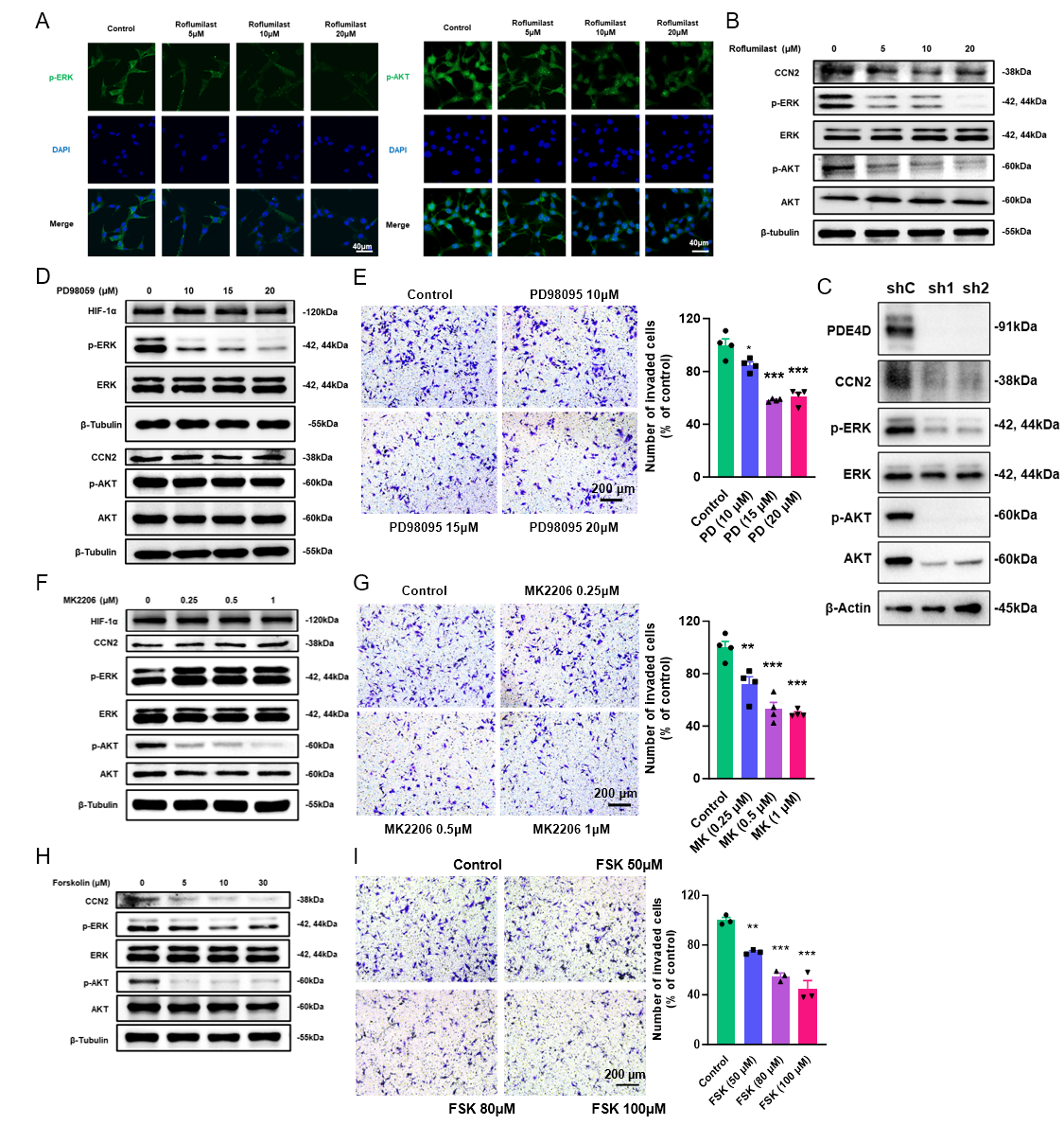


**Supplementary Fig. 9.** **PDE4D** **blocking inhibits expression of CCN2 and phosphorylation of AKT and ERK.** (A) Immunofluorescence images for phospho-ERK and phospho-AKT in MC38 cells treated with roflumilast. (B) Western blot analysis for the expression of CCN2, phospho-AKT, total AKT, phospho-ERK and total ERK in roflumilast treated MC38 cells. (C) Proteins expression level after PDE4D knockdown in SW480 cells. (n=2) (D) Effect of PD98059 mediated inhibiting of p-ERK after treating for 24 hours. (n=2) (E) Transwell migration assays of MC38 cells treated with PD98059. (n=4) (F) Effect of MK2206 mediated inhibiting of p-ERK after treating for 24 hours. (n=2) (G) Effect of MK2206 mediated inhibiting of p-AKT after treating for 24 hours. (n=4) (H) Western blot for CCN2, phospho-AKT, total AKT, phospho-ERK and total ERK in MC38 cells treated with FSK. (n=2) (I) Transwell migration assays of MC38 cells treated with FSK. Data are shown as means ± SEM. * p < 0.05; ** p < 0.01; *** p < 0.001.

**
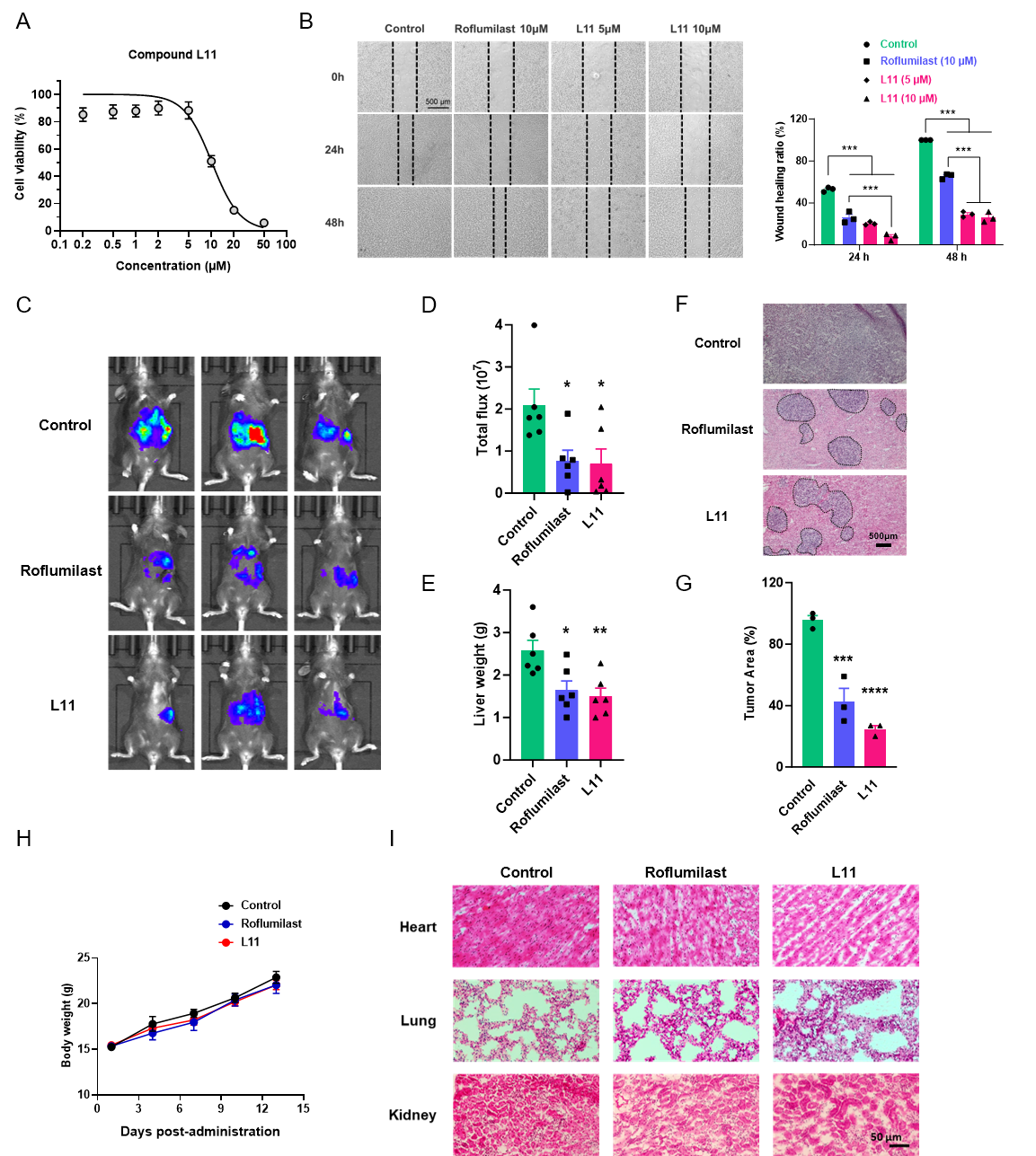
**

**Supplementary Fig. 10.** **Metastases inhibition and safety tests of compound L11.** (A) Cell viability inhibition of MC38 cells by compound L11. (B) Scratch assay analysis of MC38 cells treated with PBS, roflumilast and compound L11. Images were acquired at 0, 24 and 48 hours. Right, quantitative analysis of scratch wound closure. (n=3) Scale bar, 500 µm. (C) Bioluminescence images of liver metastases mice treated with roflumilast or compound L11. (D) Quantitative analysis of fluorescence intensity of mice. (n=6) (E) Statistical data of liver weight After dissecting three groups of mice. (n=6) (F) Histopathological examination of liver tissue sections. (G) Quantification of liver metastases area in H&E sections. (n=3) (H) Body weight curve of mice treated with PBS, roflumilast and compound L11. (I) H&E staining results of main organs of mice, including heart, lung and kidney. Scale bar, 50 µm. Data are shown as means ± SEM. * p < 0.05; ** p < 0.01; *** p < 0.001.


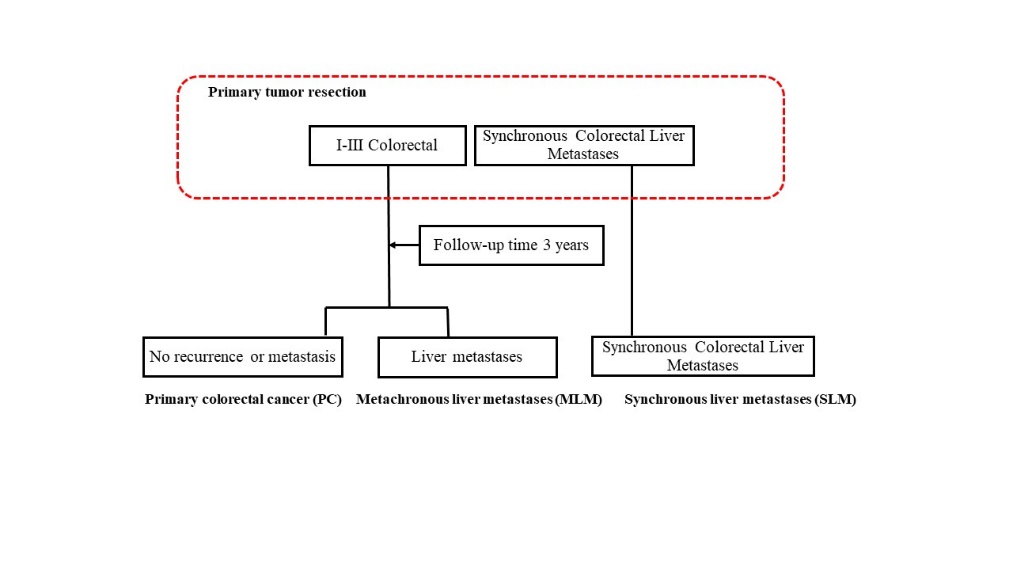


**Supplementary Scheme 1. Diagnosis and follow-up of CRC patients.**

**Supplementary Scheme 2. Synthesis of compounds L1‒L9.** Reagents and conditions: (a) NaBH_4_, MeOH, rt, 1h; (b) (i) n-BuLi,THF,-78℃; (ii) Trisopropylbrate, -78℃, 30min, -30℃, 2h; (iii) 2M HCl, H_2_O, pH=5; (c) Pd(OAc)_2_, Xantphos, Cs_2_CO_3_, Ph_3_Me, 115℃, 24h; (d) Pd(OAc)_2_, K_2_CO_3_, H_2_O:DMAC=1:6, 80℃, 2h; (e) KOH, t-BuOH, H_2_O, 60℃, 4h.

**Supplementary Scheme 3. Synthesis of compounds L10‒L13.** Reagents and conditions: (a) NaBH_4_, MeOH, rt, 1h; (b) (i) n-BuLi,THF,-78℃; (ii) Trisopropylbrate, -78℃, 30min, -30℃, 2h; (iii) 2M HCl, H_2_O, pH=5; (c) Haloalkanes, NaOH, EtOH, rt to 85℃, 24h; (d) Br_2_, DCM, -60℃ to -5℃, 2h; (e) diethyl (bromodifluoromethyl)phosphonate, KOH, CH_3_CN:H_2_O=1:1, rt, 15 min; (f) Pd(OAc)_2_, K_2_CO_3_, H_2_O:DMAC=1:6, 80℃, 2h.

**Supplementary** **Table 1. Statistical characteristics of baseline indicators in CRC patients.**

| Characteristics | Total (n=133) | Primary (n=70) | Metachronous (n=32) | Simultaneous (n=31) |
| --- | --- | --- | --- | --- |
| Age (years)  <60/≥60 | 52/81 | 25/45 | 11/21 | 16/15 |
| Gender  Female/Male | 45/88 | 29/41 | 7/25 | 9/22 |
| Tumor location  Colon/Rectum | 74/59 | 40/30 | 17/15 | 17/14 |
| Tumor size (cm^3^)  <10/≥10 | 36/97 | 18/52 | 6/26 | 12/19 |
| Histology (differentiation)  Well/Moderate/Poor | 4/101/21 | 4/51/10 | 0/27/4 | 0/23/7 |
| Lymphovascular invasion  Negative/Positive | 77/56 | 54/16 | 10/22 | 13/18 |
| Adjuvant chemotherapy | 58/102 | 34/70 | 24/32 | - |
| Stage Ⅰ/Ⅱ/Ⅲ/Ⅳ | 15/40/47/31 | 14/32/24/0 | 1/8/23/0 | 0/0/0/31 |
| HER2 0/1+/2+/3+ | 68/20/13/2 | 33/10/8/1 | 15/6/2/1 | 20/4/3/0 |
| Serum CEA (ng/mL)  <5/≥5 | 70/63 | 50/20 | 11/21 | 9/22 |
| Serum CA199 (U/mL)  <37/≥37 | 96/37 | 59/11 | 24/8 | 13/18 |

Abbreviations

HER2: human epidermal growth factor receptor-2; CEA: carcino-embryonic antigen; CA199: carbohydrate antigen199.

**Supplementary Table 2. Coefficients for 10 DhMRs in the logistic regression model trained by the training cohort**

|  | Coefficients | SE | Z | P>\|Z\| | 97.5% CI | GeneID |
| --- | --- | --- | --- | --- | --- | --- |
| Intercept | 19.6651 | 1.971 | -0.011 | 0.009 | [-7.551, -0.785] |  |
| chr7_141727248_141727748 | 1.0121 | 0.418 | 0.004 | 0.097 | [-5.478, 7.503] | MGAM |
| chr7_1876865_1877656 | -1.5569 | 0.491 | -0.003 | 0.027 | [-6.063, 2.949] | MIR4655 |
| chr11_92954414_92955000 | 2.3106 | 0.913 | 0.004 | 0.034 | [-9.736, 4.357] | SLC36A4 |
| chr5_59557780_59558235 | 1.1234 | 0.479 | 0.003 | 0.016 | [-1.683, 3.930] | PDE4D |
| chr5_94197134_94197742 | 0.4479 | 0.973 | 0.002 | 0.002 | [-3.652, 4.548] | MCTP1 |
| chr13_32953441_32953798 | -10.4726 | 1.577 | -0.031 | 0.064 | [-9.952, 9.007] | BRCA2 |
| chr6_70490057_70490499 | -3.8177 | 0.192 | -0.009 | 0.052 | [-6.378, 8.743] | LMBRD1 |
| chr15_56414626_56415302 | -4.9716 | 1.273 | -0.011 | 0.019 | [-1.210, 1.267] | RFX7 |
| chr1_109051645_109052279 | 2.4804 | 0.768 | 0.005 | 0.047 | [ -3.528, 8.489] | FAM102B |
| chr14_62144543_62144907 | 0.7744 | 0.657 | 0.002 | 0.098 | [-0.2215, 1.770] | HIF1A |

**Abbreviation:** **DhMRs**, Differentially 5hMc-enriched Regions. **SE**, standard errors of coefficients;

**z value**, Wald z-statistic value; **CI**, confidence interval.

**Supplementary Table 3. Inhibitory Activities of 2-arylbenzofurans towards PDE4.**

| **Compounds** | **Structure** | **PDE4 inhibition** | | **IC_50_** (nM) |
| --- | --- | --- | --- | --- |
|  |  | **1μM** | **100 nM** |  |
| **L1** |  | 55.71% | 41.94% | – |
| **L2** |  | 68.54% | 48.62% | 181±13 |
| **L3** |  | 24.14% | 17.04% | – |
| **L4** |  | 59.52% | 28.87% | – |
| **L5** |  | 36.91% | 15.91% | – |
| **L6** |  | 55.49% | 30.29% | – |
| **L7** |  | 77.50% | 44.08% | 197±37.4 |
| **L8** |  | 61.62% | 30.59% | – |
| **L9** |  | 65.85% | 39.13% | – |
| **L10** |  | 37.40% | – | – |
| **L11** |  | 83.55% | 33.05% | 54±15.5 |
| **L12** |  | **73.33%** | 13.18% | 910 |
| **L13** |  | 92.25% | 42.73% | 145.1±2.3 |
| **Rolipram** |  | 65.06%@ 800 nM | 43.30%@ 400 nM | 590±50 |
| **Roflumilast** |  | – | 60.27% | 0.10±0.01 |

**Supplementary Table 4. Selectivity Index of L11 across PDE Families. `**

| **Proteins** | **IC_50_ (nM)** | **Selectivity index** |
| --- | --- | --- |
| PDE4D2 (86-413) | 54.0±5.5 | – |
| PDE1B (10-487) | >10,000 | >185 |
| PDE2A (580-919) | 4,840±90 | 90 |
| PDE3A (679-1087) | >10,000 | >185 |
| PDE5A1 (535-860) | 3,000±350 | 56 |
| PDE7A1 (130-482) | 2,290±180 | 42 |
| PDE8A1 (480-820) | 3,610±320 | 67 |
| PDE9A2 (181-506) | >10,000 | >185 |
| PDE10A (449-770) | 6,920±10 | 116 |
| PDE11A(1-934) | 4,010±320 | 74 |

**Supplementary Table 5. Statistics on diffraction data and structure refinement of PDE4D-L11.**

| **Data collection** | |
| --- | --- |
| Wavelength (Å) | 1.54056 |
| Temperature (K) | 100 |
| Resolution (Å) | 2.10 |
| Space group | P2_1_2_1_2_1_ |
| a, b, c (Å) | 58.36, 80.43, 164.58 |
| α, β, γ (°) | 90.0, 90.0, 90.0 |
| No. reflections | 45739 (4515 ^a^) |
| Completeness (%) | 99.77 (99.82 ^a^) |
| R_merge_ | 0.179 (0.272 ^a^) |
| <I/σ(I) > | 19.0 (4.2 ^a^) |
| Redundancy | 4.0 (5.7 ^a^) |
| **Structure reﬁnement** | |
| R-factor/R-free | 0.227/0.278 |
| Bond lengths | 0.008 Å |
| Bond angles | 0.912 |
| Protein | 22.58 (5208) |
| Inhibitor | 18.32 (50) |
| Zn^2+^ | 18.37 (2) |
| Mg^2+^ | 12.24 (2) |
| Waters | 25.60 (288) |
| Preferred | 96.88% |
| Allowed | 3.13% |

*^a^* The highest resolution shell of 2.17 – 2.10 Å.

**Supplementary Table 6. Emetic Assays of L11 and Roflumilast on Beagle Dogs.**

| **Compounds** | **Dose (mg/kg, po)** | **No. of animals with emesis/No. of test animals** |
| --- | --- | --- |
| **L11** | 10 | 0/3 |
| **Roflumilast** | 1.0 | 3/3 |

**Supplementary Table 7. Effects on food intake in rats after 2 weeks administered of L11 and 2 weeks of recovery (g/rat·d)**

| Weeks | Groups | | | | |
| --- | --- | --- | --- | --- | --- |
|  | Gender | n | Control | **L11**(low-dose group) | **L11** (high-dose group) |
| 1 week | ♀ | 10 | 16.5 | 15.2 | 17.1 |
| 2 week | ♀ | 10 | 16.6 | 15.9 | 18.3 |
| 3 week | ♀ | 5 | 18.9 | 18.9 | 17.9 |
| 4 week | ♀ | 5 | 19.6 | 21.2 | 20.7 |
| 1 week | ♂ | 10 | 21.6 | 22.1 | 23.1 |
| 2 week | ♂ | 10 | 20.2 | 19.9 | 21.9 |
| 3 week | ♂ | 5 | 23.6 | 23.8 | 22.5 |
| 4 week | ♂ | 5 | 26.3 | 26.5 | 26.0 |

**Supplementary Table 8. Effects on hematology indicators in rats After 2 weeks administered of L11 (♀)**

| Items | | Control | | | **L11**(low-dose group) | | | **L11** (high-dose group) | | |
| --- | --- | --- | --- | --- | --- | --- | --- | --- | --- | --- |
| n= |  | 5 | | | 5 | | | 4 | | |
| WBC | (109/L) | 2.97 | ± | 1.57 | 2.29 | ± | 1.46 | 3.25 | ± | 1.87 |
| RBC | (1012/L) | 7.03 | ± | 0.60 | 6.95 | ± | 0.11 | 7.10 | ± | 0.54 |
| HGB | (g/L) | 130 | ± | 5 | 128 | ± | 7 | 134 | ± | 11* |
| HCT | (%) | 40.6 | ± | 2.0 | 39.1 | ± | 2.4 | 39.5 | ± | 2.5 |
| MCV | ( fL) | 58.0 | ± | 4.0 | 56.3 | ± | 2.8 | 55.7 | ± | 0.9 |
| MCH | (pg) | 18.5 | ± | 1.4 | 18.5 | ± | 0.8 | 18.9 | ± | 0.5 |
| MCHC | (g/L) | 319 | ± | 4 | 328 | ± | 3** | 339 | ± | 8** |
| PLT | (109/L) | 1090 | ± | 102 | 1204 | ± | 134 | 836 | ± | 548 |
| RDW-SD | ( fL) | 24.9 | ± | 1.3 | 23.9 | ± | 1.8 | 21.9 | ± | 0.9* |
| RDW-CV | (%) | 12.0 | ± | 1.2 | 11.6 | ± | 0.4 | 11.0 | ± | 0.6 |
| PDW | (fL) | 7.3 | ± | 0.3 | 7.4 | ± | 0.1 | 7.4 | ± | 0.5 |
| MPV | ( fL ) | 7.2 | ± | 0.2 | 7.4 | ± | 0.1 | 7.2 | ± | 0.3 |
| P-LCR | (%) | 5.4 | ± | 1.0 | 6.2 | ± | 0.5 | 5.5 | ± | 1.9 |
| PCT | (%) | 0.66 | ± | 0.07 | 0.70 | ± | 0.15 | 0.56 | ± | 0.37 |
| NEUT | (109/L) | 0.43 | ± | 0.17 | 0.43 | ± | 0.35 | 0.37 | ± | 0.18 |
| LYMPH | (109/L) | 2.36 | ± | 1.28 | 1.71 | ± | 1.04 | 2.71 | ± | 1.60 |
| MONO | (109/L) | 0.14 | ± | 0.09 | 0.10 | ± | 0.07 | 0.15 | ± | 0.09 |
| EO | (10^9^/L) | 0.04 | ± | 0.03 | 0.04 | ± | 0.03 | 0.03 | ± | 0.01 |
| BASO | (10^9^/L) | 0.01 | ± | 0.01 | 0.01 | ± | 0.01 | 0.01 | ± | 0.01 |
| NEUT | (%) | 15.0 | ± | 2.1 | 17.0 | ± | 5.6 | 11.6 | ± | 1.0* |
| LYMPH | (%) | 79.1 | ± | 1.5 | 76.2 | ± | 5.8 | 82.9 | ± | 1.7** |
| MONO | (%) | 4.4 | ± | 0.7 | 4.7 | ± | 1.5 | 4.3 | ± | 1.3 |
| EO | (%) | 1.3 | ± | 0.5 | 1.8 | ± | 1.1 | 0.9 | ± | 0.3 |
| BASO | (%) | 0.1 | ± | 0.2 | 0.3 | ± | 0.2 | 0.3 | ± | 0.2 |
| RET | (%) | 3.30 | ± | 0.93 | 3.63 | ± | 1.02 | 3.44 | ± | 0.78 |
| RET | (10^9^/L) | 230 | ± | 62 | 252 | ± | 72 | 244 | ± | 57 |
| IRF | (%) | 58.7 | ± | 7.5 | 60.0 | ± | 7.2 | 56.2 | ± | 8.2 |
| LFR | (%) | 41.3 | ± | 7.5 | 40.0 | ± | 7.2 | 43.8 | ± | 8.2 |
| MFR | (%) | 17.4 | ± | 1.0 | 19.1 | ± | 1.4 | 22.2 | ± | 2.6** |
| HFR | (%) | 41.2 | ± | 7.5 | 40.8 | ± | 7.7 | 34.0 | ± | 10.5 |

**Supplementary Table 9. Effects on hematology indicators in rats After 2 weeks administered of L11 (♂)**

| Items | | Control | | | **L11**(low-dose group) | | | **L11** (high-dose group) | | |
| --- | --- | --- | --- | --- | --- | --- | --- | --- | --- | --- |
| n= |  | 5 | | | 4 | | | 5 | | |
| WBC | (10^9^/L) | 4.99 | ± | 2.65 | 7.31 | ± | 0.96 | 6.56 | ± | 4.17 |
| RBC | (10^12^/L) | 7.30 | ± | 0.24 | 7.17 | ± | 0.22 | 7.09 | ± | 0.39 |
| HGB | (g/L) | 144 | ± | 5 | 144 | ± | 3 | 140 | ± | 7 |
| HCT | (%) | 43.7 | ± | 1.5 | 42.7 | ± | 0.9 | 41.1 | ± | 2.3 |
| MCV | ( fL) | 59.9 | ± | 1.4 | 59.6 | ± | 2.4 | 58.1 | ± | 4.2 |
| MCH | (pg) | 19.7 | ± | 0.5 | 20.0 | ± | 0.7 | 19.8 | ± | 1.3 |
| MCHC | (g/L) | 329 | ± | 7 | 336 | ± | 1 | 341 | ± | 4* |
| PLT | (10^9^/L) | 1126 | ± | 669 | 1383 | ± | 285 | 1109 | ± | 622 |
| RDW-SD | ( fL) | 26.7 | ± | 2.8 | 25.8 | ± | 1.0 | 25.5 | ± | 2.4 |
| RDW-CV | (%) | 12.2 | ± | 1.2 | 12.1 | ± | 0.4 | 12.1 | ± | 0.4 |
| PDW | (fL) | 7.9 | ± | 0.5 | 7.8 | ± | 0.5 | 7.3 | ± | 0.3 |
| MPV | ( fL ) | 7.7 | ± | 0.5 | 7.5 | ± | 0.2 | 7.4 | ± | 0.5 |
| P-LCR | (%) | 8.4 | ± | 3.7 | 6.8 | ± | 1.5 | 7.9 | ± | 5.7 |
| PCT | (%) | 0.62 | ± | 0.34 | 0.82 | ± | 0.11 | 0.69 | ± | 0.37 |
| NEUT | (10^9^/L) | 0.91 | ± | 0.54 | 0.93 | ± | 0.23 | 0.81 | ± | 0.72 |
| LYMPH | (10^9^/L) | 3.80 | ± | 2.11 | 5.94 | ± | 0.69 | 5.39 | ± | 3.23 |
| MONO | (10^9^/L) | 0.21 | ± | 0.08 | 0.37 | ± | 0.09* | 0.30 | ± | 0.22 |
| EO | (10^9^/L) | 0.05 | ± | 0.02 | 0.06 | ± | 0.02 | 0.03 | ± | 0.02 |
| BASO | (10^9^/L) | 0.02 | ± | 0.01 | 0.02 | ± | 0.01 | 0.02 | ± | 0.02 |
| NEUT | (%) | 18.1 | ± | 4.8 | 12.6 | ± | 1.7 | 10.7 | ± | 3.4* |
| LYMPH | (%) | 76.0 | ± | 5.7 | 81.4 | ± | 2.2 | 84.4 | ± | 4.4* |
| MONO | (%) | 4.6 | ± | 1.4 | 5.0 | ± | 1.0 | 4.2 | ± | 1.6 |
| EO | (%) | 1.0 | ± | 0.1 | 0.8 | ± | 0.2 | 0.5 | ± | 0.1** |
| BASO | (%) | 0.3 | ± | 0.1 | 0.3 | ± | 0.1 | 0.2 | ± | 0.2 |
| RET | (%) | 4.94 | ± | 1.22 | 4.71 | ± | 0.77 | 4.83 | ± | 0.82 |
| RET | (10^9^/L) | 360 | ± | 83 | 336 | ± | 45 | 341 | ± | 51 |
| IRF | (%) | 61.0 | ± | 4.0 | 60.0 | ± | 3.3 | 59.5 | ± | 4.9 |
| LFR | (%) | 39.0 | ± | 4.0 | 40.1 | ± | 3.3 | 40.5 | ± | 4.9 |
| MFR | (%) | 16.2 | ± | 1.3 | 19.4 | ± | 2.0* | 20.8 | ± | 2.9* |
| HFR | (%) | 44.8 | ± | 5.0 | 40.6 | ± | 4.6 | 38.7 | ± | 7.5 |

**Supplementary Table 10.** Effects on hematology indicators in rats after 2 weeks administered of **L11** and 2 weeks of recovery (♀)

| Items | | Control | | | **L11**(low-dose group) | | | **L11** (high-dose group) | | |
| --- | --- | --- | --- | --- | --- | --- | --- | --- | --- | --- |
| n= |  | 5 | | | 4 | | | 5 | | |
| WBC | (10^9^/L) | 4.94 | ± | 3.30 | 4.30 | ± | 0.98 | 4.10 | ± | 2.66 |
| RBC | (10^12^/L) | 7.32 | ± | 0.40 | 7.04 | ± | 0.28 | 6.98 | ± | 0.24 |
| HGB | (g/L) | 138 | ± | 5 | 135 | ± | 8 | 132.40 | ± | 1.95 |
| HCT | (%) | 40.32 | ± | 2.10 | 40.63 | ± | 1.85 | 39.50 | ± | 0.22 |
| MCV | ( fL) | 55.22 | ± | 3.55 | 57.75 | ± | 1.28 | 56.64 | ± | 1.72 |
| MCH | (pg) | 18.94 | ± | 1.06 | 19.10 | ± | 0.71 | 19.02 | ± | 0.59 |
| MCHC | (g/L) | 343.60 | ± | 10.88 | 331 | ± | 6 | 335.20 | ± | 3.83 |
| PLT | (10^9^/L) | 1329 | ± | 245 | 1068 | ± | 165 | 1134 | ± | 96 |
| RDW-SD | ( fL) | 21.98 | ± | 1.89 | 24.98 | ± | 1.02 | 23.84 | ± | 1.25 |
| RDW-CV | (%) | 11.24 | ± | 1.14 | 12.03 | ± | 0.97 | 11.60 | ± | 0.89 |
| PDW | (fL) | 7.46 | ± | 0.42 | 7.60 | ± | 0.46 | 7.32 | ± | 0.19 |
| MPV | ( fL ) | 7.26 | ± | 0.25 | 7.58 | ± | 0.22 | 7.28 | ± | 0.13 |
| P-LCR | (%) | 5.92 | ± | 1.21 | 7.60 | ± | 1.01 | 5.54 | ± | 0.91 |
| PCT | (%) | 0.81 | ± | 0.12 | 0.69 | ± | 0.03 | 0.69 | ± | 0.06 |
| NEUT | (10^9^/L) | 0.65 | ± | 0.30 | 0.60 | ± | 0.22 | 0.71 | ± | 0.47 |
| LYMPH | (10^9^/L) | 3.99 | ± | 2.96 | 3.42 | ± | 0.87 | 3.20 | ± | 2.10 |
| MONO | (10^9^/L) | 0.23 | ± | 0.13 | 0.23 | ± | 0.08 | 0.13 | ± | 0.10 |
| EO | (10^9^/L) | 0.05 | ± | 0.03 | 0.05 | ± | 0.02 | 0.04 | ± | 0.03 |
| BASO | (10^9^/L) | 0.01 | ± | 0.01 | 0.01 | ± | 0.01 | 0.01 | ± | 0.01 |
| NEUT | (%) | 15.06 | ± | 4.81 | 14.18 | ± | 4.59 | 18.32 | ± | 4.82 |
| LYMPH | (%) | 78.52 | ± | 6.54 | 79.28 | ± | 5.30 | 77.52 | ± | 4.89 |
| MONO | (%) | 5.06 | ± | 1.61 | 5.23 | ± | 0.81 | 3.00 | ± | 0.56* |
| EO | (%) | 1.12 | ± | 0.96 | 1.03 | ± | 0.44 | 0.90 | ± | 0.57 |
| BASO | (%) | 0.24 | ± | 0.25 | 0.30 | ± | 0.08 | 0.26 | ± | 0.19 |
| RET | (%) | 3.34 | ± | 0.33 | 3.12 | ± | 0.77 | 4.13 | ± | 0.74 |
| RET | (10^9^/L) | 243.70 | ± | 22.51 | 218.10 | ± | 44.84 | 287.38 | ± | 46.14 |
| IRF | (%) | 55.76 | ± | 8.26 | 50.40 | ± | 8.71 | 63.56 | ± | 2.34 |
| LFR | (%) | 44.24 | ± | 8.26 | 49.60 | ± | 8.71 | 36.44 | ± | 2.34 |
| MFR | (%) | 17.38 | ± | 1.45 | 15.20 | ± | 1.30 | 15.18 | ± | 1.03* |
| HFR | (%) | 38.38 | ± | 9.17 | 35.20 | ± | 9.26 | 48.38 | ± | 3.11* |

*p<0.05, **p<0.01, compared to the control group.

**Supplementary Table 11.** Effects on hematology indicators in rats after 2 weeks administered of **L11** and 2 weeks of recovery (♂)

| Items | | Control | | | **L11**(low-dose group) | | | **L11** (high-dose group) | | |
| --- | --- | --- | --- | --- | --- | --- | --- | --- | --- | --- |
| n= |  | 5 | | | 3 | | | 5 | | |
| WBC | (10^9^/L) | 5.76 | ± | 2.99 | 3.45 | ± | 1.90 | 5.12 | ± | 1.70 |
| RBC | (10^12^/L) | 7.38 | ± | 0.56 | 7.69 | ± | 0.20 | 8.29 | ± | 0.40* |
| HGB | (g/L) | 138 | ± | 7 | 131 | ± | 3 | 150 | ± | 1** |
| HCT | (%) | 41.34 | ± | 2.07 | 39.83 | ± | 1.19 | 44.20 | ± | 0.82* |
| MCV | ( fL) | 56.12 | ± | 2.93 | 51.80 | ± | 0.26* | 53.40 | ± | 2.05 |
| MCH | (pg) | 18.70 | ± | 1.13 | 17.03 | ± | 0.12* | 18.10 | ± | 0.76 |
| MCHC | (g/L) | 332.80 | ± | 3.96 | 328.67 | ± | 3.06 | 339.00 | ± | 6.20 |
| PLT | (10^9^/L) | 1212.40 | ± | 107.61 | 1147.00 | ± | 86.76 | 1195.80 | ± | 67.33 |
| RDW-SD | ( fL) | 23.06 | ± | 1.12 | 23.80 | ± | 0.92 | 22.52 | ± | 0.95 |
| RDW-CV | (%) | 11.76 | ± | 1.04 | 13.93 | ± | 0.75* | 13.04 | ± | 1.06 |
| PDW | (fL) | 7.50 | ± | 0.46 | 7.43 | ± | 0.21 | 7.22 | ± | 0.19 |
| MPV | ( fL ) | 7.34 | ± | 0.26 | 7.30 | ± | 0.17 | 7.06 | ± | 0.18 |
| P-LCR | (%) | 6.04 | ± | 1.51 | 5.73 | ± | 0.99 | 4.90 | ± | 0.87 |
| PCT | (%) | 0.78 | ± | 0.11 | 0.74 | ± | 0.09 | 0.69 | ± | 0.03 |
| NEUT | (10^9^/L) | 1.00 | ± | 0.45 | 1.00 | ± | 0.89 | 0.66 | ± | 0.34 |
| LYMPH | (10^9^/L) | 4.46 | ± | 2.39 | 2.23 | ± | 0.94 | 4.18 | ± | 1.54 |
| MONO | (10^9^/L) | 0.23 | ± | 0.14 | 0.16 | ± | 0.08 | 0.22 | ± | 0.07 |
| EO | (10^9^/L) | 0.06 | ± | 0.03 | 0.05 | ± | 0.01 | 0.04 | ± | 0.01 |
| BASO | (10^9^/L) | 0.01 | ± | 0.01 | 0.01 | ± | 0.01 | 0.02 | ± | 0.01 |
| NEUT | (%) | 18.08 | ± | 1.92 | 25.97 | ± | 9.04 | 13.22 | ± | 6.12 |
| LYMPH | (%) | 76.82 | ± | 2.41 | 67.50 | ± | 8.41 | 81.12 | ± | 7.42 |
| MONO | (%) | 3.80 | ± | 1.30 | 4.57 | ± | 0.46 | 4.40 | ± | 1.52 |
| EO | (%) | 1.08 | ± | 0.33 | 1.57 | ± | 0.57 | 0.92 | ± | 0.51 |
| BASO | (%) | 0.22 | ± | 0.16 | 0.40 | ± | 0.10 | 0.34 | ± | 0.15 |
| RET | (%) | 3.63 | ± | 0.49 | 3.30 | ± | 0.37 | 3.47 | ± | 0.47 |
| RET | (10^9^/L) | 267.38 | ± | 37.29 | 254.03 | ± | 33.36 | 286.24 | ± | 29.45 |
| IRF | (%) | 57.60 | ± | 2.75 | 59.93 | ± | 2.06 | 55.10 | ± | 3.88 |
| LFR | (%) | 42.40 | ± | 2.75 | 40.07 | ± | 2.06 | 44.90 | ± | 3.88 |
| MFR | (%) | 16.00 | ± | 0.71 | 17.10 | ± | 2.01 | 18.14 | ± | 1.42 |
| HFR | (%) | 41.60 | ± | 3.22 | 42.83 | ± | 2.57 | 36.96 | ± | 4.31 |

*p<0.05, **p<0.01, compared to the control group.

**Supplementary Table 12.** Effects on serum test indicators in rats after 2 weeks administered of **L11**

| Items | | Control | | | **L11**(low-dose group) | | | **L11** (high-dose group) | | |
| --- | --- | --- | --- | --- | --- | --- | --- | --- | --- | --- |
| ♀ n= | | 5 | | | 5 | | | 5 | | |
| ALB | (g/L) | 34.0 | ± | 0.7 | 33.8 | ± | 1.9 | 34.1 | ± | 0.7 |
| TP | (g/L) | 59.5 | ± | 1.5 | 58.4 | ± | 3.1 | 59.2 | ± | 2.1 |
| AST | (u/L) | 199 | ± | 22 | 162 | ± | 38 | 142 | ± | 40* |
| TCHO | (mmol/L) | 1.69 | ± | 0.48 | 1.60 | ± | 0.30 | 1.31 | ± | 0.13 |
| ALP | (u/L) | 152 | ± | 46 | 136 | ± | 28 | 157 | ± | 48 |
| UREA | (mmol/L) | 5.22 | ± | 0.89 | 5.24 | ± | 0.65 | 5.00 | ± | 0.47 |
| TBIL | (umol/L) | 0.53 | ± | 0.18 | 0.68 | ± | 0.20 | 0.87 | ± | 0.50 |
| GLU | (mmol/L) | 5.32 | ± | 0.71 | 4.56 | ± | 0.74 | 6.29 | ± | 0.25* |
| TG | (mmol/L) | 0.34 | ± | 0.21 | 0.30 | ± | 0.17 | 0.22 | ± | 0.06 |
| ALT | (u/L) | 32.8 | ± | 7.6 | 29.2 | ± | 3.6 | 29.1 | ± | 5.5 |
| CREA | (umol/L) | 33.6 | ± | 3.8 | 39.5 | ± | 6.8 | 31.0 | ± | 3.9 |
| ♂ n= | | 5 | | | 5 | | | 5 | | |
| ALB | (g/L) | 27.4 | ± | 14.8 | 26.4 | ± | 14.5 | 33.5 | ± | 0.7 |
| TP | (g/L) | 53.7 | ± | 11.7 | 52.5 | ± | 11.4 | 59.2 | ± | 1.6 |
| AST | (u/L) | 234 | ± | 48 | 185 | ± | 59 | 110 | ± | 9* |
| TCHO | (mmol/L) | 7.10 | ± | 12.13 | 6.90 | ± | 12.52 | 1.56 | ± | 0.26 |
| ALP | (u/L) | 237 | ± | 106 | 224 | ± | 110 | 233 | ± | 22 |
| UREA | (mmol/L) | 5.78 | ± | 0.89 | 5.10 | ± | 1.26 | 4.96 | ± | 0.57 |
| TBIL | (umol/L) | 8.17 | ± | 17.07 | 8.64 | ± | 18.65 | 0.54 | ± | 0.24 |
| GLU | (mmol/L) | 3.81 | ± | 1.89 | 4.07 | ± | 2.30 | 6.37 | ± | 0.79* |
| TG | (mmol/L) | 0.75 | ± | 0.82 | 0.69 | ± | 0.74 | 0.64 | ± | 0.18 |
| ALT | (u/L) | 80.4 | ± | 95.7 | 57.4 | ± | 45.6 | 36.1 | ± | 4.0 |
| CREA | (umol/L) | 29.7 | ± | 16.5 | 26.1 | ± | 12.3 | 27.8 | ± | 1.3 |

*p<0.05, **p<0.01, compared to the control group.

**Supplementary Table 13.** Effects on serum test indicators in rats after 2 weeks administered of **L11** and 2 weeks of recovery

| Items | | Control | | | **L11**(low-dose group) | | | **L11** (high-dose group) | | |
| --- | --- | --- | --- | --- | --- | --- | --- | --- | --- | --- |
| ♀ n= | | 5 | | | 5 | | | 5 | | |
| ALB | (g/L) | 34.5 | ± | 3.0 | 33.2 | ± | 1.5 | 36.4 | ± | 2.0 |
| TP | (g/L) | 61.6 | ± | 6.5 | 57.4 | ± | 3.8 | 64.4 | ± | 3.0 |
| AST | (u/L) | 173 | ± | 50 | 163 | ± | 24 | 160 | ± | 19 |
| TCHO | (mmol/L) | 1.85 | ± | 0.48 | 1.35 | ± | 0.48 | 1.72 | ± | 0.26 |
| ALP | (u/L) | 146 | ± | 51 | 124 | ± | 23 | 119 | ± | 13 |
| UREA | (mmol/L) | 6.68 | ± | 0.93 | 6.55 | ± | 1.04 | 6.75 | ± | 0.73 |
| TBIL | (umol/L) | 0.73 | ± | 0.38 | 1.00 | ± | 0.32 | 1.12 | ± | 0.29 |
| GLU | (mmol/L) | 6.95 | ± | 1.92 | 5.57 | ± | 1.02 | 6.05 | ± | 0.31 |
| TG | (mmol/L) | 0.45 | ± | 0.15 | 0.25 | ± | 0.03* | 0.29 | ± | 0.06 |
| ALT | (u/L) | 40.9 | ± | 11.1 | 37.8 | ± | 9.3 | 28.4 | ± | 4.4* |
| CREA | (umol/L) | 40.6 | ± | 5.8 | 40.1 | ± | 4.1 | 39.1 | ± | 4.9 |
| ♂ n= | | 5 | | | 5 | | | 5 | | |
| ALB | (g/L) | 33.0 | ± | 2.8 | 31.6 | ± | 0.3 | 32.9 | ± | 1.5 |
| TP | (g/L) | 59.9 | ± | 6.9 | 56.7 | ± | 1.5 | 58.9 | ± | 2.9 |
| AST | (u/L) | 187 | ± | 53 | 176 | ± | 26 | 160 | ± | 48 |
| TCHO | (mmol/L) | 1.62 | ± | 0.30 | 1.43 | ± | 0.13 | 1.58 | ± | 0.22 |
| ALP | (u/L) | 202 | ± | 98 | 191 | ± | 45 | 172 | ± | 20 |
| UREA | (mmol/L) | 5.82 | ± | 0.65 | 5.02 | ± | 0.29* | 6.31 | ± | 1.04 |
| TBIL | (umol/L) | 0.69 | ± | 0.71 | 0.62 | ± | 0.18 | 0.77 | ± | 0.23 |
| GLU | (mmol/L) | 5.97 | ± | 1.61 | 5.87 | ± | 0.68 | 6.70 | ± | 1.12 |
| TG | (mmol/L) | 0.49 | ± | 0.16 | 0.36 | ± | 0.15 | 0.59 | ± | 0.42 |
| ALT | (u/L) | 40.4 | ± | 12.2 | 34.4 | ± | 4.1 | 35.0 | ± | 5.7 |
| CREA | (umol/L) | 33.0 | ± | 3.2 | 30.4 | ± | 2.6 | 32.8 | ± | 2.3 |

*p<0.05, **p<0.01, compared to the control group.

**Supplementary Data 1. ^1^HNMR and ^13^CNMR spectrums of the targeted compounds.**

**
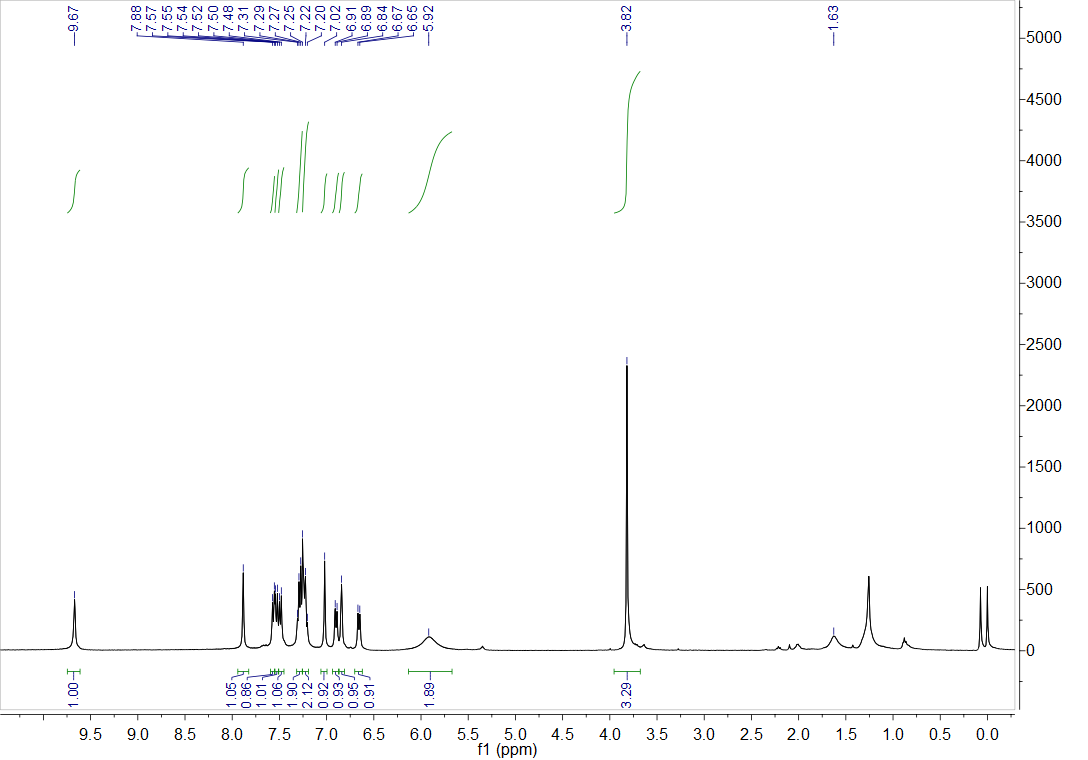
**

**
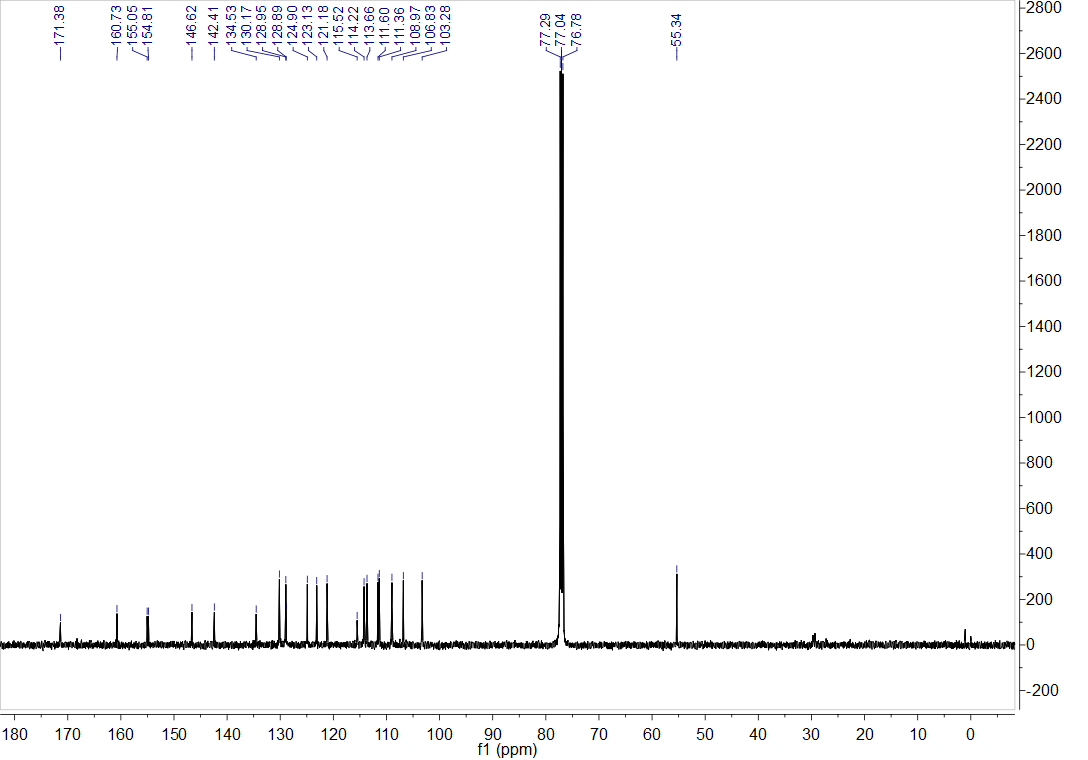
**


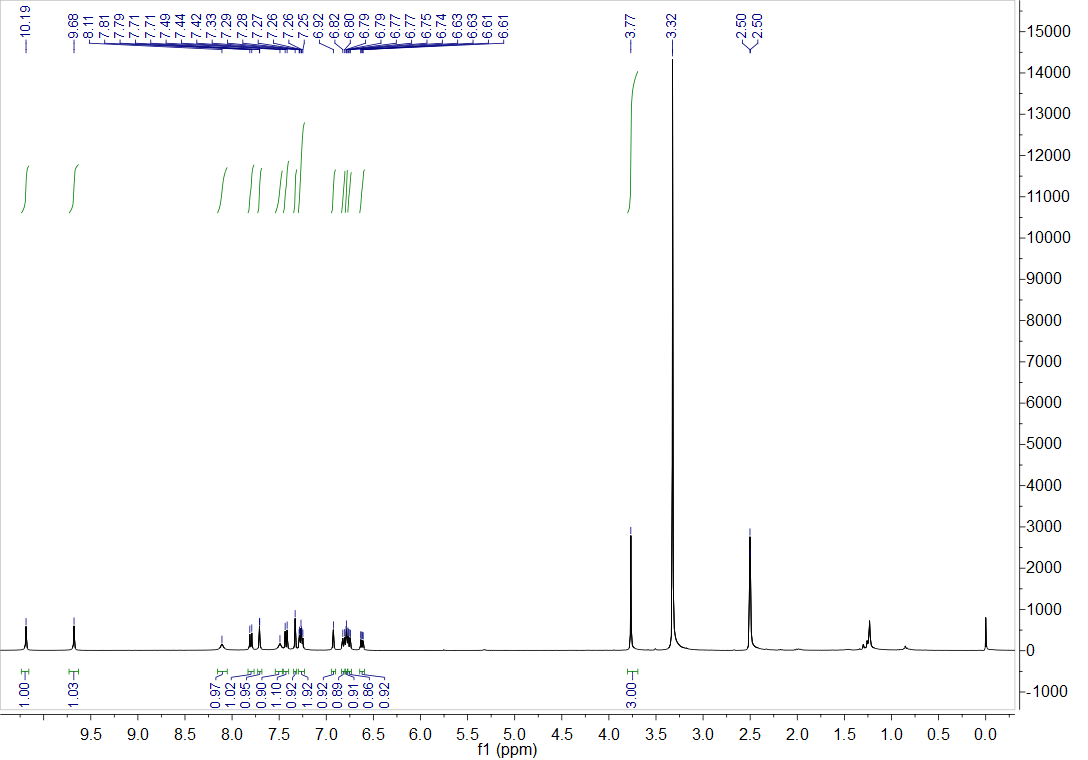


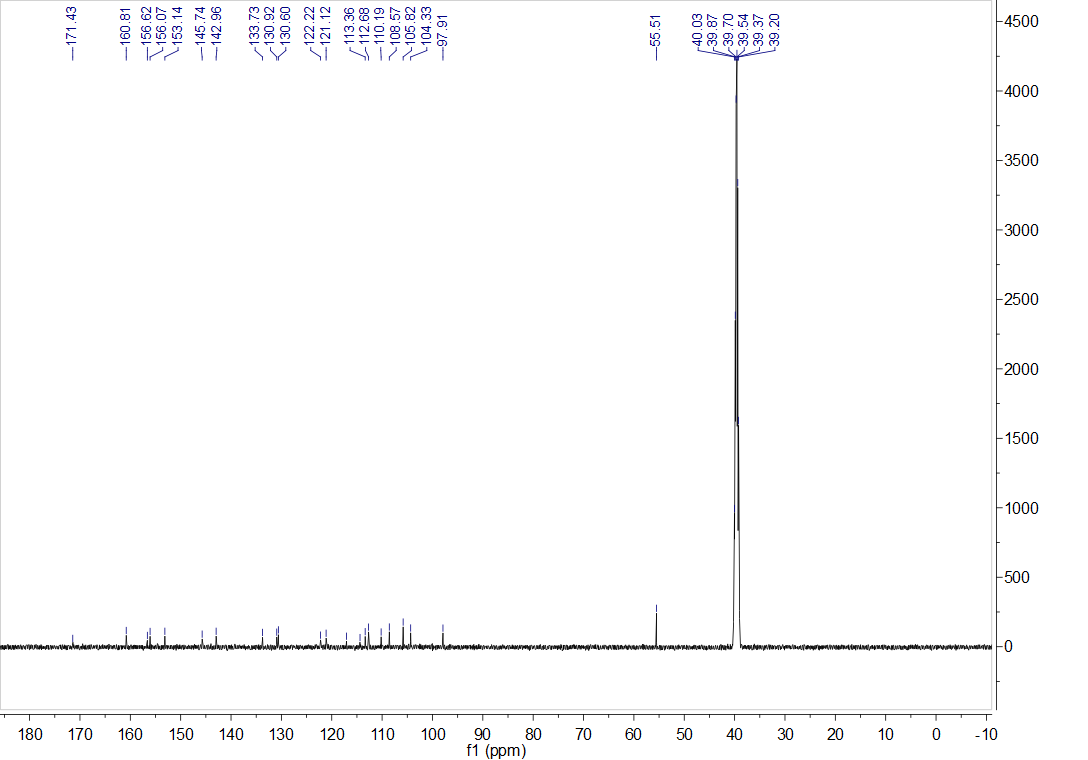


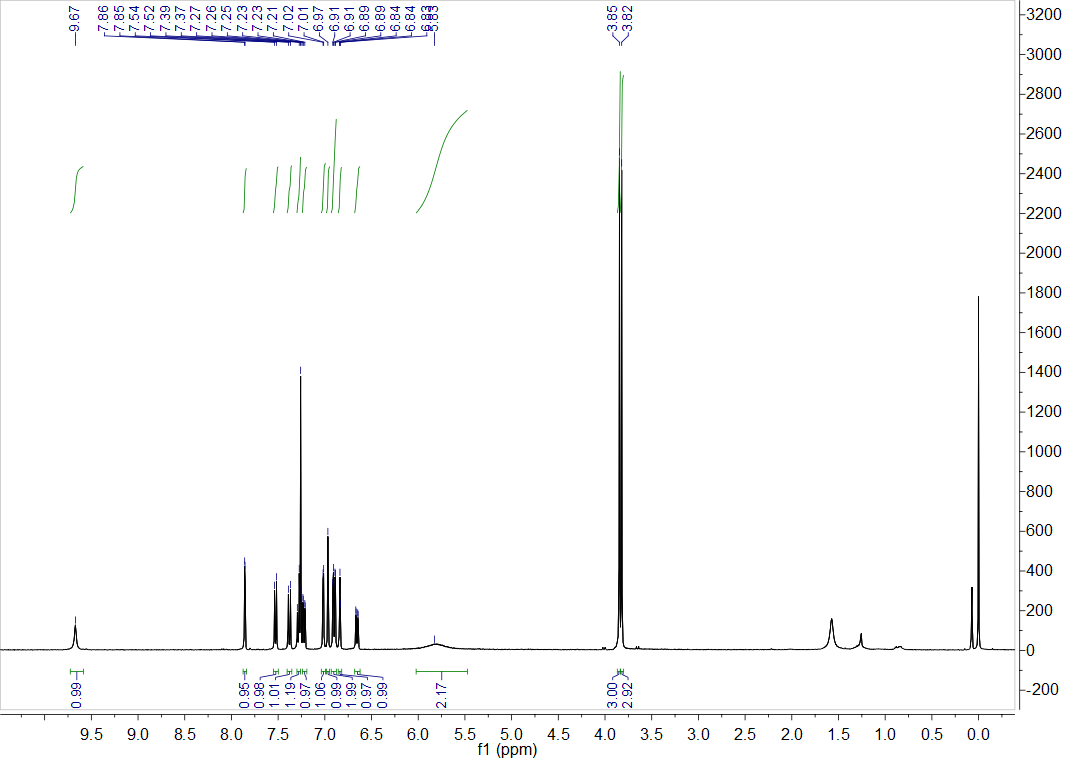


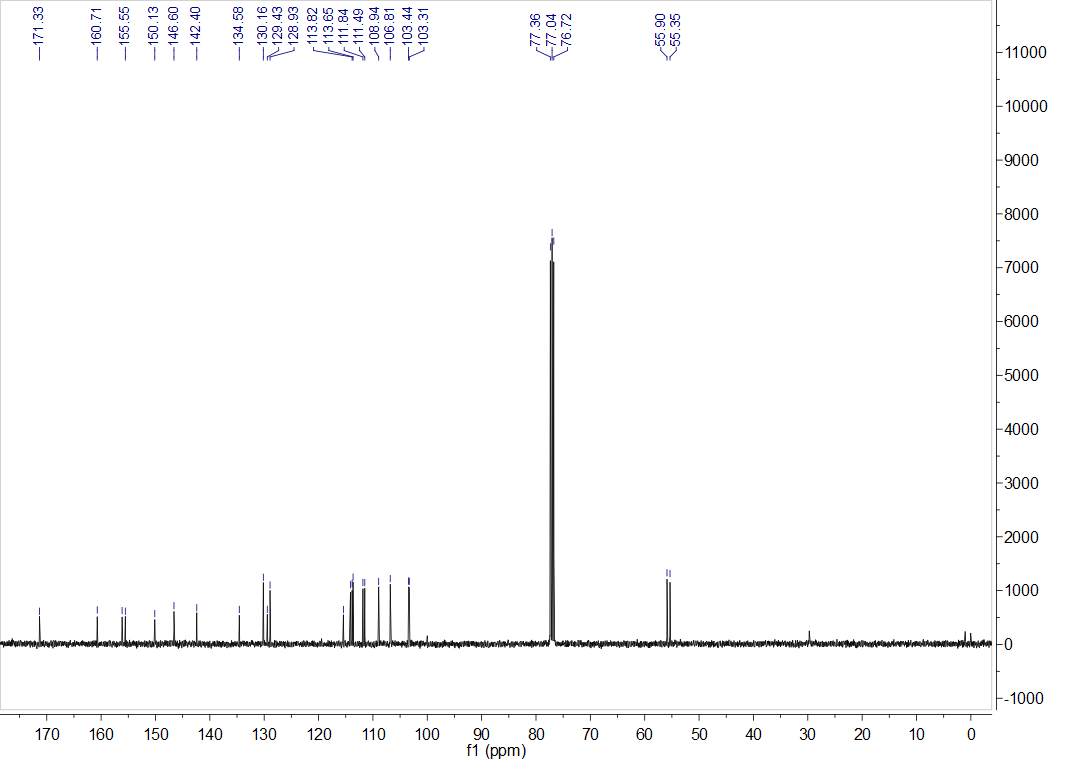


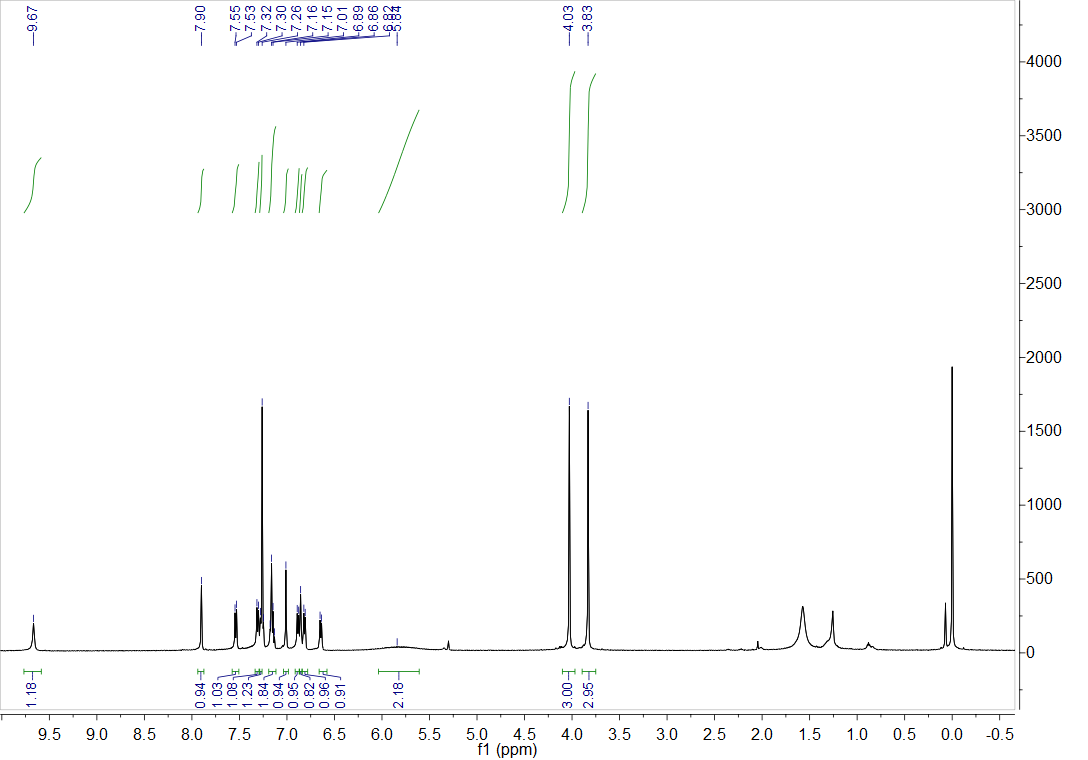


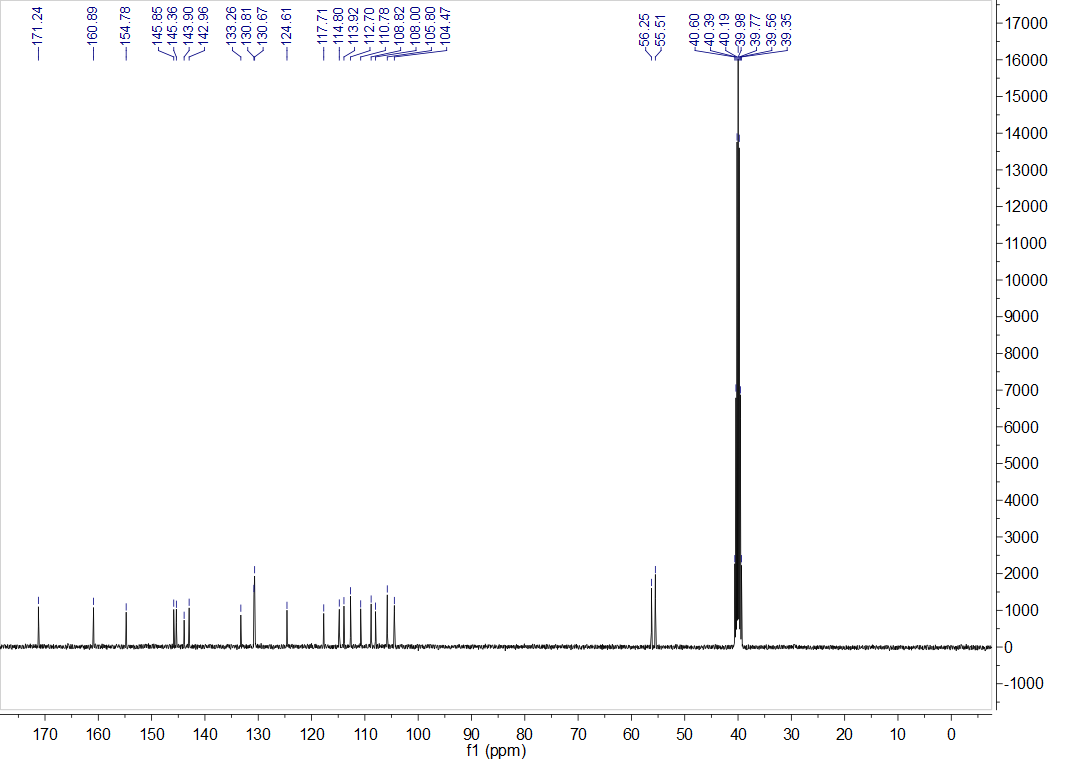


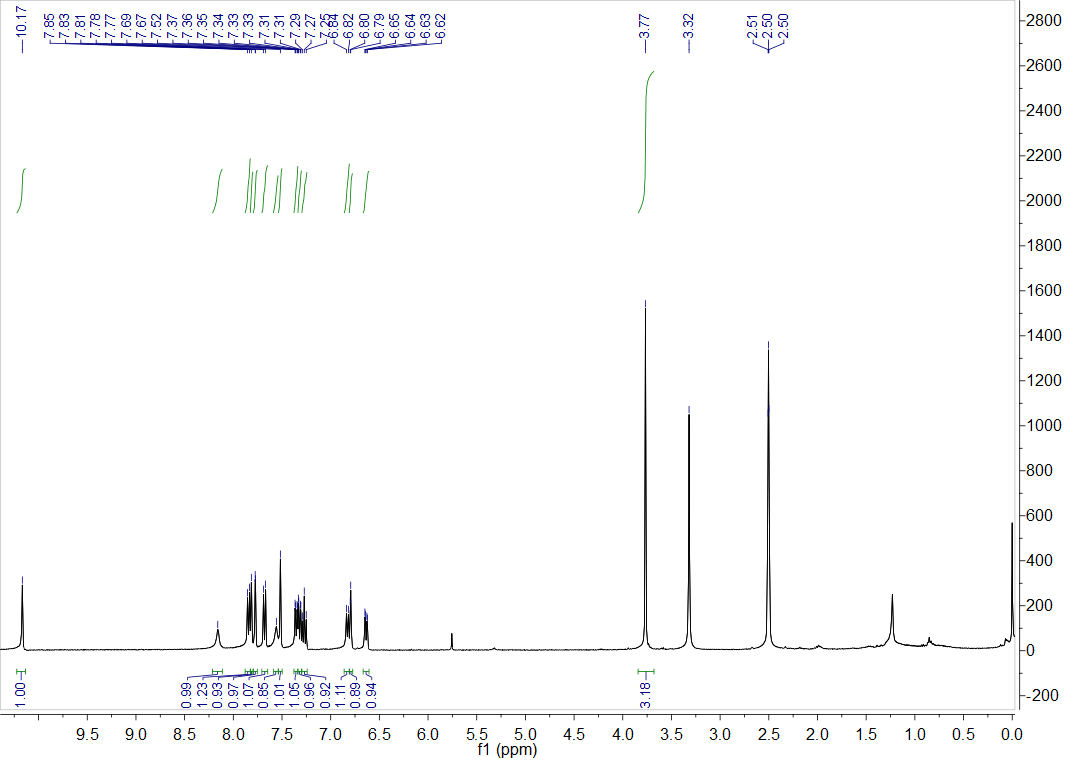


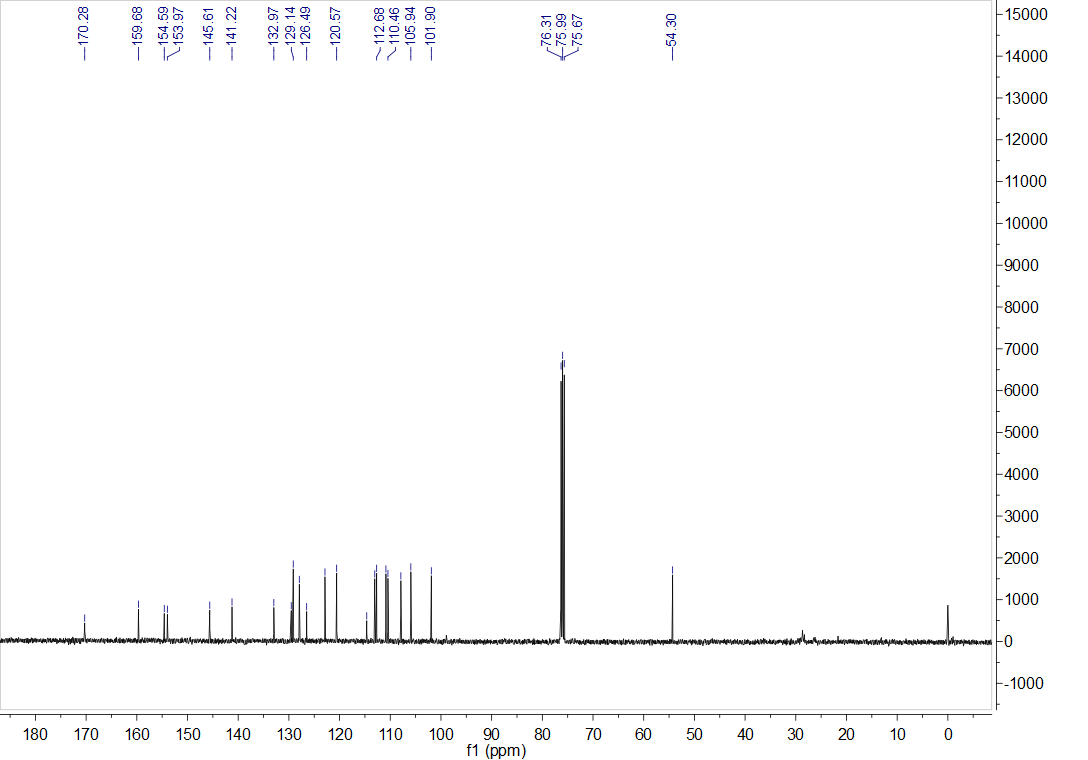


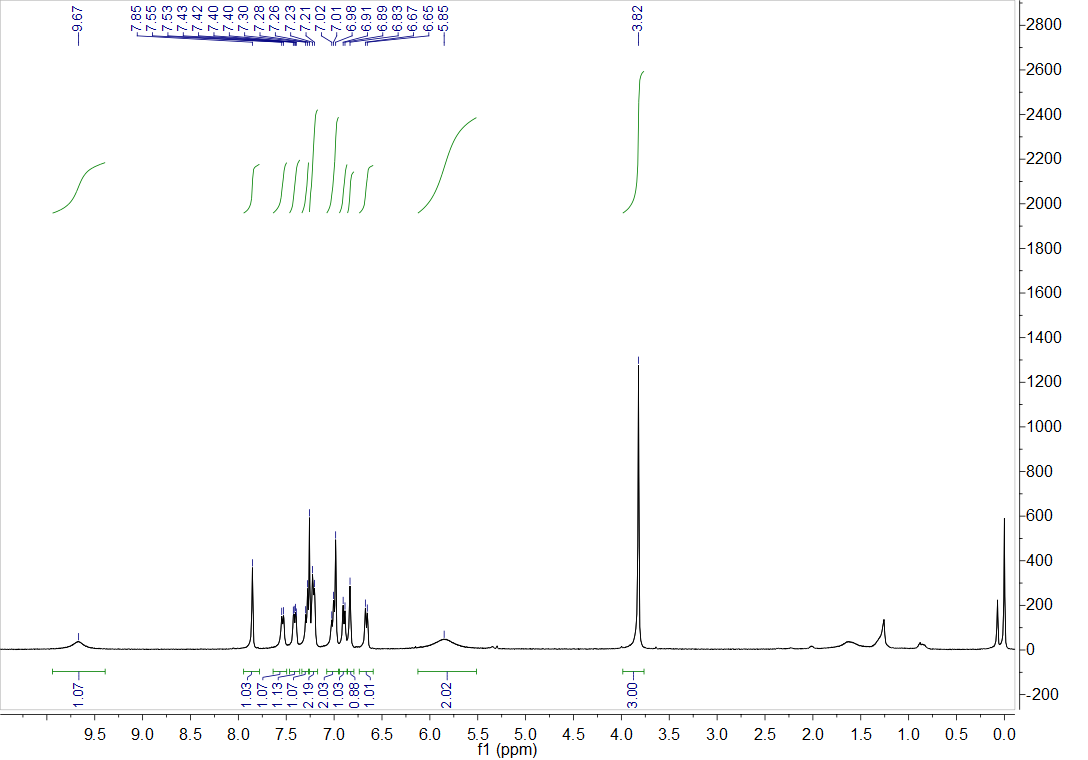


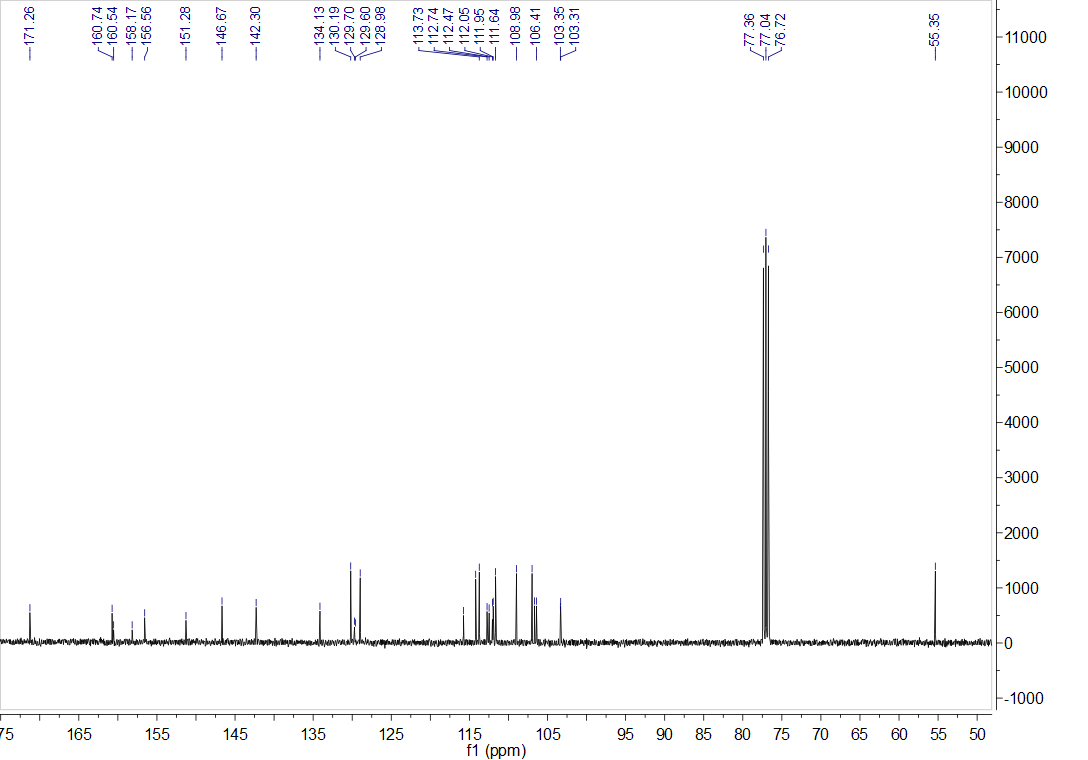


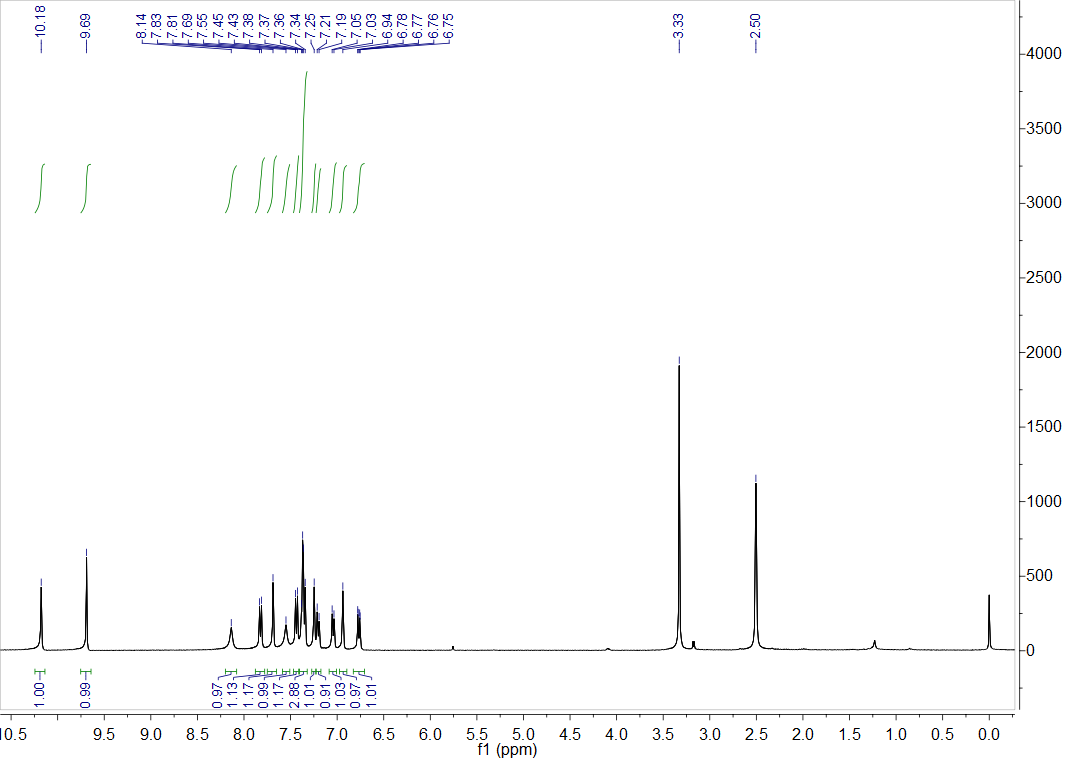


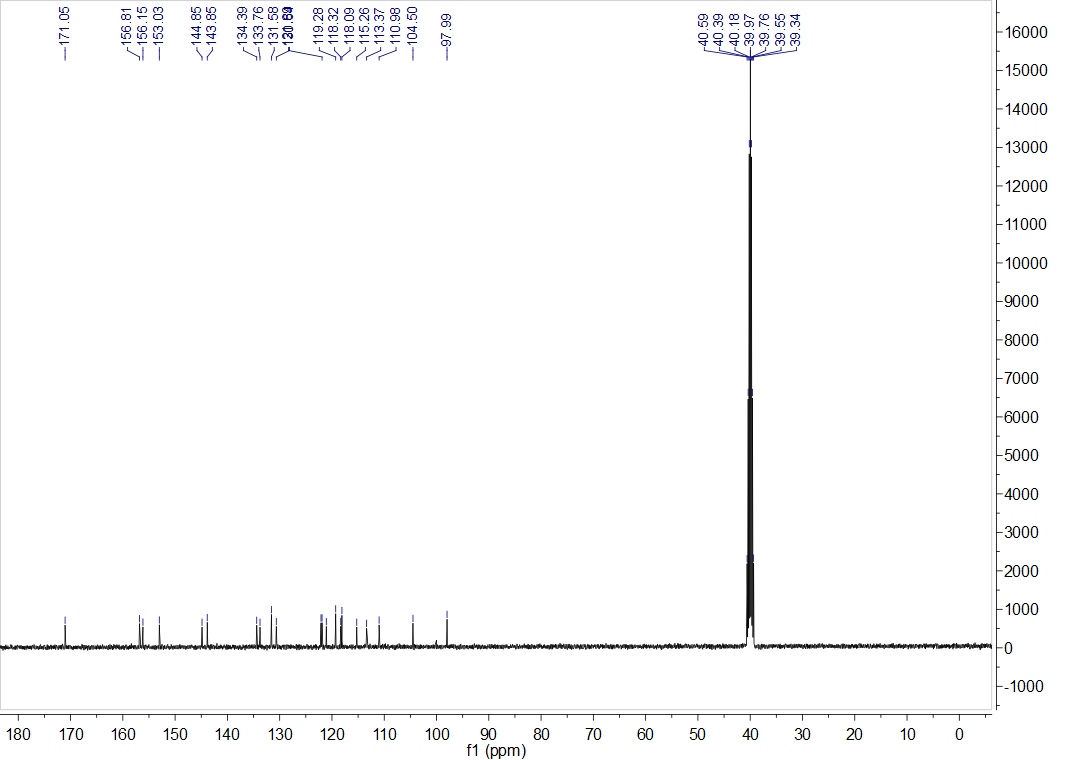


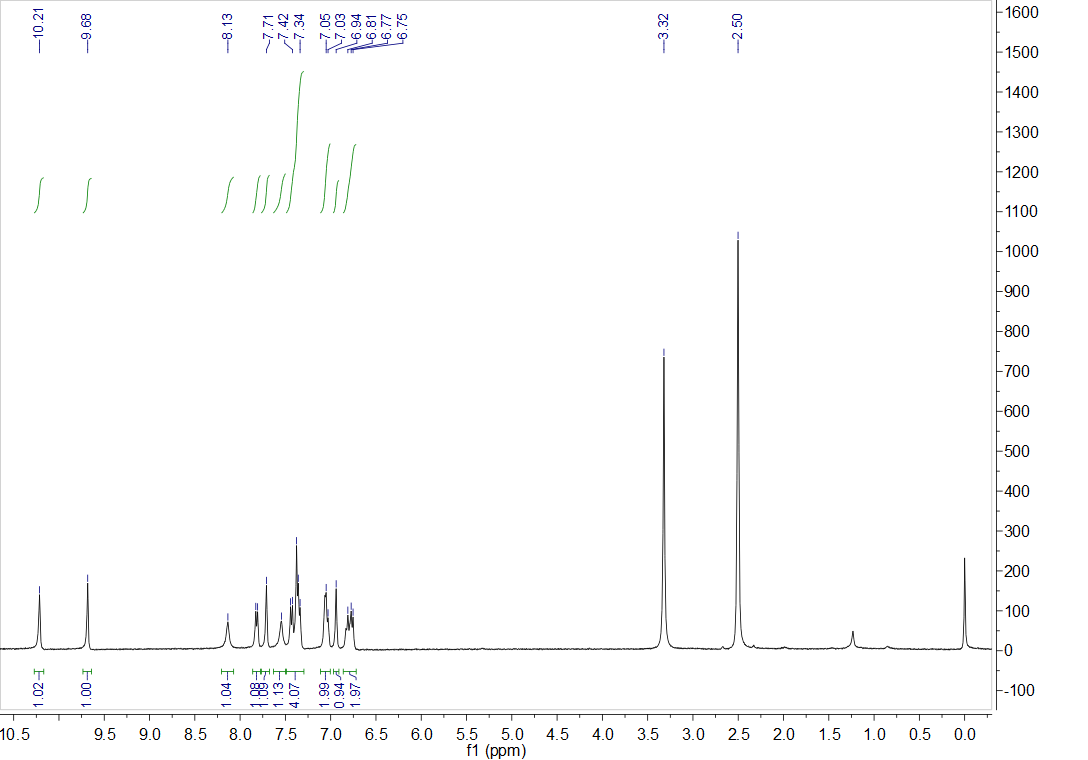


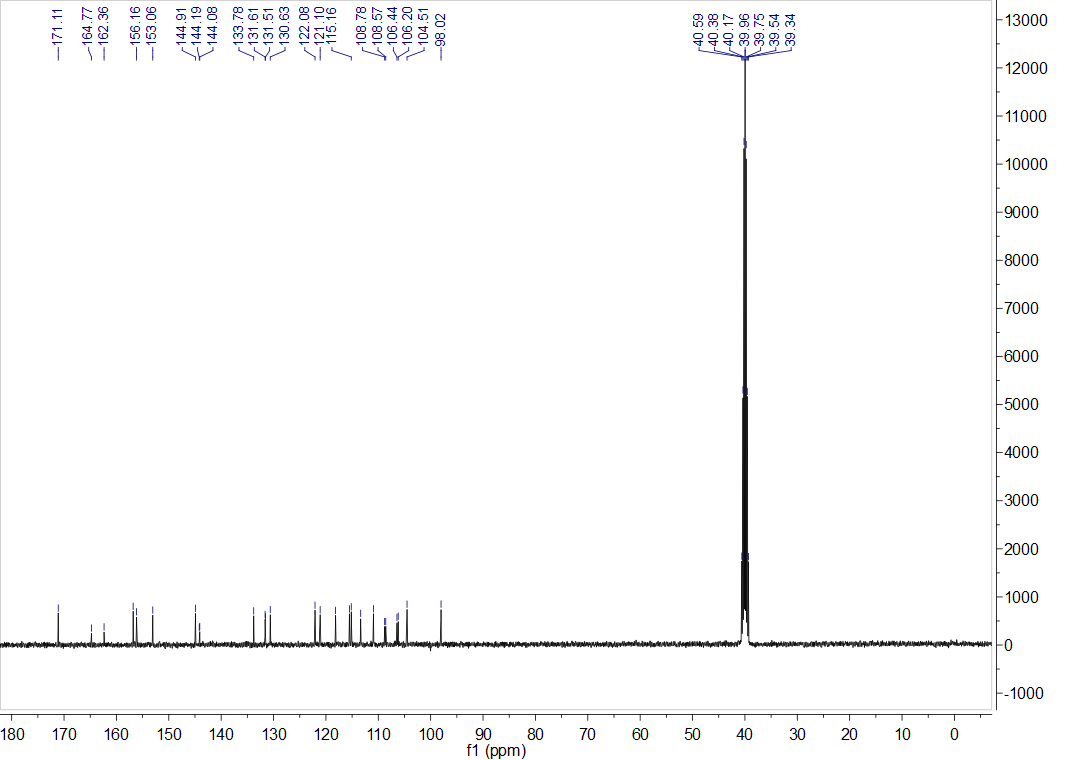


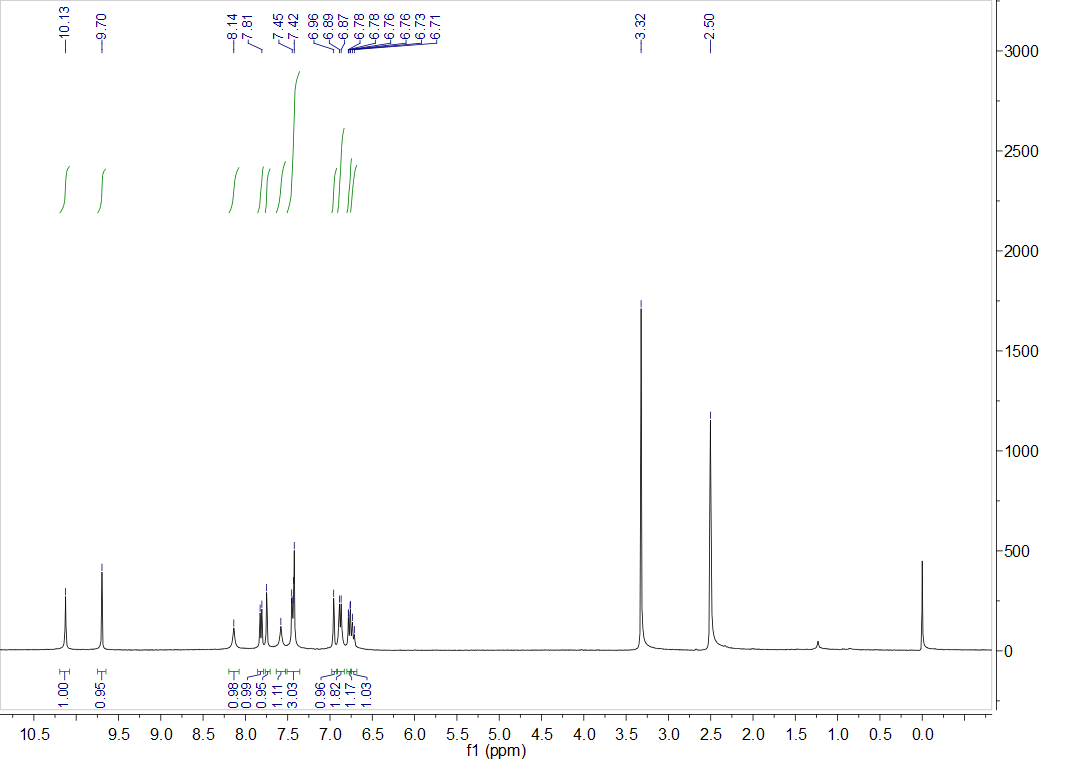


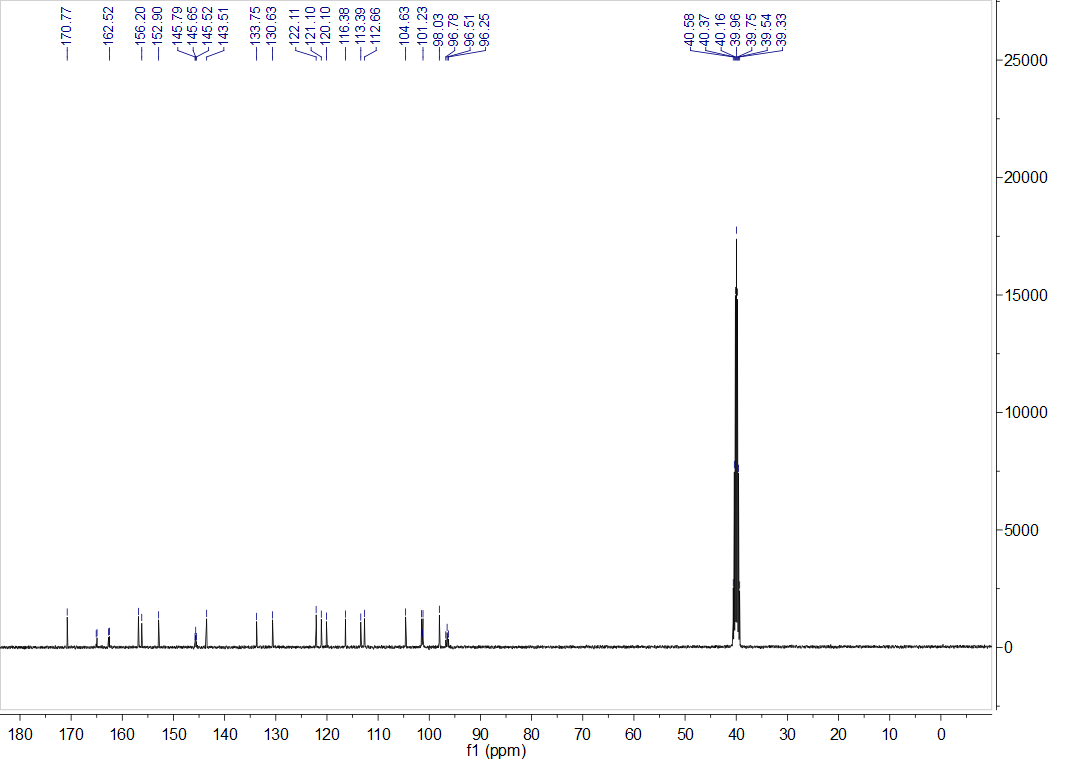


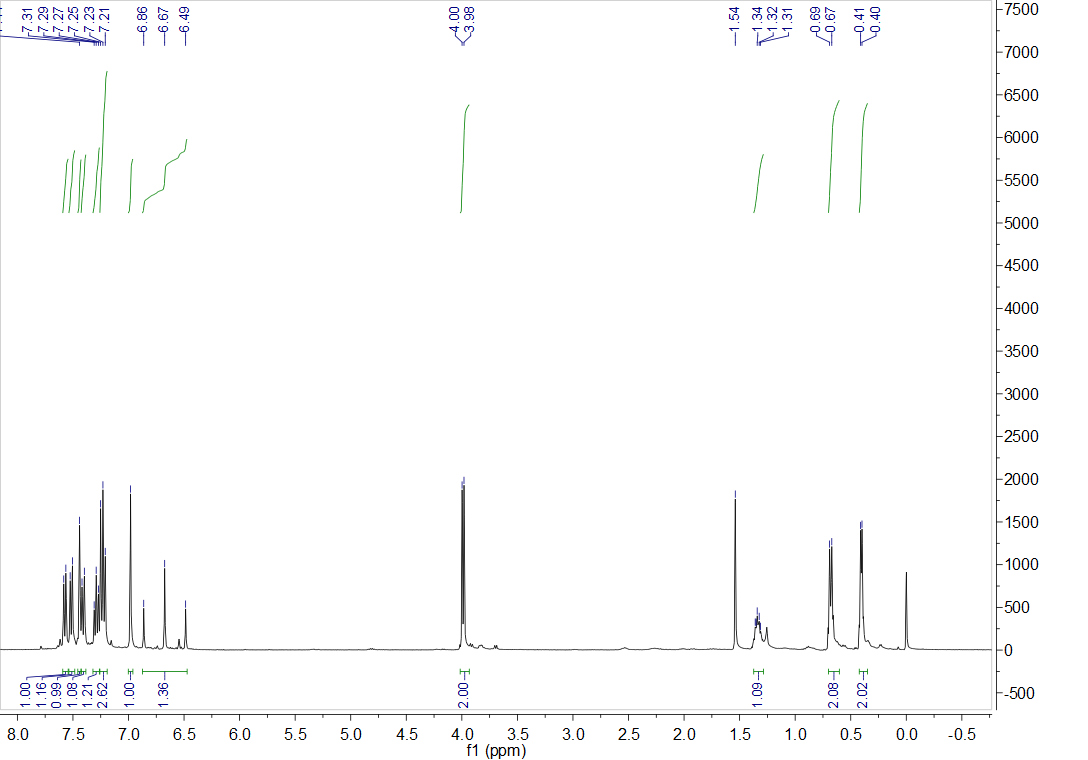


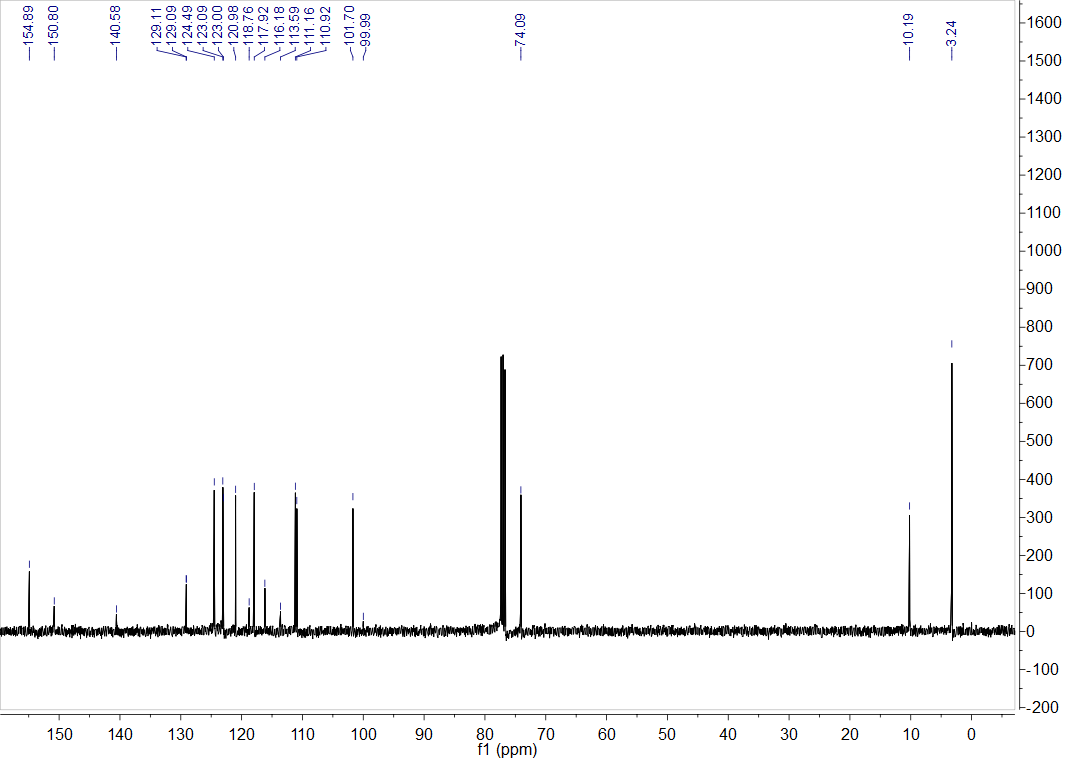


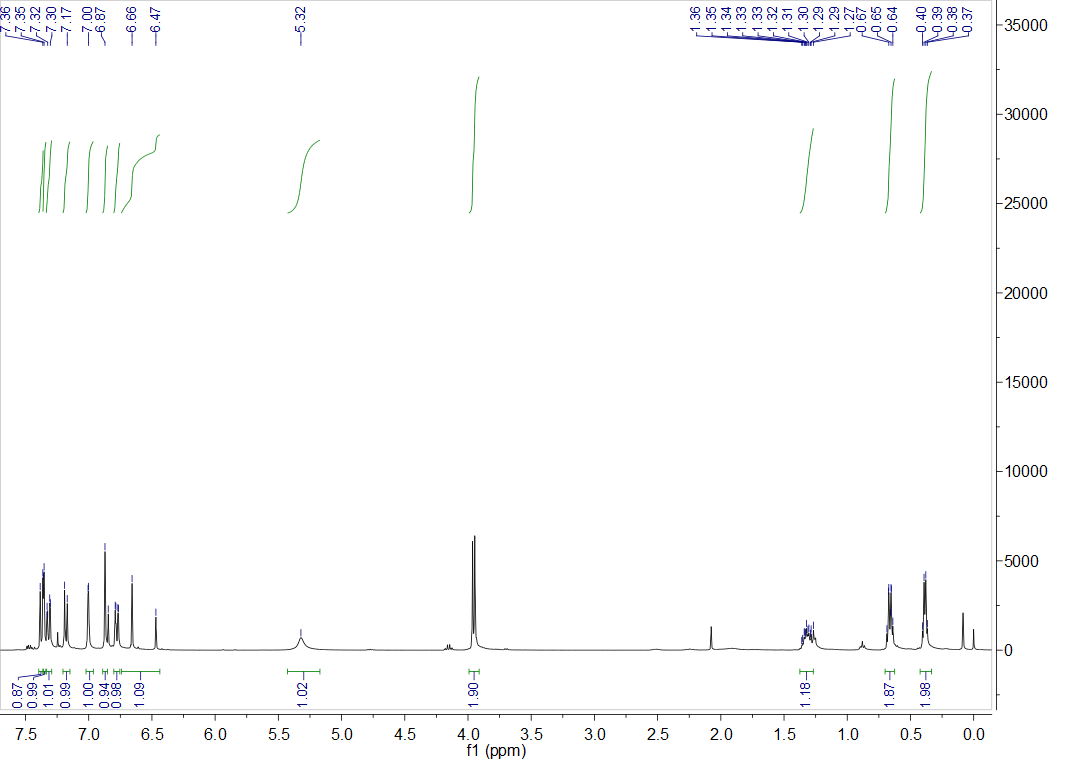


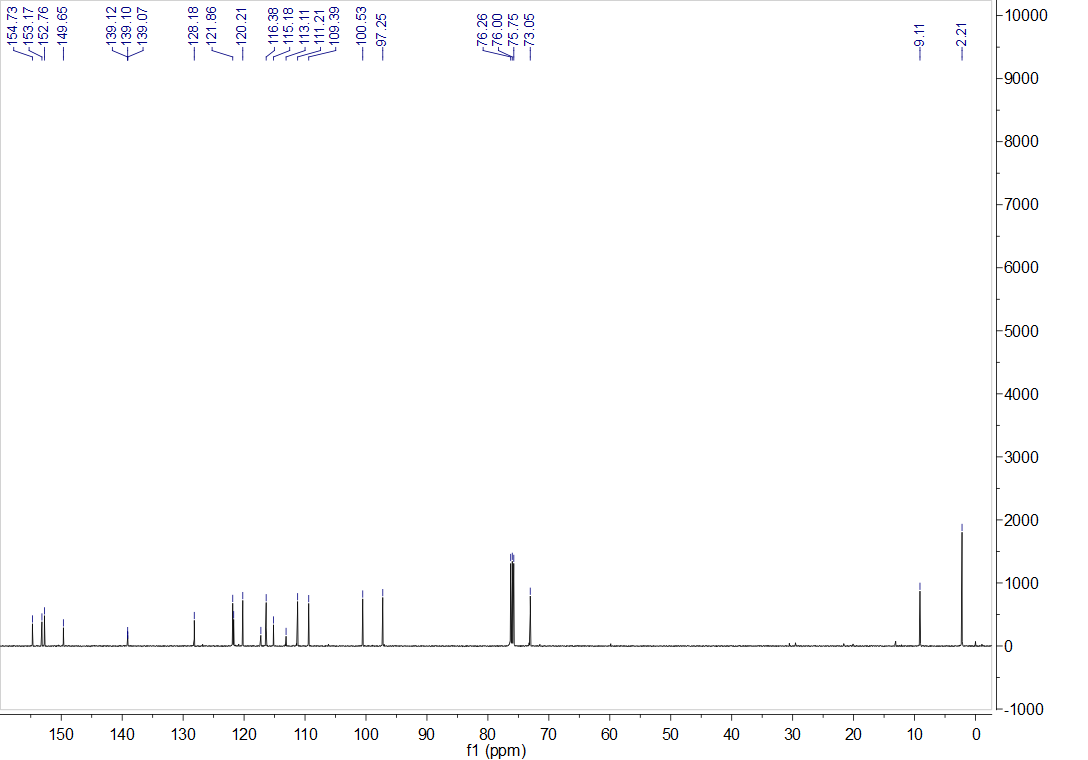


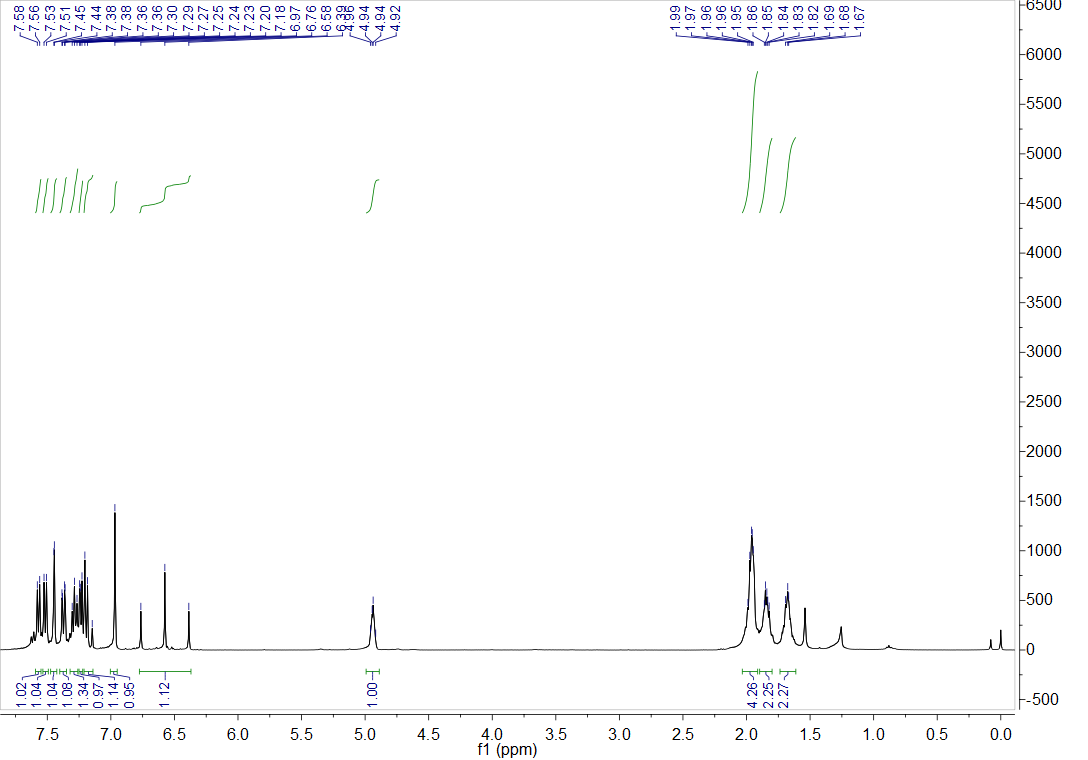


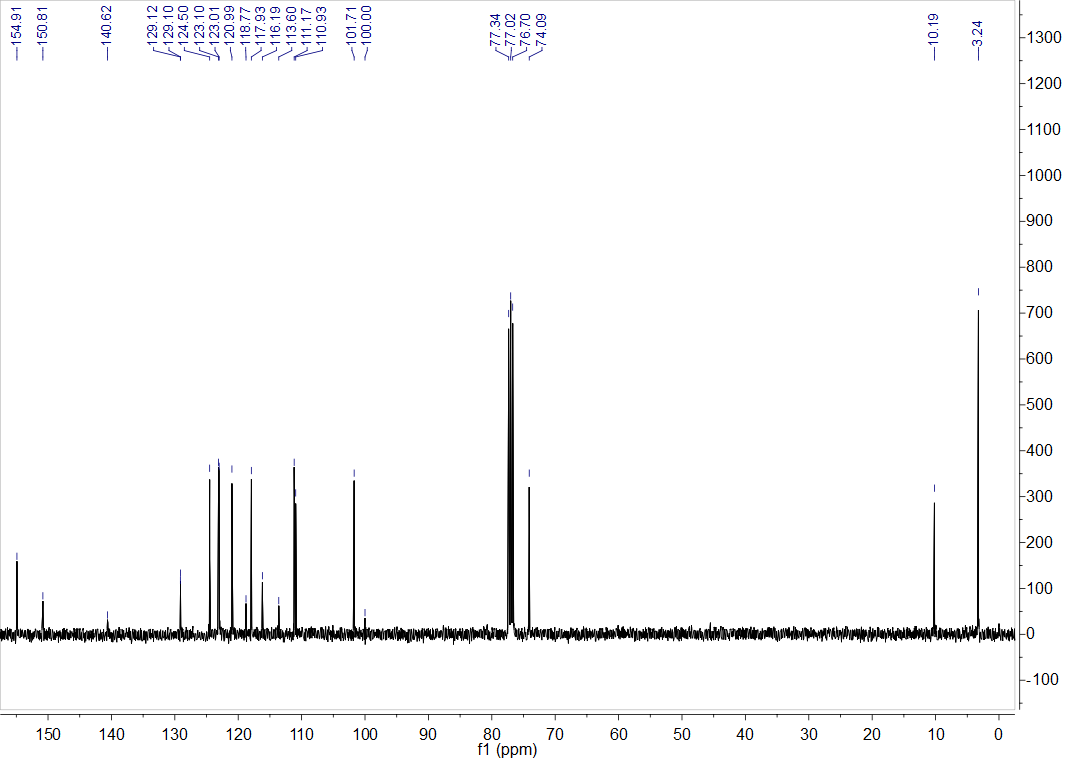


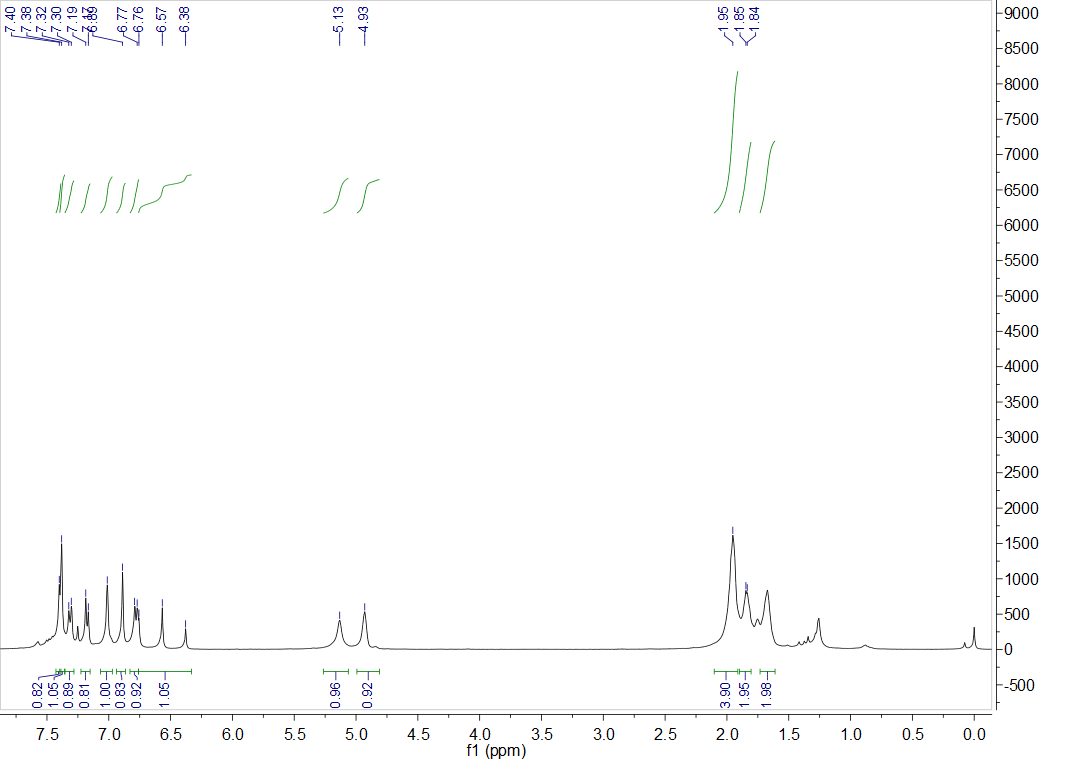


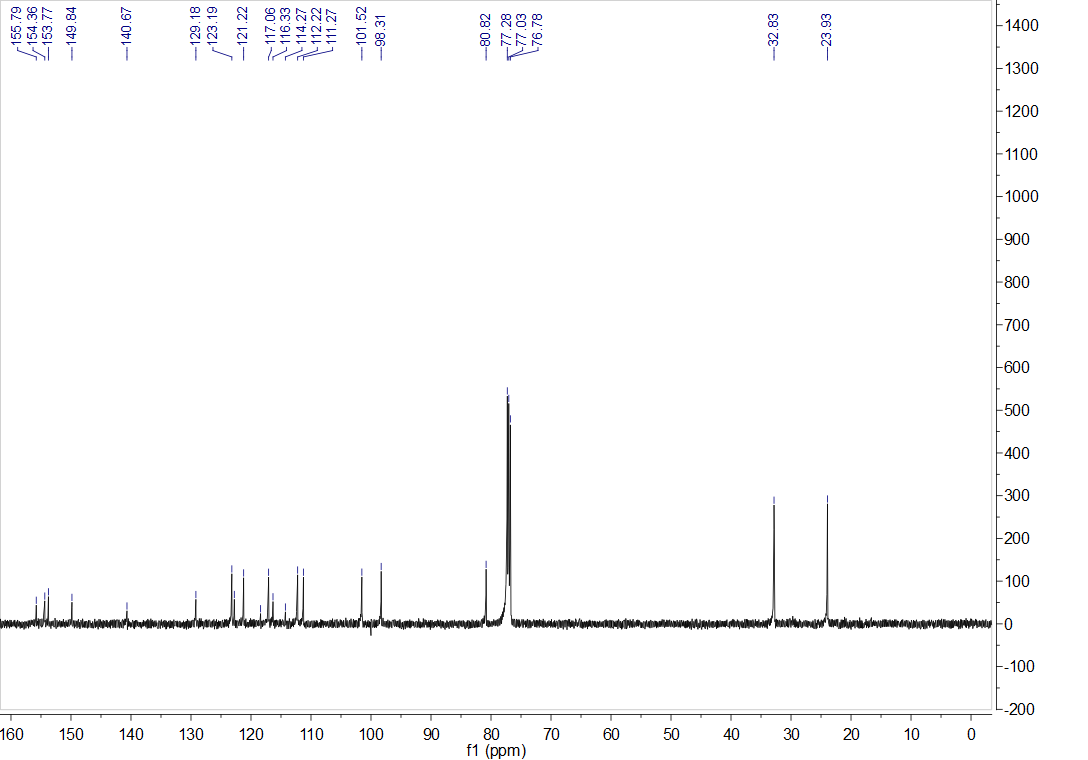


**Supplementary Data 2. The HRMS spectrums of the target compounds.**


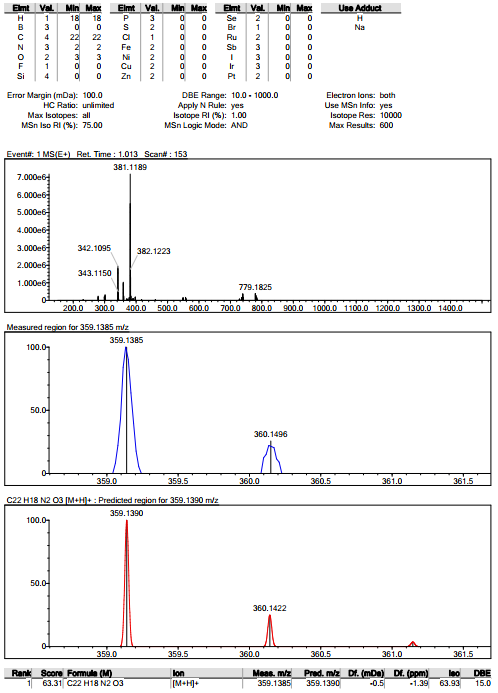


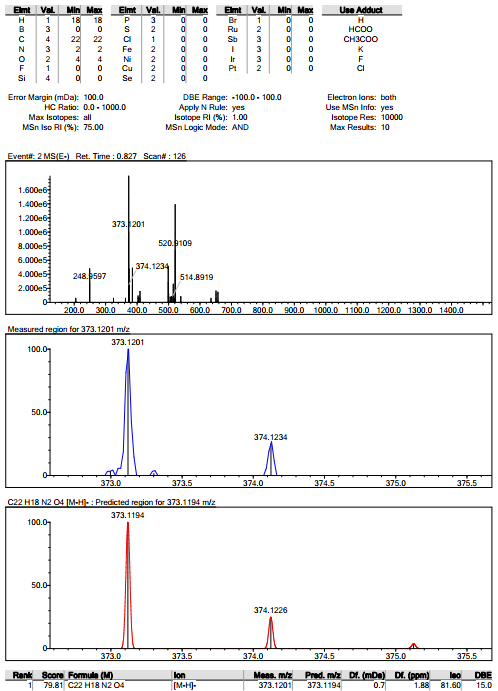


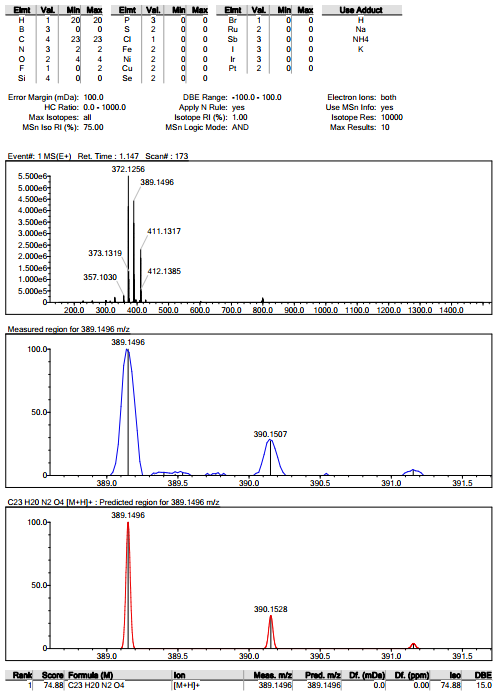

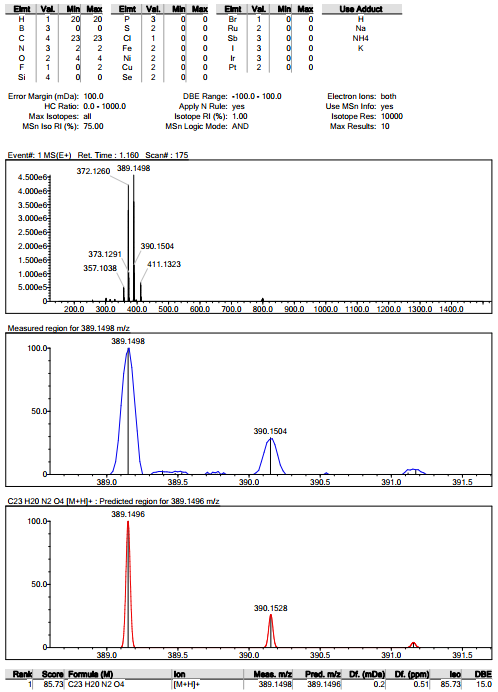


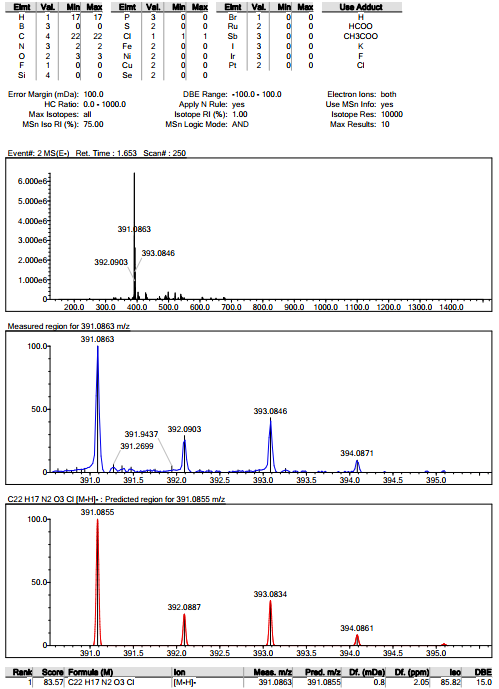


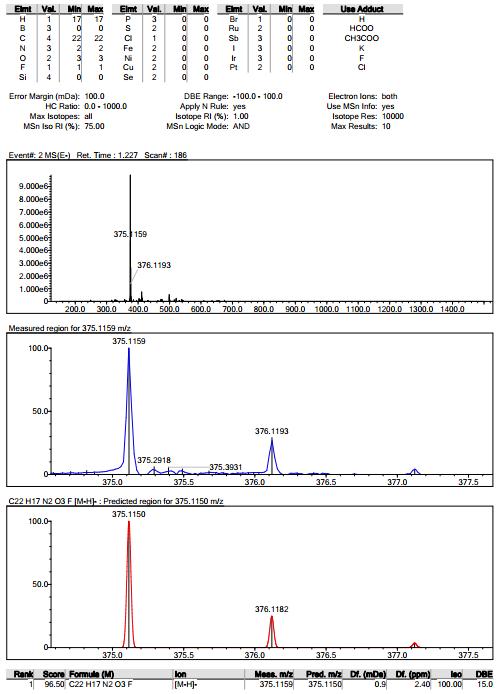


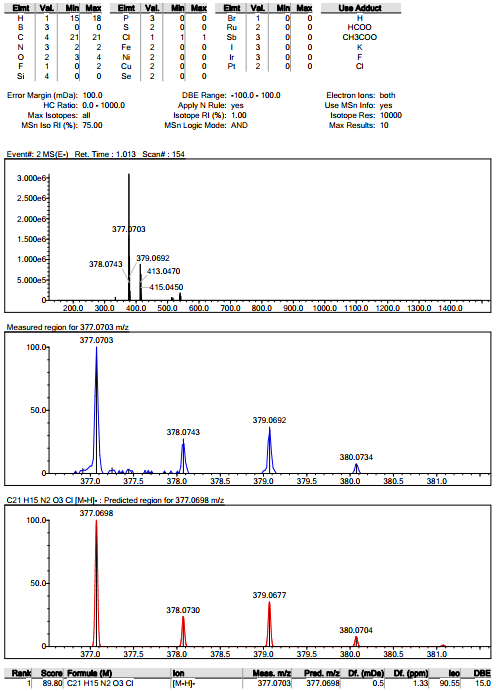


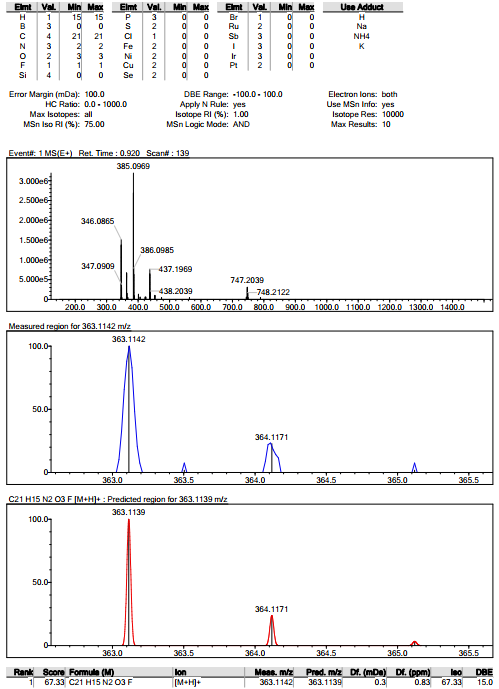


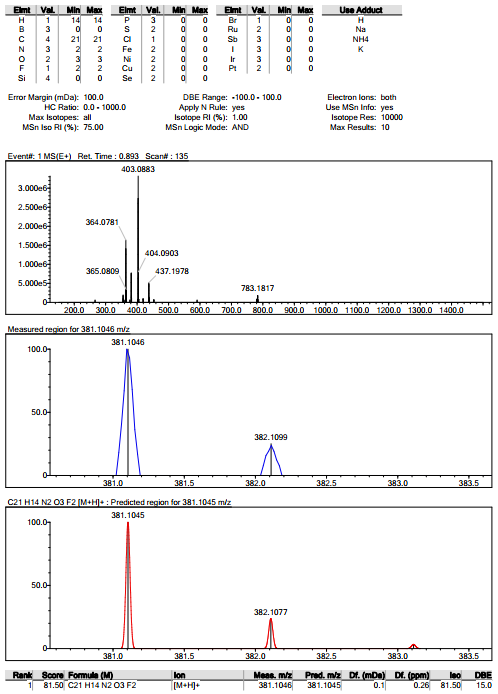


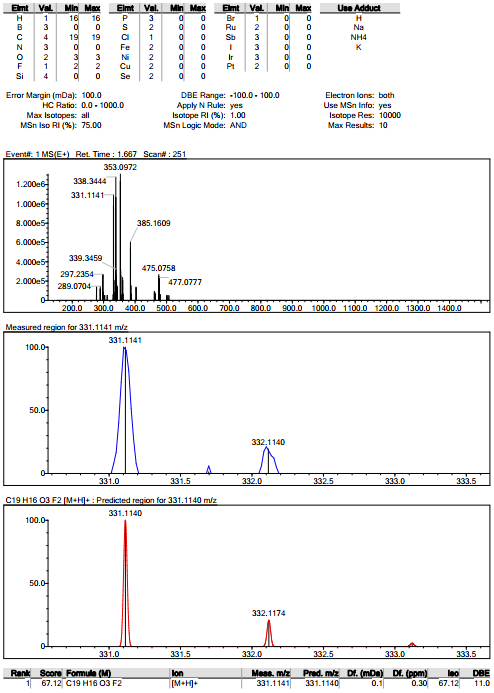


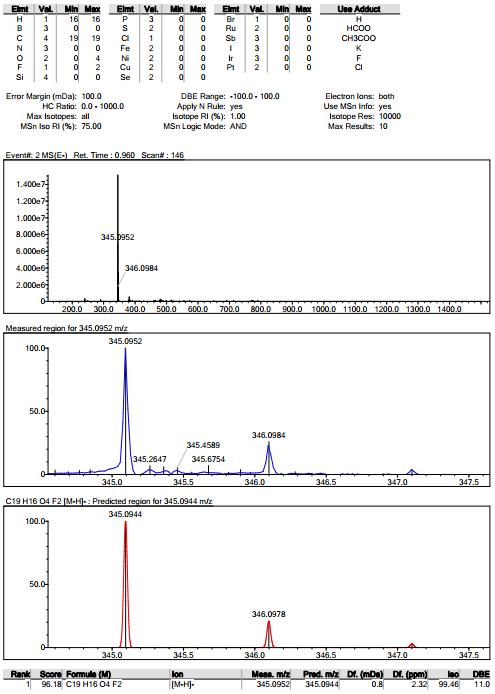


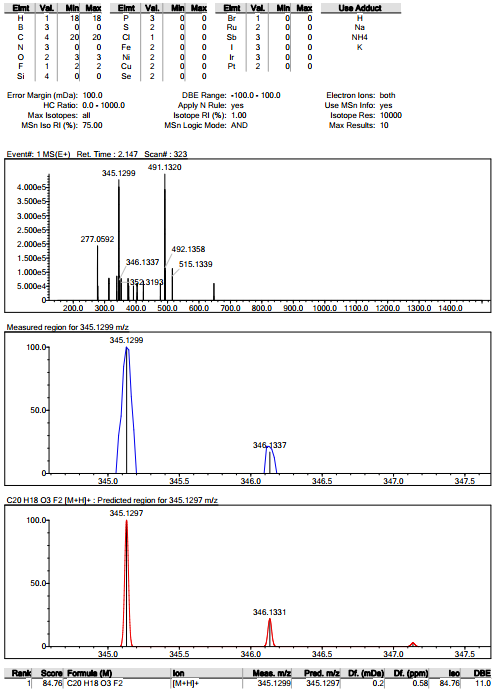


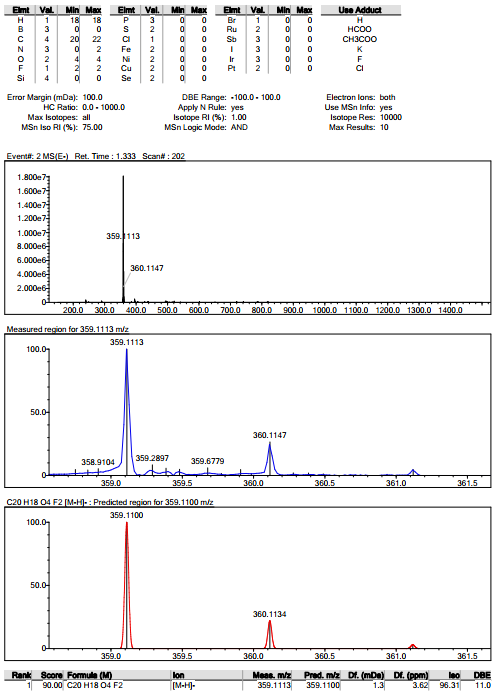


**Supplementary Data 2. General procedure for synthesis of** **chemicals**

The general synthetic procedures for intermediates 3a-3f have been reported in our previous study (*106*) and the products were used directly in the next step without further purification.

**General procedure for synthesis of intermediates 6a-6d.**

To a mixture of 4 (0.5 mmol), 5 (0.75 mmol) and xantphos (0.05 mmol) in toluene (4.0 mL) was added Pd(OAc)_2_ (0.025 mmol) and Cs_2_CO_3_(0.75 mmol). The reaction was stirred at 115 ℃ for 24 h under Ar atmosphere (28324649). After cooling to room temperature, the mixture was extracted with portions of ethyl acetate. The combined organic extracts were dried over anhydrous sodium sulfate, concentrated and purified by silica gel column chromatography to get the compounds 6a-6d, respectively.

4-Bromo-2-((3-methoxyphenyl)amino)benzonitrile (**6a**). Yield 51.8%. ^1^H NMR (400 MHz, CDCl_3_) *δ* 7.36 – 7.32 (m, 2H), 7.30 (d, *J* = 8.1 Hz, 1H), 6.96 (dd, *J* = 8.3, 1.5 Hz, 1H), 6.79 (d, *J* = 7.9 Hz, 1H), 6.77 – 6.71 (m, 2H), 6.33 (s, 1H), 3.83 (s, 3H).

4-Bromo-2-((3-chlorophenyl)amino)benzonitrile (**6b**). Yield 77.1%. ^1^H NMR (400 MHz, CDCl_3_) *δ* 7.37 (d, *J* = 8.3 Hz, 1H), 7.34 (d, *J* = 8.1 Hz, 1H), 7.31 (s, 1H), 7.20 (t, *J* = 2.0 Hz, 1H), 7.17 (ddd, *J* = 8.0, 1.7, 0.8 Hz, 1H), 7.09 (dd, *J* = 8.1, 1.4 Hz, 1H), 7.03 (dd, *J* = 8.3, 1.7 Hz, 1H), 6.33 (s, 1H).

4-Bromo-2-((3-fluorophenyl)amino)benzonitrile (**6c**). Yield 95%. ^1^H NMR (400 MHz, CDCl_3_) *δ* 7.39 – 7.32 (m, 3H), 7.03 (dd, *J* = 8.4, 1.6 Hz, 1H), 6.97 (dd, *J* = 8.0, 1.7 Hz, 1H), 6.95 – 6.90 (m, 1H), 6.87 (dd, *J* = 8.3, 2.3 Hz, 1H), 6.36 (s, 1H).

4-Bromo-2-((3,5-difluorophenyl)amino)benzonitrile (**6d**). Yield 80.3%. ^1^H NMR (400 MHz, CDCl_3_) *δ* 7.44 (d, *J* = 1.6 Hz, 1H), 7.40 (d, *J* = 8.3 Hz, 1H), 7.11 (dd, *J* = 8.3, 1.7 Hz, 1H), 6.70 (dt, *J* = 7.0, 3.5 Hz, 2H), 6.60 (tt, *J* = 8.9, 2.2 Hz, 1H), 6.36 (s, 1H).

**General procedure for synthesis of compounds L1-L9.**

To a mixture of 3 (1.2 mmol), 6 (1.0 mmol) in deionized water (0.5 mL) and dimethylacetamide (3.0 mL) was added tricyclohexyl phosphine (0.1 mmol), potassium carbonate (1.5 mmol) followed by palladium diacetate (0.05 mmol). The resulting suspension was heated to 80 ℃ under Ar atomosphere and stirred for 2 h when the reaction was monitored complete by TLC. After cooling to room temperature, the mixture was diluted with water and acidified by the addition of 2N aqueous HCl. Then the aqueous solution was extracted with ethyl acetate and the organic layers were dried over anhydrous sodium sulfate, concentrated and purified by silica gel column chromatography to get the target compounds 7a-7i, respectively.1 Then to a solution of compound 7a-7i (0.3 mmol) in water (5 mL) and t-BuOH (8 mL) was added KOH (0.45 mmol). The reaction mixture was stirred at 60 oC for 4 h. After monitored complete by TLC, it was diluted with water and extracted with portions of ethyl acetate, washed with brine, dried over anhydrous sodium sulfate, and concentrated to give a crude, which was purified by column chromatography to afford the product, respectively.

4-(Benzofuran-2-yl)-2-((3-methoxyphenyl)amino)benzamide (**L1**). Yield 86.5%. ^1^H NMR (400 MHz, CDCl_3_) *δ* 9.67 (s, 1H), 7.88 (s, 1H), 7.56 (d, *J* = 7.7 Hz, 1H), 7.53 (d, *J* = 8.4 Hz, 1H), 7.49 (d, *J* = 8.1 Hz, 1H), 7.32 – 7.26 (m, 2H), 7.25 – 7.19 (m, 2H), 7.02 (s, 1H), 6.90 (d, *J* = 7.8 Hz, 1H), 6.84 (s, 1H), 6.66 (d, *J* = 8.0 Hz, 1H), 5.92 (s, 2H), 3.82 (s, 3H). ^13^C NMR (126 MHz, CDCl_3_) *δ* 171.38, 160.73, 155.06, 154.81, 146.62, 142.41, 134.53, 130.17, 128.95, 128.89, 124.90, 123.13, 121.18, 115.52, 114.22, 113.66, 111.60, 111.36, 108.97, 106.83, 103.28, 55.34. HRMS (ESI-TOF) m/z: [M+H]^+^ calcd for C_22_H_18_N_2_O_3_, 359.1390; found, 359.385.

4-(6-Hydroxybenzofuran-2-yl)-2-((3-methoxyphenyl)amino)benzamide (**L2**). Yield 78.6%. ^1^H NMR (400 MHz, DMSO-*d*_6_) *δ* 10.19 (s, 1H), 9.68 (s, 1H), 8.11 (s, 1H), 7.80 (d, *J* = 8.3 Hz, 1H), 7.71 (d, *J* = 1.2 Hz, 1H), 7.49 (s, 1H), 7.43 (d, *J* = 8.4 Hz, 1H), 7.33 (s, 1H), 7.30 – 7.23 (m, 2H), 6.92 (s, 1H), 6.81 (d, *J* = 7.9 Hz, 1H), 6.79 (d, *J* = 1.9 Hz, 1H), 6.76 (dd, *J* = 8.4, 2.0 Hz, 1H), 6.62 (dd, *J* = 8.2, 2.0 Hz, 1H), 3.77 (s, 3H). ^13^C NMR (126 MHz, DMSO-*d*_6_) *δ* 171.43, 160.81, 156.62, 156.07, 153.14, 145.74, 142.96, 133.73, 130.92, 130.60, 122.22, 121.12, 117.07, 114.39, 113.36, 112.68, 110.19, 108.57, 105.82, 104.33, 97.91, 55.51. HRMS (ESI-TOF) m/z: [M-H]^-^ calcd for C_22_H_18_N_2_O_4_, 373.1194; found, 373.1201.

4-(5-Methoxybenzofuran-2-yl)-2-((3-methoxyphenyl)amino)benzamide (**L3**). Yield 78.2%. ^1^H NMR (400 MHz, CDCl_3_) *δ* 9.67 (s, 1H), 7.86 (d, *J* = 1.5 Hz, 1H), 7.53 (d, *J* = 8.3 Hz, 1H), 7.38 (d, *J* = 8.9 Hz, 1H), 7.28 (d, *J* = 8.1 Hz, 1H), 7.22 (dd, *J* = 8.2, 1.5 Hz, 1H), 7.02 (d, *J* = 2.5 Hz, 1H), 6.97 (s, 1H), 6.90 (dd, *J* = 8.9, 2.5 Hz, 2H), 6.84 (t, *J* = 2.2 Hz, 1H), 6.66 (dd, *J* = 8.3, 1.9 Hz, 1H), 5.83 (brs, 2H), 3.85 (s, 3H), 3.82 (s, 3H). ^13^C NMR (101 MHz, CDCl_3_) *δ* 171.33, 160.71, 156.14, 155.55, 150.13, 146.60, 142.40, 134.58, 130.16, 129.43, 128.93, 115.43, 114.14, 113.82, 113.65, 111.84, 111.49, 108.94, 106.81, 103.44, 103.31, 55.90, 55.35. HRMS (ESI-TOF) m/z: [M+H]^+^ calcd for C_23_H_20_N_2_O_4_, 389.1496; found, 389.1496.

4-(7-Methoxybenzofuran-2-yl)-2-((3-methoxyphenyl)amino)benzamide (**L4**). Yield 79.9%. ^1^H NMR (500 MHz, CDCl_3_) *δ* 9.67 (s, 1H), 7.90 (s, 1H), 7.54 (d, *J* = 8.2 Hz, 1H), 7.31 (d, *J* = 8.1 Hz, 1H), 7.28 (s, 1H), 7.15 (q, *J* = 7.6 Hz, 2H), 7.01 (s, 1H), 6.88 (d, *J* = 7.8 Hz, 1H), 6.86 (s, 1H), 6.82 (d, *J* = 7.0 Hz, 1H), 6.64 (d, *J* = 7.9 Hz, 1H), 5.84 (s, 2H), 4.03 (s, 3H), 3.83 (s, 3H). ^13^C NMR (101 MHz, DMSO-*d*_6_) *δ* 171.24, 160.89, 154.78, 145.85, 145.36, 143.90, 142.96, 133.26, 130.81, 130.67 (2C), 124.61, 117.71, 114.80, 113.92, 112.70, 110.78, 108.82, 108.00, 105.80, 104.47, 56.25, 55.51. HRMS (ESI-TOF) m/z: [M+H]^+^ calcd for C_23_H_20_N_2_O_4_, 389.1496; found, 389.1498.

4-(6-Chlorobenzofuran-2-yl)-2-((3-methoxyphenyl)amino)benzamide (**L5**). Yield 53.9%. ^1^H NMR (400 MHz, DMSO-*d*_6_) *δ* 10.17 (s, 1H), 8.16 (s, 1H), 7.84 (d, *J* = 8.3 Hz, 1H), 7.81 (s, 1H), 7.77 (d, *J* = 1.4 Hz, 1H), 7.68 (d, *J* = 8.3 Hz, 1H), 7.56 (s, 1H), 7.52 (s, 1H), 7.35 (dd, *J* = 8.3, 1.4 Hz, 1H), 7.32 (dd, *J* = 8.4, 1.8 Hz, 1H), 7.27 (t, *J* = 8.1 Hz, 1H), 6.83 (d, *J* = 8.0 Hz, 1H), 6.80 (d, *J* = 2.0 Hz, 1H), 6.64 (dd, *J* = 8.2, 1.9 Hz, 1H), 3.77 (s, 3H). ^13^C NMR (101 MHz, CDCl_3_) *δ* 170.28, 159.68, 154.59, 153.97, 145.61, 141.22, 132.97, 129.54, 129.14, 127.95, 126.49, 122.88, 120.57, 114.64, 113.06, 112.68, 110.86, 110.46, 107.91, 105.94, 101.90, 54.30. HRMS (ESI-TOF) m/z: [M-H]^-^ calcd for C_22_H_17_ClN_2_O_3_, 391.0855; found, 391.0863.

4-(5-Fluorobenzofuran-2-yl)-2-((3-methoxyphenyl)amino)benzamide (**L6**). Yield 91.3%. ^1^H NMR (400 MHz, CDCl_3_) *δ* 9.67 (s, 1H), 7.85 (s, 1H), 7.54 (d, *J* = 7.7 Hz, 1H), 7.41 (dd, *J* = 8.8, 3.7 Hz, 1H), 7.29 (d, *J* = 8.0 Hz, 1H), 7.22 (d, *J* = 8.1 Hz, 2H), 7.08 – 6.95 (m, 2H), 6.90 (d, *J* = 7.7 Hz, 1H), 6.83 (s, 1H), 6.66 (d, *J* = 8.1 Hz, 1H), 5.85 (s, 2H), 3.82 (s, 3H). ^13^C NMR (101 MHz, CDCl_3_) *δ* 171.26, 160.74, 159.36 (d, ^1^*J_CF_* = 238.4 Hz), 156.56, 151.28, 146.67, 142.30, 134.13, 130.19, 129.65 (d, ^3^*J_CF_* = 10.8 Hz), 128.98, 115.75, 114.19, 113.73, 112.61 (d, ^2^*J_CF_* = 26.5 Hz), 112.00 (d, ^3^*J_CF_* = 9.6 Hz), 111.64, 108.98, 106.97, 106.54 (d, ^2^*J_CF_* = 25.1 Hz), 103.33 (d, *J* = 4.1 Hz), 55.35. HRMS (ESI-TOF) m/z: [M-H]^-^ calcd for C_21_H_15_ClN_2_O_3_, 375.1150; found, 375.1159.

2-((3-Chlorophenyl)amino)-4-(6-hydroxybenzofuran-2-yl)benzamide (**L7**). Yield 83.2%. ^1^H NMR (400 MHz, DMSO-*d*_6_) *δ* 10.18 (s, 1H), 9.69 (s, 1H), 8.14 (s, 1H), 7.82 (d, *J* = 8.3 Hz, 1H), 7.69 (s, 1H), 7.55 (s, 1H), 7.44 (d, *J* = 8.4 Hz, 1H), 7.36 (dd, *J* = 9.3, 6.5 Hz, 3H), 7.25 (s, 1H), 7.20 (d, *J* = 8.1 Hz, 1H), 7.04 (d, *J* = 7.9 Hz, 1H), 6.94 (s, 1H), 6.77 (dd, *J* = 8.4, 1.8 Hz, 1H).  ^13^C NMR (101 MHz, DMSO-*d*_6_) *δ* 171.05, 156.81, 156.15, 153.03, 144.85, 143.85, 134.39, 133.76, 131.58, 130.64, 122.10, 121.89, 121.08, 119.28, 118.32, 118.09, 115.26, 113.37, 110.98, 104.50, 97.99. HRMS (ESI-TOF) m/z: [M-H]^-^ calcd for C_21_H_15_ClN_2_O_3_, 377.0698; found, 377.0703.

2-((3-Fluorophenyl)amino)-4-(6-hydroxybenzofuran-2-yl)benzamide (**L8**). Yield 85.4%. ^1^H NMR (400 MHz, DMSO-*d*_6_) *δ* 10.21 (s, 1H), 9.68 (s, 1H), 8.13 (s, 1H), 7.82 (d, *J* = 8.1 Hz, 1H), 7.71 (s, 1H), 7.54 (s, 1H), 7.49 – 7.30 (m, 4H), 7.04 (d, *J* = 8.3 Hz, 2H), 6.94 (s, 1H), 6.86 – 6.72 (m, 2H). ^13^C NMR (101 MHz, DMSO-*d*_6_) *δ* 171.11, 163.56 (d, ^1^*J_CF_* = 242.5 Hz), 156.80, 156.16, 153.06, 144.91, 144.13 (d, ^3^*J_CF_* = 10.6 Hz), 133.78, 131.56 (d, ^3^*J_CF_* = 9.7 Hz), 130.63, 122.08, 121.10, 118.15, 115.52, 115.16, 113.36, 110.92, 108.67 (d, ^2^*J_CF_* = 21.2 Hz), 106.32 (d, ^2^*J_CF_* = 24.3 Hz), 104.51, 98.02. HRMS (ESI-TOF) m/z: [M+H]^+^ calcd for C_21_H_15_FN_2_O_3_, 363.1139; found, 363.1142.

2-((3,5-Difluorophenyl)amino)-4-(6-hydroxybenzofuran-2-yl)benzamide (**L9**). Yield 78.5%. ^1^H NMR (400 MHz, DMSO-*d*_6_) *δ* 10.13 (s, 1H), 9.70 (s, 1H), 8.14 (s, 1H), 7.82 (d, *J* = 8.3 Hz, 1H), 7.75 (s, 1H), 7.58 (s, 1H), 7.44 (dd, *J* = 9.1, 3.1 Hz, 3H), 6.96 (s, 1H), 6.88 (d, *J* = 7.8 Hz, 2H), 6.77 (dd, *J* = 8.4, 1.8 Hz, 1H), 6.72 (d, *J* = 9.3 Hz, 1H).  ^13^C NMR (101 MHz, DMSO-*d*_6_) *δ* 170.77, 163.89 (d, ^1^*J_CF_* = 243.4 Hz), 163.73 (d, ^1^*J_CF_* = 243.3 Hz), 156.85, 156.20, 152.90, 145.65 (t, *J* = 13.3 Hz), 143.51, 133.75, 130.63, 122.11, 121.10, 120.10, 116.38, 113.39, 112.66, 104.63, 101.37 (d, *J* = 11.8 Hz), 101.37 (d, *J* = 28.1 Hz), 98.03, 96.51 (t, *J* = 26.3 Hz). HRMS (ESI-TOF) m/z: [M+H]^+^ calcd for C_21_H_14_F_2_N_2_O_3_, 381.1045; found, 381.1046.

**General procedure for synthesis of intermediates 9a-9b.**

To a flask containing benzene-1,2-diol (5.5 g, 50.0 mmol) was added NaOH (2.0 g, 50.0 mmol) followed by the addition of EtOH (20 mL) under Ar atmosphere. After stirring for 1 h at room teperature, the (bromomethyl)cyclopropane (7.9 g, 58.5 mmol, for **9a**) or bromocyclopentane(8.7 g, 58.5 mmol, for **9b**) was added dropwise and the mixture was refluxed at 85℃ for 24h.^3^ Then the reaction mixture was concentrated and was extracted with portions of ethyl acetate. The combined organic extracts were dried over anhydrous sodium sulfate, concentrated and purified by silica gel column chromatography to get the compounds **9a-9b**, respectively.

2-(Cyclopropylmethoxy)phenol (**9a**). Yield 41.1%. ^1^H NMR (400 MHz, CDCl_3_) *δ* 6.98 – 6.95 (m, 1H), 6.90 (dt, *J* = 2.8, 2.4 Hz, 1H), 6.88 – 6.82 (m, 2H), 5.81 (s, 1H), 3.90 (d, *J* = 7.1 Hz, 2H), 1.37 – 1.18 (m, 1H), 0.71 – 0.65 (m, 2H), 0.41 – 0.35 (m, 2H).

2-(Cyclopentyloxy)phenol (**9b**). Yield 42.6%. ^1^H NMR (400 MHz, CDCl_3_) *δ* 6.92 (dd, *J* = 7.6, 2.0 Hz, 1H), 6.86 – 6.79 (m, 3H), 5.65 (s, 1H), 4.81 (ddd, *J* = 8.5, 5.6, 3.1 Hz, 1H), 1.98 – 1.86 (m, 4H), 1.83 – 1.76 (m, 2H), 1.71 – 1.62 (m, 2H).

**General procedure for synthesis of intermediates 10a-10b.**

To a solution of **9** (600 mg, 3.4 mmol) in DCM (12 mL) was added Br_2_ (0.18 mL, 3.5 mmol, diluted with 3.0 mL dichloromethane) dropwise at -60 ^o^C for 30 min. After stirring at -60 ^o^C for 2 h, the mixture was warmed up to room temperature and quenched with saturated Na_2_S_2_O_3_ solution.^3^ Then the reaction mixture was extracted with dichloromethane and the combined organic layer was concentrated to afford the compound **10a-10b.**

4-Bromo-2-(cyclopropylmethoxy)phenol (**10a**). Yield 94.3%. ^1^H NMR (400 MHz, CDCl_3_) *δ* 7.00 (dd, *J* = 8.4, 2.1 Hz, 1H), 6.96 (d, *J* = 2.0 Hz, 1H), 6.83 (d, *J* = 8.4 Hz, 1H), 5.74 (s, 1H), 3.86 (dd, *J* = 7.0, 4.3 Hz, 2H), 1.29 (qd, *J* = 7.6, 3.7 Hz, 1H), 0.69 (q, *J* = 5.5 Hz, 2H), 0.37 (t, *J* = 5.2 Hz, 2H).

4-Bromo-2-(cyclopentyloxy)phenol (**10b**). Yield 96.2%. ^1^H NMR (400 MHz, CDCl_3_) *δ* 6.99 – 6.92 (m, 2H), 6.78 (d, *J* = 9.0 Hz, 1H), 5.57 (s, 1H), 4.78 (td, *J* = 5.5, 2.7 Hz, 1H), 1.90 (ddd, *J* = 9.3, 8.7, 4.4 Hz, 4H), 1.78 (dd, *J* = 7.2, 4.7 Hz, 2H), 1.65 (dd, *J* = 9.4, 5.6 Hz, 2H).

**General procedure for synthesis of intermediates 11a-11b.**

To a solution of **10** (3.0 mg, 11.7 mmol) in CH_3_CN:H_2_O=1:1 (118 mL) was added KOH (7.7 g, 137.1 mmol), and diethyl (bromodifluoromethyl)phosphonate (4.2 mL, 23.4 mmol) and the reaction was stirred at room temperature for 15 min.^4^ Then the mixture was diluted with ethyl acetate. The organic layer was separated, washed with saturated NaCl solution, concentrated and purified by silica gel column chromatography to get the products **11a-11b**, respectively.

4-Bromo-2-(cyclopropylmethoxy)-1-(difluoromethoxy)benzene (**11a**). Yield 96.1%. ^1^H NMR (400 MHz, CDCl_3_) *δ* 7.18 (s, 1H), 7.05 (d, *J* = 8.6 Hz, 1H), 7.04 (s, 1H), 6.58 (t, *J_HF_* = 75.3 Hz, 1H), 3.85 (d, *J* = 3.1 Hz, 2H), 1.33 – 1.23 (m, 1H), 0.69 – 0.62 (m, 2H), 0.35 (q, *J* = 5.0 Hz, 2H).

4-Bromo-2-(cyclopentyloxy)-1-(difluoromethoxy)benzene (**11b**). Yield 98.3%. ^1^H NMR (400 MHz, CDCl_3_) *δ* 7.11 (s, 1H), 7.04 (s, 2H), 6.52 (t, *J_HF_* = 75.3 Hz, 1H), 4.79 (dt, *J* = 5.8, 2.6 Hz, 1H), 2.03 – 1.85 (m, 4H), 1.87 – 1.74 (m, 2H), 1.73 – 1.60 (m, 2H).

**General procedure for synthesis of compounds L10-L13.**

The same Procedure as the synthesis of **7a**-**7i**.

2-(3-(Cyclopropylmethoxy)-4-(difluoromethoxy)phenyl)benzofuran (**L10**). Yield 47.8%. ^1^H NMR (400 MHz, CDCl_3_) *δ* 7.57 (d, *J* = 7.6 Hz, 1H), 7.51 (d, *J* = 8.1 Hz, 1H), 7.44 (s, 1H), 7.41 (d, *J* = 8.4 Hz, 1H), 7.29 (t, *J* = 7.6 Hz, 1H), 7.22 (d, *J* = 8.4 Hz, 2H), 6.98 (s, 1H), 6.67 (t, ^2^*J_HF_* = 75.4 Hz, 1H), 3.99 (d, *J* = 6.9 Hz, 2H), 1.37 – 1.29 (m, 1H), 0.67 (q, *J* = 6.4 Hz, 2H), 0.40 (q, *J* = 5.0 Hz, 2H). ^13^C NMR (101 MHz, CDCl_3_) *δ* 154.89, 150.80, 140.58, 129.11, 129.09, 124.49, 123.09, 123.00, 120.98, 117.92, 116.18 (t, *J_CF_* = 259.9 Hz), 111.16, 110.92, 101.70, 99.99, 74.09, 10.19, 3.24. HRMS (ESI-TOF) m/z: [M+H]^+^ calcd for C_19_H_16_F_2_O_3_, 331.1140; found, 331.1141.

2-(3-(Cyclopropylmethoxy)-4-(difluoromethoxy)phenyl)benzofuran-6-ol (**L11**). Yield 46.1%. ^1^H NMR (400 MHz, CDCl_3_) *δ* 7.37 (d, *J* = 8.4 Hz, 1H), 7.36 (d, *J* = 1.9 Hz, 1H), 7.32 (dd, *J* = 8.3, 1.9 Hz, 1H), 7.18 (d, *J* = 8.3 Hz, 1H), 7.00 (d, *J* = 1.7 Hz, 1H), 6.87 (s, 1H), 6.78 (dd, *J* = 8.4, 2.1 Hz, 1H), 6.66 (t, *J_HF_* = 75.4 Hz, 1H), 5.32 (s, 1H), 3.95 (d, *J* = 6.9 Hz, 2H), 1.39 – 1.27 (m, 1H), 0.72 – 0.61 (m, 2H), 0.39 (q, *J* = 4.9 Hz, 2H). ^13^C NMR (126 MHz, CDCl_3_) *δ* 154.73, 153.17, 152.76, 149.65, 139.10 (t, *J* = 3.0 Hz), 128.18, 121.86, 121.70, 120.21, 116.38, 115.18 (t, *J*_CF_ = 259.9 Hz), 111.21, 109.39, 100.53, 97.25, 73.05, 9.11, 2.21. HRMS (ESI-TOF) m/z: [M-H]^-^ calcd for C_19_H_16_F_2_O_4_, 345.0944; found, 345.0952.

2-(3-(Cyclopentyloxy)-4-(difluoromethoxy)phenyl)benzofuran (**L12**). Yield 34.8%. ^1^H NMR (400 MHz, CDCl_3_) *δ* 7.57 (d, *J* = 7.6 Hz, 1H), 7.52 (d, *J* = 8.0 Hz, 1H), 7.45 (d, *J* = 1.3 Hz, 1H), 7.37 (dd, *J* = 8.3, 1.5 Hz, 1H), 7.32 – 7.26 (m, 1H), 7.25 – 7.22 (m, 1H), 7.21 – 7.14 (m, 1H), 6.97 (s, 1H), 6.58 (t, ^2^*J_HF_* = 75.5 Hz, 1H), 4.99 – 4.89 (m, 1H), 2.03 – 1.91 (m, 4H), 1.90 – 1.80 (m, 2H), 1.74 – 1.61 (m, 2H). ^13^C NMR (101 MHz, CDCl_3_) *δ* 154.91, 150.81, 140.62, 129.12, 129.10, 124.50, 123.10, 123.01, 120.99, 117.93, δ 116.19 (t, *J_CF_* = 260.0 Hz), 111.17, 110.93, 101.71, 100.00, 74.09, 10.19, 3.24. HRMS (ESI-TOF) m/z: [M+H]^+^ calcd for C_20_H_18_F_2_O_3_, 345.1297; found, 345.1299.

2-(3-(Cyclopentyloxy)-4-(difluoromethoxy)phenyl)benzofuran-6-ol (**L13**). Yield 42.1%. ^1^H NMR (400 MHz, CDCl_3_) *δ* 7.39 (d, *J* = 7.8 Hz, 1H), 7.38 (s, 1H), 7.31 (d, *J* = 8.1 Hz, 1H), 7.18 (d, *J* = 8.2 Hz, 1H), 7.01 (s, 1H), 6.89 (s, 1H), 6.78 (d, *J* = 8.2 Hz, 1H), 6.57 (t, *J_HF_* = 75.6 Hz, 1H), 5.13 (s, 1H), 4.93 (s, 1H), 1.95 (s, 4H), 1.84 (d, *J* = 4.2 Hz, 2H), 1.67 (s, 2H). ^13^C NMR (126 MHz, CDCl_3_) *δ* 155.79, 154.36, 153.77, 149.84, 140.67, 129.18, 123.19, 122.78, 121.22, 117.06, 116.33 (t, *J_CF_* = 259.3 Hz), 112.22, 111.27, 101.52, 98.31, 80.82, 32.83, 23.93. HRMS (ESI-TOF) m/z: [M-H]^-^ calcd for C_20_H_18_F_2_O_4_, 359.1100; found, 359.1113.
